# Human-centered participatory co-design with children and adults for a prototype lifestyle intervention and implementation strategy in a rural middle school

**DOI:** 10.1186/s12889-024-18351-x

**Published:** 2024-03-19

**Authors:** Janette M. Watkins, Sarah J. Greeven, Kathleen N. Heeter, Julia E. Brunnemer, Jacob Otile, Paola A. Fernández Solá, Sandeep Dutta, James M. Hobson, Justin M. Evanovich, Cassandra J. Coble, Nicole E. Werner, Vanessa M. Martinez Kercher, Kyle A. Kercher

**Affiliations:** 1 Department of Kinesiology, School of Public Health-Bloomington, Indiana University, Bloomington, IN USA; 2 Program in Neuroscience, College of Arts and Sciences, Indiana University, Bloomington, IN USA; 3 Department of Applied Health Science, School of Public Health-Bloomington, Indiana University, Bloomington, IN USA; 4 Department of Health & Wellness Design, School of Public Health-Bloomington, Indiana University, Bloomington, IN USA; 5 Department of Epidemiology & Biostatistics, School of Public Health-Bloomington, Indiana University, Bloomington, IN USA; 6 Neag School of Education, University of Connecticut, Storrs, CT USA; 7 White River Valley Middle School, Lyons, IN USA

**Keywords:** Human-centered design, Sport-based youth development, Sport for development, Cardiovascular disease, Multilevel intervention, Physical activity

## Abstract

**Purpose:**

The significance of regular physical activity (PA) in reducing cardiovascular disease (CVD) risk is widely acknowledged. However, children in rural areas encounter specific barriers to PA compared to their urban counterparts. This study employs human-centered participatory co-design, involving community stakeholders in developing a multi-level PA intervention named Hoosier Sport. The primary hypothesis is the co-design sessions leading to the development of a testable intervention protocol.

**Methods:**

Two co-design teams, each consisting of six children and six adults, were formed using human-centered participatory co-design facilitated by research faculty and graduate students. The process involved five co-design sessions addressing problem identification, solution generation, solution evaluation, operationalization, and prototype evaluation. Thematic analysis was employed to identify key themes and intervention components.

**Results:**

Child co-designers (*n* = 6) ranged from 6th to 8th grade, averaging 12.6 years (*SD* = 1.8), while adult co-designers (*n* = 6) averaged 43.3 years (*SD* = 8.08). Thematic analysis revealed children emphasizing autonomy, the freedom to choose physical and non-physical activities, and the importance of building peer relationships during PA. Adult interviews echoed the importance of autonomy and choice in activities, with a focus on relatedness through positive role modeling.

**Conclusion:**

The prototype intervention and implementation strategies developed constitute a testable intervention aligned with Phase 1 of the ORBIT model. This testable prototype lays the groundwork for a collaborative campus-community partnership between the university and the local community, ensuring mutual benefits and sustainable impact.

**Supplementary Information:**

The online version contains supplementary material available at 10.1186/s12889-024-18351-x.

## Introduction

Cardiovascular disease (CVD) is the leading cause of death in the United States (U.S.), with people from rural areas and lower socioeconomic backgrounds facing a higher risk of CVD [1–4]. Despite the disease burden, several risk factors for developing CVD can be modified. Regular participation in physical activity (PA) is a well-recognized modifiable behavior effective in reducing the risk of CVD. Past research has shown that when children develop PA behaviors during childhood, they are more likely to continue these behaviors into adulthood. However, only one out of five children in the U.S. engage in the recommended levels of PA [5]. Additionally, because the progression of atherosclerosis begins in childhood, prevention strategies are critical for establishing lifelong PA-related behaviors [6–8].

Lack of PA is a concern for children from a diverse range of backgrounds, with only 21% of children meeting the World Health Organization (WHO) PA guidelines [9]. However, those residing in rural areas encounter unique challenges in participating in PA in contrast to their urban counterparts. Many of the barriers to PA experienced by children in rural areas stem from lower socioeconomic status (SES) impeding their access to PA resources. Specifically, reduced SES can curtail PA opportunities due to the limited availability of sports equipment [10], fewer parental role models for PA [11], and overall reduced parental support for being physically active [12]. Additionally, lack of transportation poses a significant barrier for children in low SES communities due to barriers to attending sport/PA programs and accessing public spaces that support PA (e.g., parks, training facilities) [13, 14]. This limitation not only hinders their access to educational opportunities but also restricts their ability to participate in extracurricular activities, including sports and physical activities, creating disparities in health and psychosocial development.

Preserving rural health depends, in part, on the implementation of policies that address the socioeconomic disadvantage within schools. These policies should focus on providing essential health-related initiatives for PA promotion (e.g., incorporating PA breaks and funding programs) as well as access to healthcare, and transportation, all of which are crucial for enhancing the health and well-being of children [15, 16]. Moreover, children from lower socioeconomic households often face social stigma, feel unwelcome on school teams, lack prerequisite sports equipment/apparel, and deal with financial hardships [17]. Physical activity interventions in structured settings (e.g., school, summer camps, PA/sport programs) have shown limited success in enhancing population health and influencing PA trends, especially when progressing to larger scale trials [18, 19]. This lack of translation highlights the need for improved interventions and implementation strategies, especially in light of the ongoing challenges associated with rural participation in PA.

College student role modeling serves as a powerful intervention for providing the knowledge, skills, and values needed to support children’s health behaviors [20–22]. Children perceive young adults as more credible and relatable than older adults [23–25]. Young adults better understand the concerns of young children and effectively convey messaging about PA through interpersonal relationships, such as role modeling [23–25]. This, in turn, increases the likelihood of behavior change [23]. Additionally, incorporating trained college student mentors as the primary facilitators of intervention/programs can improve cost-effectiveness and sustainability [26]. College students as implementers ensures a continuous influx of new students, reducing overall staffing costs. In community-engaged research, community stakeholders often express dissatisfaction with programs. Frustration arises because the programs/interventions are typically short-lived, provide limited long-term benefits, and lack the necessary infrastructure to support sustained efforts [27]. To address these concerns, an implementation plan will be designed utilizing college student mentors and adopting a long-term approach to collaborate with the community. This strategy emphasizes building capacity through the continuous development of college student mentors and the delivery of ongoing interventions and programming.

Because rural populations bear a higher burden of CVD [28], there is an increasing demand for innovative multilevel interventions in under-resourced community settings. A multilevel intervention employs a comprehensive strategy to address health disparities, focusing on individual, interpersonal, and community levels. Following the Obesity-Related Behavioral Intervention Trials (ORBIT) model [29] is a systematic framework recommended to establish a robust evidence-based research foundation, increasing the likelihood of implementation of multilevel interventions. The ORBIT model provides a flexible and iterative process for behavioral intervention development and testing that can help with design of early-stage interventions, refine interventions if they fail in early stages, or push interventions toward rigorous testing when they achieve success in early testing [29]. Specifically, goal of Phase I of the ORBIT model is to design the essential features of a behavioral treatment [29]. Moreover, conducting research with children as part of the design team is crucial for gaining a well-rounded understanding of the specific PA-based needs within the context of schools. A recent analysis of child-focused health research revealed a significant gap in that less than 1% of published studies involve input from children throughout the research process [30]. Despite acknowledging that children offer unique perspectives and ideas not always accessible to adult researchers, their involvement remains minimal [30–32].

Therefore, we conducted a prospective single site study using a co-design process to collaboratively design a lifestyle intervention protocol and implementation strategies to align with middle school stakeholder preferences and community context. Specifically, a human-centered participatory co-design was used to take a systematic approach to understanding context, preferences, barriers, and facilitators that puts community stakeholders at the center of the intervention development and testing process [33, 34]. The present study was conducted within Phase Ia of the ORBIT model to design the scientific foundation and essential features of *Hoosier Sport*, a sport-based youth developmental program. Specifically, we engaged separate design teams of children and adults from the target community in co-design sessions. The study’s objective was to collaboratively design a testable prototype multi-level lifestyle intervention and implementation strategy named *Hoosier Sport* through co-designing with both children and adults.

## Methods and analysis

The subsequent sections delineate the conceptual framework and methodology used in this study. Within this study, physical activity (PA) was defined in accordance with the Centers for Disease Control and Prevention as: *any bodily movement that is produced by the contraction of skeletal muscle and that substantially increases energy expenditure* [35]. The formation of separate design teams of children and adults was done intentionally to enhance inclusivity and adaptability of *Hoosier Sport* and to encourage children to share openly with the primarily college student research team.

### Conceptual framework

**Fig. 1:**
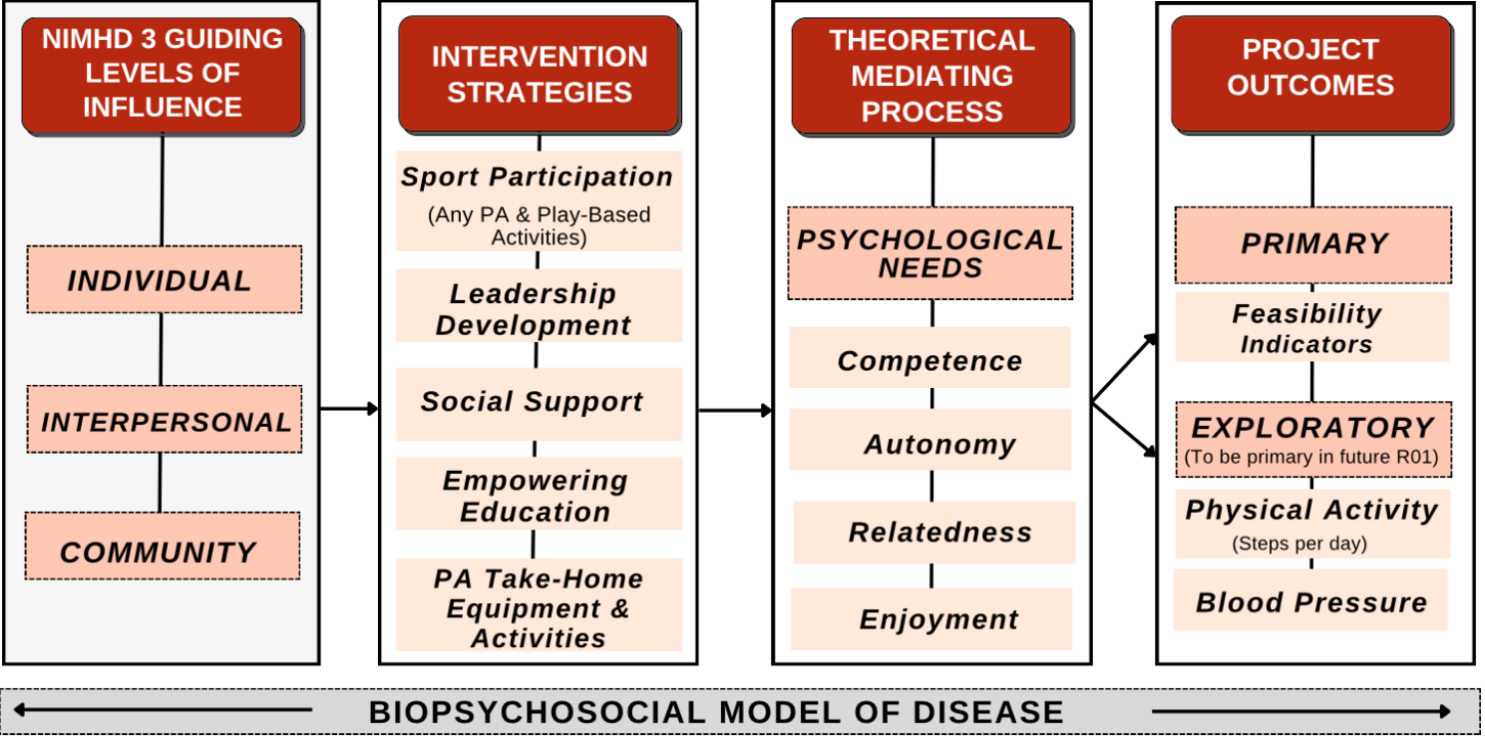
Hoosier sport conceptual model

This study’s conceptual underpinning rests upon three interrelated theoretical elements— (1) the basic psychological needs mini-theory rooted in self-determination theory (SDT) [36], (2) the biopsychosocial model [37], and (3) the National Institute on Minority Health and Health Disparities (NIMHD) research framework [38]. Each of these conceptual models guided the development of the methodology. The basic psychological needs mini-theory aided the research team in predicting and examining factors that influence study outcomes. Meanwhile, the biopsychosocial model assisted in describing and interpreting the broad range of findings, although it does not specifically predict outcomes. Lastly, the NIMHD Research Framework helped conceptualize the multilayered factors essential in comprehending and enhancing physical activity within our rural, low-socioeconomic context. These four theoretical elements are described in greater detail in Fig. 1.

The present study encompassed three essential theoretical elements shaping its methodology and approach. First, the basic psychological needs mini-theory within self-determination theory highlights that enhancing autonomy, competence, and relatedness boosts child well-being [36, 39–41]. For example, incorporating open-ended questions probing these aspects along with enjoyment (pivotal for intrinsic motivation and sustained engagement in activities) [42]. In addition to the typical three psychological needs, we included the element of enjoyment to guide our approach and comprehension during the study. The integration of enjoyment within the framework of self-determination theory holds significant importance, serving as a motivational force that nurtures intrinsic motivation and sustains individuals’ involvement in activities. Ultimately, this contributes to enhancing their feelings of autonomy, competence, and relatedness [42, 43].

Secondly, the Biopsychosocial Model acknowledges the interconnectedness of biological, psychological, and social factors in shaping PA and well-being [37]. This model influenced our guiding questions and contextual framing of results, enabling considerations for biological/physical outcomes, psychological aspects, and social support strategies within the PA context.

Lastly, aligning with the NIMHD Research Framework, which addresses multiple levels of influence (individual, interpersonal, and community) [38], our study targeted multilevel impact. Understanding the complex barriers faced by rural communities in PA, this framework directed our discussions within co-design teams toward achieving impact across various levels rather than solely focusing on individual behaviors. These theoretical underpinnings significantly influenced our participatory co-design session agendas, design team discussions, and interpretation, enhancing its depth and breadth within the study.

### Design

**Fig. 2:**
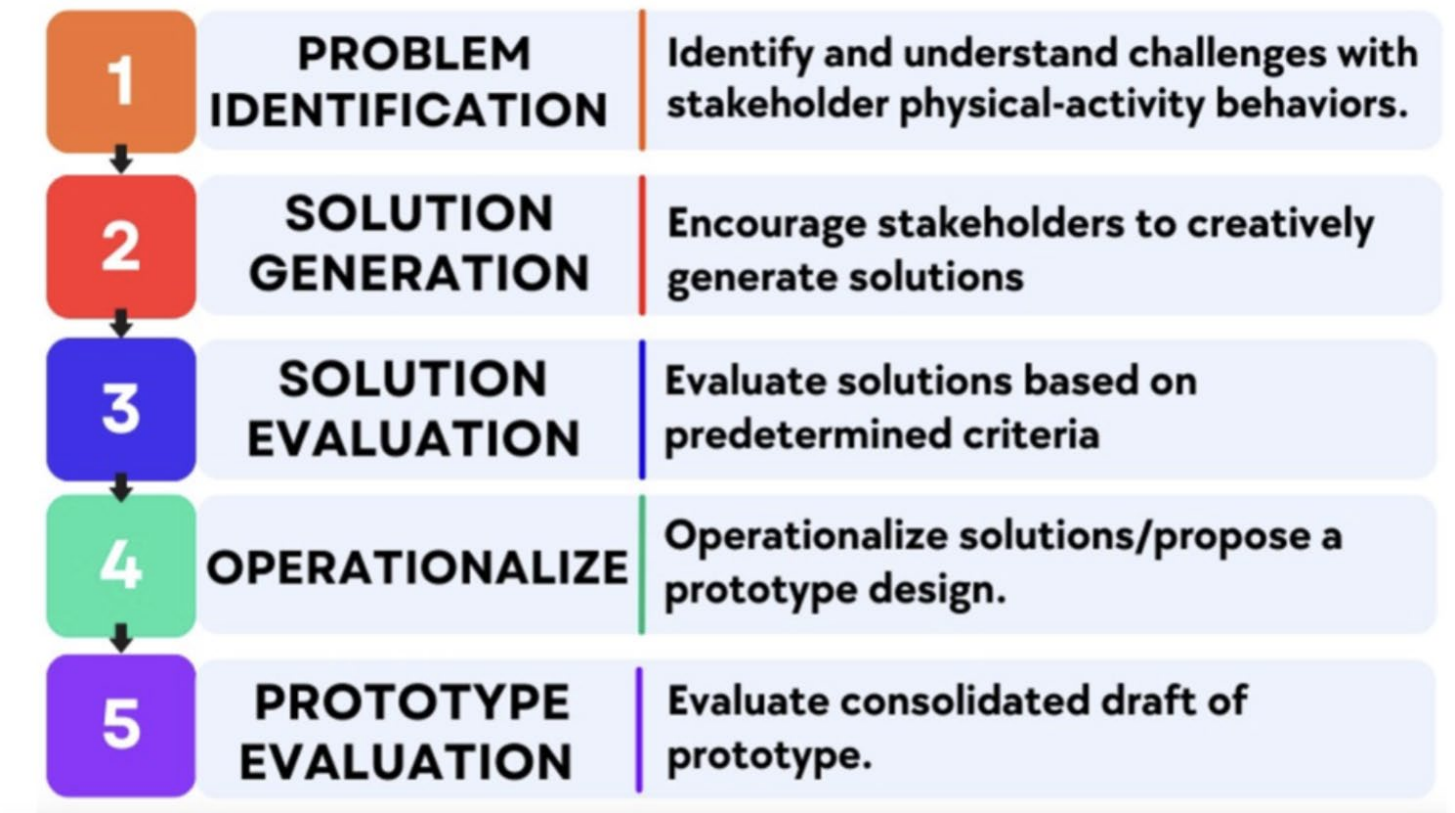
Co-design session goals

We conducted a 5-step participatory co-design protocol that includes the following five session sequence: (1) problem identification; (2) solution generation; (3) solution evaluation; (4) operationalization; and (5) prototype evaluation. The participatory co-design process in our study context was designed to empower children and adults (i.e., parents/teachers/administrators) to provide input into the prototype *Hoosier Sport* lifestyle intervention protocol and implementation strategies. The goals of these sessions are outlined in Fig. 2. Based on preliminary school stakeholder input [44] and previous lifestyle intervention literature [45], the five preliminary topics we selected to guide design sessions were: (1) sport/PA participation [46]; (2) leadership development [47]; (3) social support for PA [48]; (4) empowering education [49]; (5) PA take-home equipment & activities [50]. School administrators requested that the *Hoosier Sport* intervention be designed to be conducted primarily during physical education class but we also discussed other before-, during-, and after-school programming ideas. *Hoosier Sport* was selected because *Hoosier* is a term of pride among many Indiana residents and integrating *sport* into the intervention is part of the “hook” to encourage children to participate in programming.

The published participatory co-design approach employed by the present study has been shown to lead to effective intervention development [51–54]. Co-designed interventions are likely to be more engaging, satisfying, and useful to participants [55], and while co-design has been done in under-resourced PA contexts with children [56, 57], the field remains in its relative infancy. Our planned methods will consider the unique PA-related needs, goals, opportunities, and assets of rural children, parents, and teachers/administrators and may be more likely to lead to PA-based intervention protocol and implementation strategies that are uniquely responsive to the target middle school community.

### Setting and sample

The co-design teams were made-up of faculty and student researchers (*n* = 6) and children (*n* = 6) or researchers (*n* = 6) and adults (parents, teachers) (*n* = 6) from the middle school community. This sample size was chosen using the recommended group size for participatory design [58]. The target population was 6th– 8th and grade students (generally 10–13 years old). The group sizes aligned with selecting a group that was mature enough for more advanced intervention strategies than elementary school students, while also aligning with our research team’s expertise. The odd number of participants allowed for a majority vote to break ties between design alternatives within the group. Adults and children were recruited using convenience sampling via parent/guardian meetings and weekly newsletters distributed by school administrators. To be eligible for inclusion, children had to be: (1) enrolled in the middle school; (2) entering 6th– 8th grade; (3) have parent/guardian consent to participate; and (4) willing to participate in all 5 co-design sessions. To be eligible for inclusion, adults had to be: (1) a parent/guardian of a student currently enrolled at the school in 6th– 8th grade or a teacher/administrator employed at the school; and (2) willing to participate in all five co-design sessions.

### Procedure

The two design teams completed a series of five co-design sessions across three months, with approximately one to two weeks between sessions. Child and adult participants received a $40 e-gift card for each session (earning up to $200). The adult group began the process, and in parallel, the child group alternated sessions between the adult group (e.g., adult session, child session, adult session). The two groups collaborated with the study team separately, however during their discussions, topics from other team’s conversations were also mentioned. This parallel and alternating co-design process allowed the children to have a sense of autonomy in the process, ensuring the inclusion of important concepts to them (e.g., fun, enjoyment). Simultaneously, it permitted the adults to share their opinions and ideas while discussing valuable insights from the child group.

The sessions were facilitated by an experienced research team member with training in facilitating group coaching and discussions. The research team formulated open-ended questions tailored to each session’s co-design objectives, resulting in ten semi-structured co-design sessions. Separate sessions were conducted for adults and children from the community, forming distinct groups for adults and children, respectively, to participate in these co-design sessions. For instance, in session one, the design session agenda focused on understanding challenges with children’s PA-related behaviors. The design process was an iterative process where they began by coming to a common understanding of the challenges with PA-related behaviors, then collaboratively developed numerous divergent solution ideas. Our initial strategy was to focus on five key elements informed by prior literature: (1) encouraging sport and physical activity participation, (2) cultivating leadership development, (3) fostering social support for physical activity, (4) delivering empowering education, and (5) providing take-home equipment and activities for physical fitness. Furthermore, one general predetermined component of the implementation strategy was the involvement of college students as implementers of future interventions. Co-design of the details of the college student-driven implementation strategy were discussed throughout the co-design process.

Next, we progressively moved toward a detailed and high-fidelity intervention protocol as well as identification of implementation strategies. Throughout the session, the facilitators encouraged discussions, interpretations, and respectful debates among design team members while ensuring progress. PA-based needs, goals, opportunities, and assets that were identified in a preliminary needs assessment survey [44]. were integrated throughout the design session discussions.

The research team collected observation notes and audio recordings to analyze the design teams’ work as it was produced and at the end of the final co-design session. These records captured the co-design sessions, enabling a detailed examination of the participants’ conversations, opinions, and collaborative efforts in generating intervention and implementation design solutions. Throughout the co-design sessions, the facilitators assisted in guiding the participants’ conversations and thought processes to generate and collaborate on intervention protocol design solutions. Each session lasted for 50–60 min.

### Measures

A survey of all intervention and implementation ideas was sent out to all participants after the solution evaluation (session 3). Participants rated each solution on a 1–10 scale of *Not at all important* to *Extremely important*. The results from this survey were used to help inform subsequent discussions on operationalizing ideas (session 4).

### Data analysis

The research team transcribed co-design sessions to assist in a deductive thematic data analysis using the audio recording and observation notes of the sessions. Two research team members (K.K., S.G.), both of whom were present at every design session, independently read initial transcripts, coded them for potential themes and sub-themes, and then came together with a third research team member (K.H.) to refine and come to consensus on finalizing a codebook. Self-determination theory’s basic psychological needs satisfaction mini theory guided the transcript coding process. Themes and sub-themes were reviewed and agreed upon by four research team members (J.W., S.G., K.H., K.K.). Next, two research team members (J.W., S.G.) separately and independently reviewed, identified patterns, and applied the codebook themes and sub-themes to all the transcripts. They came together to review and refine through consensus discussion with a third research team member (K.H.). The conclusion of the qualitative analysis yielded a testable intervention protocol and implementation strategies to pilot/feasibility test in future studies.

## Results

### Sample

Child co-designers were in 6th-8th grade (*n* = 6), with a mean age of 12.6 years (SD = 1.8), while adult co-designers (*n* = 6) had a mean age of 43.3 years (SD = 8.08). The results are guided by the psychological needs satisfaction mini theory from self-determination theory (SDT), which comprises autonomy, competence, and relatedness as key themes. Furthermore, the analysis delves into additional themes related to policy, systems, and environment (PSE) to evaluate possible modifications that could enhance support for SDT within the realm of physical activity. Enjoyment was identified as a recurring theme, as enjoyment is a critical component of engaging children in PA [59, 60]. See Supplemental File 1 for all themes and sub-themes, and Supplemental File 2 for all transcripts of sessions (with pseudonyms for participants).

### Autonomy

Adults observed the significance of autonomy in physical activity (PA) settings, emphasizing discussions around the choice of PA options and alternative non-PA choices. They pointed out that students with low physical literacy might feel uneasy participating in competitive games, suggesting that providing additional avenues for participation would boost students’ autonomy and their capacity to engage in physical activities. Faculty members also spoke of the unique interests of the community, and how the intervention could promote these individual interests in the context of sport and PA.

"*So some kiddos, I’ve noticed in the past, some kiddos that are not very great at sports, they’re really good at designing like a game. So why can’t we have them design the game or some facts or whatever. And they like do trials with the kiddos that actually want to do them*.”– Teacher.

Similarly, children also emphasized autonomy in their co-design sessions. Autonomy was the most discussed theme. Children highlighted the importance of having the ability to choose between different PA programming options, and the ability to participate in non-PA options.

*"I think what my, you know, dream gym would be like, I think it would have, you know, like everybody, you know, participating, and, you know, like whatever we have, maybe some basketball goals, different toys for other kids to play with, stuff like that.”–* Child.

### Competence

The concept of competence was explored concerning skill development in sports. The goal is to enhance children’s competency in accomplishing complex tasks by breaking them down into small, tangible steps. Faculty suggested that building this competency could have a lasting impact on lifelong leisure skills, enabling children to participate in a wider range of sports opportunities within community settings.

"*But pickleball is something that my daughter, one of my daughters has learned how to play, because it’s really big in Lafayette [and can now play with others in the community]*.”– Parent.

Children highlighted the importance of competence by expressing a desire for additional resources at school, specifically after-school sports activities. They expressed a need for more structured PA opportunities after school and a wish for increased chances to be active while waiting to go home. These children articulated a need for more structured opportunities for PA post-school hours, underscoring their intrinsic motivation to engage in activities where they feel competent and capable.

"*But we sometimes go to the gym and play with like the […] teacher. They take us to the gym, and we can play. And we either try to find a game, or we have free time. [But nothing is structured.*]”– Child.

*I play tons of sports, so I usually play sports after school.*” *– Child*.

### Relatedness

Adults predominantly focused on relatedness concerning role modeling, which involved peers or adults within the school or home environment. They emphasized that role models, whether peers or adults, play a crucial role in motivating children to explore new activities and offering social support for ongoing challenges.

“*Kids would become more involved and want to do more things because they’re seeing the parents setting that example.*”– Parent.

"*[…] to have like a care mentor or like upperclassman or just kind of being a, like an aid or a mentor to other kids to get them to join in.*”– Parent.

Drawing on previous PA experiences, adults highlighted the importance of fostering improved relationships among children and adults within the school environment, especially emphasizing positive connections between children and adults in leadership roles such as coaches and parents.

"*But there was not much interaction with the coaches*.”– Parent.

"*I was thinking something even like a, maybe get all the parents together and the kids one night out of maybe that whole ordeal where you do like maybe a sack jumping race with your parents or your sibling. You know, bring them all together too, maybe just one night out of that week out of everything that you’re doing.*”– Teacher.

Children also underscored the significance of relatedness, focusing on peer relationships. Specifically, they mentioned the importance of supporting each other in small groups and pairing up with friends to enhance the enjoyment of activities. Children emphasized that PA could be an opportunity to get to know peers better and interact with different people.

"*Maybe first they could talk to each other first and get to know each other first, before they do that, before they get partnered and see if they’re compatible. If not, they can switch partners*.”– Child.

"*We could like get to know each other better, you know, say more stuff about us, say what favorite color, how many pets you have, what pets do you have, their names, stuff like that*.”– Child.

Children also pointed out a lack of effort from adults in leadership roles to support relatedness, particularly within the school setting where coaches may not promote activities that build peer connections. The children noted that, at times, teachers or coaches may not actively engage with them, limiting opportunities for relatedness in the school environment.

"*I don’t like that part when they just like put girls on one team and boys on the other so like to do like a mixture. And then at [community sports program] they put like an even amount of people on a team, and I just don’t think that’s okay*.”– Child.

"*So, I would have the teacher, or the PE teacher would actually pay attention and do stuff with the kids and stuff*.”– Child.

### Enjoyment

Adults spoke of enjoyment through the importance of inclusion. A primary concern was that some children may be unable to participate in some activities, and that the coach could do a better job of fostering an inclusive space.

"*Well, I think, and everybody should be included. Not everybody is going to be the pro sport. Not everybody is going to be the athletic, have the natural athletic ability. So if they’re talking to everybody about it’s not just about athletics, it’s about what you can do with the education, so that way, the kid that is sitting there that can’t dribble and walk at the same time isn’t going to feel left out*.”– Teacher.

Additionally, adults spoke of the importance of recognizing unique goals of children, and supporting them to increase inclusion and enjoyment of PA.

"*I like goals, as long as they’re personal goals, because the whole class, each kid isn’t going to have the same goal. As long as they can set their own goals of what they’d like to achieve, if it’s something to do with sports, what they’re, running what they want speed wise, if they want to get faster or jump higher*.”– Teacher.

Likewise, children stressed the importance of inclusion in promoting enjoyment of physical activities. These discussions focused on finding ways to include children who may not enjoy traditional sports, ensuring that everyone has a chance to participate and try something new.

"*You don’t have to be a sports person. Not everybody, like you didn’t get like dis-included from it*.”– Child.

"*I play, I want to do track, I’m going to do track this year. Soccer, I’ve always done soccer. And then I do tennis lessons, and it’s really fun. I want other kids to like want to try it too*.”– Child.

Children mentioned that the enjoyment of physical activities could be enhanced through incentives. These incentives primarily centered around group activities, recognition from adults, and the prospect of receiving prizes.

"*And then like I would like there to be water balloon fights and stuff like that, like if you earn a water balloon fight or something, you know*.”– Child.

"*Maybe like a celebration thing to like congrats us by, when we win or something, because I’m not the type of person that like asks for things, for like prizes or something. I just like to be like celebrated for stuff like that*.”– Child.

"*Maybe like, you know, maybe like, you know, maybe like if like, say that I won a game, you know. How about for a prize, how about like a like* $20 *gift card to Dairy Queen or Subway or, you know*?”– Child.

### Policy, systems, and environment (PSE)

Adults discussed potential alterations within the PSE, focusing on enhancing opportunities for children to engage in PA at school. This encompassed integrating breaks within class time for walking or outdoor activities, along with providing before- and after-school activity options.

"*I would like do like an exercise maybe in the classroom with them to kind of get them a little motivated, go outside, take a little walk or something. I mean, you don’t always have to play sports, or you don’t always have to play something, you know*.”– Teacher.

"*Basketball is with it, because it just requires a net or a hoop. Or maybe some sort of obstacle course, like the, oh, not quite the IRONMAN but, or a CrossFit type thing, running, jumping, doing all the different skills throughout the class*.”– Parent.

Adults also stressed the importance of achieving a balance between academic studies and physical activity. This entails incorporating more flexibility into schedules, enabling teachers to encourage physical activity, and requiring more PE time.

"*[…] maybe say your ABCs or something that you have memorized as you’re doing it. I don’t know, just some 10-minute, maybe 15-minute, not very long, play a boardgame […]*”– Parent.

"*Then they will go to art for a full week. So, the kids won’t rotate back into like PE. It could possibly be like four weeks before they go back into PE*.”– Teacher.

Children also highlighted the importance of expanded PA opportunities. Having breaks from class to be active and go outside were discussed.

"*But you still have a break to go outside, get fresh air and.. but as activity, we go outside sometimes, and also we have lifestyle class at the end of the day*.”– Child.

"*We have been like stuck inside, and like we like we’ve been doing like work after work, and, you know, we haven’t really got taken outside. So I think maybe we should do like 20 min in the morning and then maybe like 10 min in the afternoon*.”– Child.

Children also addressed the issue of PSE, specifically the shortage of resources in terms of qualified personnel. They shared stories of teachers who were not actively involved in class settings and stressed their desire for teachers who would actively engage with them.

"*He like, he isn’t one of those teachers like that like stay back and talk to the other teachers. He’s like one of the ones that like to do what kids are doing instead of what the adults are doing*.”– Child.

"*He’s not really strict, but he’s strict, you know, not taking advantage of him. But I like how he is very like coming in and calling out people’s names and saying, oh, hi, how’s your day? Did you get your energy going yet*?”– Child.

**Fig. 3:**
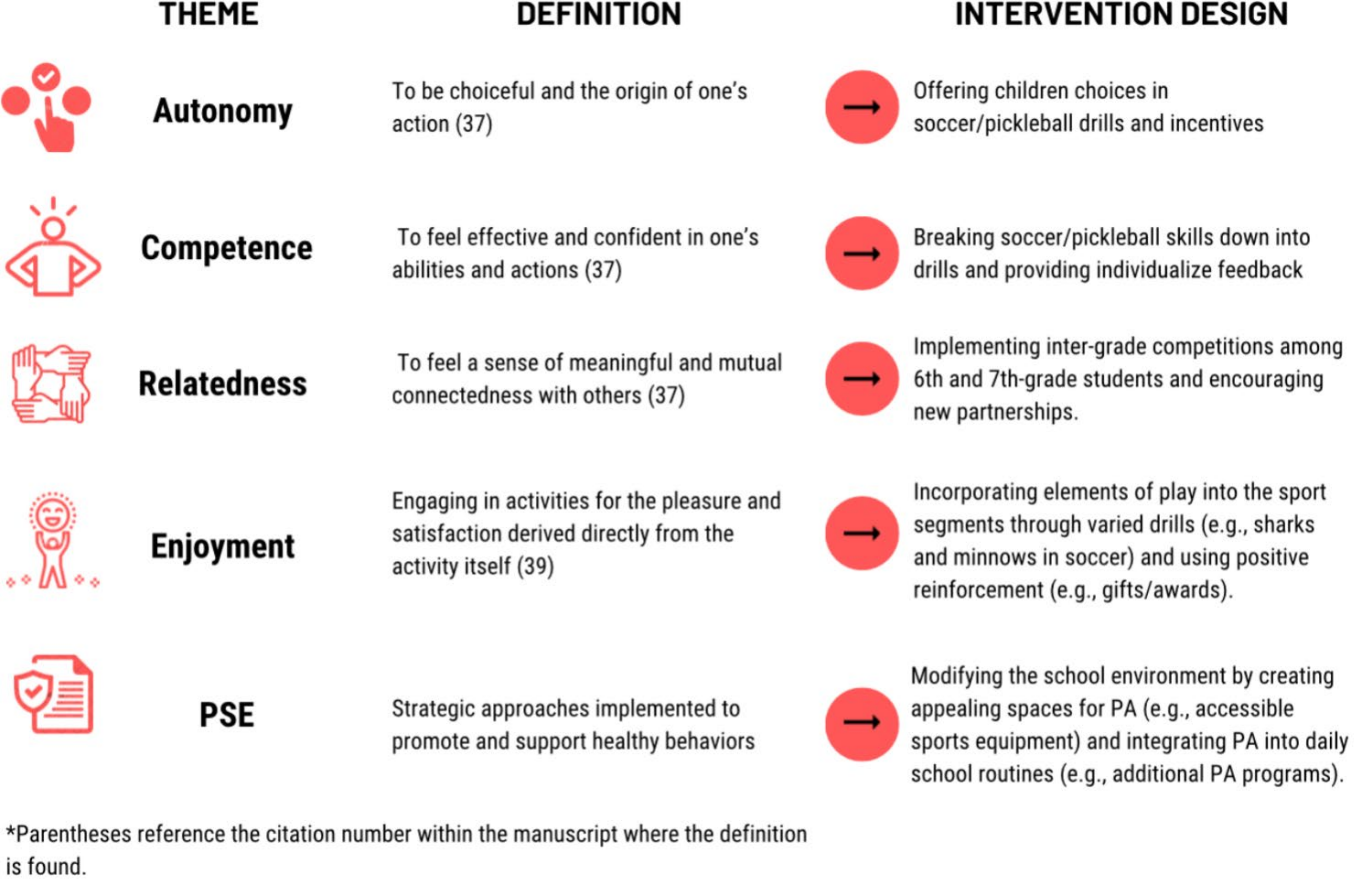
Co-design themes and intervention strategies

Finally, the designed intervention will target aspects of SDT (autonomy, competence, relatedness, and enjoyment) and PSE using the qualitative feedback from the co-design sessions as a guide. Importantly, SDT is an empirically supported framework for promoting PA in children [61, 62]. For example, to target autonomy, the *Hoosier Sport* intervention will provide choices (i.e., autonomy) in PA activities and sport drills. See Fig. 3 for how these themes were integrated into the intervention design.

## Discussion

The study’s purpose was to collaboratively design a testable prototype multi-level lifestyle intervention and implementation strategy named *Hoosier Sport* through co-design. Indeed, health interventions incorporating evidence and engaging key community members in the planning process generate more effective outcomes [34, 63, 64]. As such, the World Health Organization has recognized human-centered design as a key strategy to address various health challenges and promote equitable healthcare solutions [65]. Prior research has demonstrated that participatory co-design is an effective strategy for designing innovative interventions with unique populations (e.g., rural low-SES communities) [54, 66]. The present study revealed four key findings. First, participants highlighted the promotion of autonomy by providing choices in both PA and non-PA opportunities (in both groups/teams). Second, children and adults emphasized the importance of fostering a sense of relatedness through role modeling and leadership. Third, the study underscored the significance of increasing opportunities for structured PA during and outside of school hours. Fourth, participants identified the crucial role of support from trained facilitators/leaders in creating a healthier and more physically active environment.

Research supports the finding that encouraging autonomy fosters participation in PA [67, 68]. When students perceive autonomy from their PE teacher, such as different PA options from which they can choose (e.g., multiple stations, different games, ect), it can significantly boost their participation in physical activities [67]. Likewise, when children experience autonomy-supportive behaviors from peers and physical education teachers, they are more likely to experience a better health-related quality of life. Increased PA plays a crucial role as a mediator in this correlation [69]. Moreover, providing options for PA activities boosts perceived autonomy and increases participation among children in classroom settings [70]. Our first key finding emphasizing the importance of promoting autonomy in a rural low-SES middle school setting PA-related contexts builds on the growing body of literature that supports the notion that a sense of autonomy should be built into youth lifestyle interventions and implementation strategies.

The second key finding was a strong emphasis on the importance of fostering relatedness in school PA-related settings. Relatedness is strongly supported by self-determination theory and has demonstrated an important role for increasing PA participation among children [71, 72]. When youth experience a sense of connection with both their teachers and peers, they tend to be more inclined to participate in PA [71]. Participants reported enjoying PA more when they felt connected to their peers, saying: “I like [PA] best when I get to be with my best friends. It isn’t as much fun if you don’t know the person you’re partnered with.” Similarly, research emphasizing the significance of relationships often highlight how they enhance enjoyment, which is believed to be a contributing factor leading to increased participation in PA [72]. Importantly, when youth have high enjoyment of PA in class-settings, it correlates to a more physically active lifestyle [59]. Indeed, relatedness to peers and teachers support enjoyment, and further increase long-term participation in PA [60]. Moreover, mentorship programs involving university students have demonstrated a notable effect in boosting PA levels in youth [73]. Specifically, mentors who are perceived as more relatable or closer to the students seem to exert the most significant influence on increasing PA [74]. Likewise, peer mentorship is a feasible approach to increase PA in youth [75], demonstrating success in enhanced PA participation and levels) [76, 77]. In line with the significant body of research on psychological needs satisfaction, relatedness may play a critical role in the feasibility testing of the *Hoosier Sport* intervention and implementation strategies in subsequent pilot testing.

The third significant finding highlighted a desire for both before-, during, and after-school PA programs. Promoting PA opportunities before and after school is another empirically-based method for increasing PA in youth [19, 78]. A recent meta-analysis highlighted the significance of after-school physical activity programs as a vital tool for boosting PA and reducing sedentary behaviors [19]. Likewise, the American Heart Association recognizes schools as a crucial environment to enhance physical activity opportunities for young individuals [79], recommending before- and after-school programs as effective strategies to achieve this goal [79, 80]. School-based programs are capable of not only increasing PA levels, but also physical fitness and overall health [81]. In line with these findings and recommendations from previous literature, child and adult participants in the present study agreed with the potential for their middle school to be an appropriate setting for increasing PA. While there are significant challenges with increasing children’s PA at any time of the day, rural schools may be even more critical than urban schools due to the significant transportation and access barriers with getting rural children to PA locations outside of traditional school settings [13, 14]. Hence, consistent with prior research, offering extra PA opportunities at school could help alleviate this significant barrier to engaging in physical activity. Furthermore, participants had high competence in sport and PA participation. This may be related to reserach showing competence in physical performance is correlated with increased PA participation [82]. Indeed, physical competence has been cited as the primary psychological factor for promoting PA engagement [83]. In the present sample, children reflected confidence in their physical abilities and sought out additonal PA opportunities. Therefore, in addition to the previously discussed reasons, this community sample may especially benefit from additional PA opportunities due to their increased liklihood to participate long-term in such a program.

Just like our fourth key discovery, research strongly supports the necessity of having trained staff and teachers to facilitate opportunities for physical activity [84]. Indeed, trained staff are better equipped to influence PA levels in youth than non-trained staff [85], and staff development opportunities support increasing PA levels [86]. Both child and adult participants in the current study noted the prominent role, both positive and negative, that staff, coaches, and/or teachers can have in shaping the PA experience of middle school children. Furthermore, offering training and development to school staff can significantly enhance the effectiveness of physical activity interventions among youth [87]. In the present study population, there is currently not a licensed PE teacher, but rather an instructional assistant who is currently receiving PE training. This reflects on a key barrier to PA success within the sample and suggests that the future intervention may need to address on-site staff development to ensure sustainability of the program and outcome success.

These four findings from the present co-design study will guide the creation of a testable PA intervention called *Hoosier Sport.* This sport-based intervention aims to enhance PA engagement among youth through a multifaceted approach. Future intervention design and implementation will be guided by our preliminary studies, existing literature, and input from the community partner. Drawing from these three sources of decision making, *Hoosier Sport* emphasizes the importance of trained staff within school environments. Implementing this intervention involves providing specific training and professional development primarily to college student implementers but also to select school staff members. The implementation model focuses on the development and pilot testing of a university service-learning course that will train college student implementers to work with the *Hoosier Sport* research team to deliver interventions. Additionally, the intervention model focuses on establishing in-school, before-, and after-school programs, aligning with recommendations from the American Heart Association. Moreover, the intervention underscores the need for creating enjoyable and inclusive activities within classroom settings, fostering a strong sense of autonomy, competence, relatedness, and enjoyment in PA. Altogether, by integrating trained staff, establishing structured programs, and prioritizing key constructs of self-determination theory, *Hoosier Sport* aspires to significantly elevate rural PA participation and subsequently improve the overall health and well-being of youth. Moving forward, the *Hoosier Sport* intervention components and implementation strategy will be pilot/feasibility tested, refined, and retested in rural middle schools.

Based on this co-design study, the implementation strategy for *Hoosier Sport* will use the following: (1) training university students in a university service-learning course; (2) enhanced PE delivery [88, 89]; (3) goal setting [90]; (4) college student mentors [20, 21, 23]; and (5) a positive reinforcement system to create a sustainable PA program. Integrating a university service-learning course enables students to bridge theory and practical application by engaging in community-based projects. This hands-on approach fosters a stronger sense of social responsibility and community engagement among students. Additionally, an enhanced PE delivery model surpasses conventional methods by incorporating diverse physical activities and modern teaching techniques. Tailored to community needs, this approach not only promotes a comprehensive understanding of physical wellness but also nurtures habits conducive to lifelong health and fitness. Furthermore, collaborating with college student mentors in goal-setting processes facilitates academic and personal growth for younger students. Complementing these strategies, a positive reinforcement system within the learning environment emphasizes the value of progress and effort. By acknowledging and rewarding desired behaviors or achievements, this system cultivates a culture of continuous improvement and positivity, fostering heightened confidence and motivation.

To this end, *Hoosier Sport* will be integrated into the PE curriculum at a rural middle school. The program will be conducted twice a week over an 8-week duration, within which two 4-week segments will be dedicated to different sports, such as soccer and pickleball. Each session will encompass a structured format comprised of warm-up routines, skill-focused drills, and engaging mini-games (i.e., 3 vs. 3 soccer). In fostering participant autonomy, the program will offer diversified skill level options for drills, enabling students to choose activities tailored to their proficiency levels. This may also increase their sense of physical competency. Furthermore, participants will have the freedom to select their partners, thereby enhancing their sense of control and ownership over their learning experiences. To cultivate a sense of relatedness and teamwork, collaborative activities will be emphasized, encouraging collective goal setting that involves the entire class or large groups (i.e., as a group scoring 10 goals). Additionally, strategies aimed at fostering enjoyment will be integrated, including the gamification of activities and prioritization of drills and games preferred by the group, thus emphasizing a positive and engaging experience for all participants. Lastly, the program will extend physical activity opportunities beyond regular sessions through virtual platforms like Zoom. Service-learning students, with the assistance of school staff, will host 20-minute segments at the beginning and end of the school day. These sessions will feature a designated sport along with additional gamified PA activities such as freeze tag, obstacle courses, and relay races, offering students a diverse and engaging range of physical experiences.

The findings in this study should be interpreted within the study limitations. The study had a small sample size, relied on convenience sampling, a condensed time frame, and included primarily female representation. The use of a limited number of participants—five adults and five children in each design team—posed challenges in capturing diverse perspectives and experiences critical for a comprehensive intervention, despite being in line with recommended co-design team sizes [58]. Additionally, employing convenience sampling methods might introduce selection bias, potentially overlooking valuable insights from individuals or families not represented within the sample. Moreover, the short duration of the study, with only five co-design sessions conducted over a three-month period, may have constrained the depth and breadth of exploration in developing a robust intervention. This abbreviated time frame might have restricted the thoroughness of idea generation and limited opportunities for comprehensive refinement, potentially impacting the richness and effectiveness of the final intervention and implementation strategies.

Ultimately, the *Hoosier Sport* co-design sessions allowed for a mutually beneficial community-engaged research process which created a testable intervention and implementation strategy in our middle school partner. Indeed, this design allowed for increased feasibility and adaptability to a range of school contexts that could benefit immediately from partnerships with major academic institutions with the college student service-learning workforce to deliver programming at scale.

### Electronic supplementary material

Below is the link to the electronic supplementary material.

Supplementary Material 1

Supplementary Material 2

## Data Availability

All data generated or analyzed during this study are included in this published article [and its supplementary information files].
