# Supplementary material for: Human-centered participatory co-design with children and adults for a prototype lifestyle intervention and implementation strategy in a rural middle school
Source: BMC Public Health. 2024 Mar 19;24:845. doi: 10.1186/s12889-024-18351-x (PMC10949632; doi:10.1186/s12889-024-18351-x)
Supplement: Supplementary file 1 — Supplementary Material 1 [file 12889_2024_18351_MOESM1_ESM.docx]

Indiana University Sport Management

Hoosier Sport

Child Design Session 1

**MODERATOR 1:** Cool. So first question is about physical activity and physical education, PE class. So I asked you, Riley and Leah, just a little bit, but how do you feel about PE currently, like what you did last year, for example. We’re wondering what you like about it, one, and what you didn’t like about it. So who, Leah, would you be willing to share first, just some stuff you like and don’t like about PE?

**LEAH:** Yeah, sure. First thing I do like is we like to do dodgeball, and we get a lot of physical activity in that circle and everything. Same with like baseball and everything. Sometimes we have free days. I don’t really care for those, because I like doing dodgeball and stuff like that, but . . .

**MODERATOR 1:** Awesome. Great answer. So those are some fun things. Good. We’ll come back to you a little later. Riley or Taylor, can you tell me some stuff you like about PE?

**RILEY:** [Inaudible, 00**:**01**:**17]. Oh, sorry.

**TAYLOR:** I really like same like Leah said. I mean, I like playing dodgeball and playing basketball. And I just like get a lot of physical activity doing softball or baseball or basketball or soccer even.

**MODERATOR 1:** Cool. And soccer. Cool. Lots of physical activity. Good. How about you, Riley?

**RILEY:** Mostly dodgeball and soccer.

**MODERATOR 1:** Nice. Staying on what you guys like about it, and we’ll, like we could just go back to you, Leah. So I got some of the specific sports you guys like. How about some other things you like? Like, what, I don’t know if it’s organized well, or if you get to play those sports with a lot of kids or not, or I don’t know if the equipment is good or not. Are there some other things that you like about PE class?

**LEAH:** So I think before we do anything, we should like maybe do like a run around the gym first and then do it so we could have more physical activity.

**MODERATOR 1:** Nice. Yeah, a little warmup.

**LEAH:** And so, and also the playground outside is completely tore apart. I wish we had better equipment. There’s no, the slide is broken. There’s no swings and stuff like that. That’s why we’re all like mad when we go to recess, because we don’t have anything to slide on or to swing on, but . . .

**MODERATOR 1:** Great point, Leah. Yeah. That would be frustrating. How about Riley or Taylor? Some other things you like beyond just the sport like the organization, the equipment, that kind of stuff.

**TAYLOR:** Well, I like kind of like every, like all different kinds of physical activity. You know, like we go out to recess and, you know, we do all different kinds of physical activity. And same with the playground, you know, there’s like no, there’s barely anything kids can play on and, you know.

**MODERATOR 1:** Yeah. That makes sense. And hello, new person. What’s your name? Thanks for coming, by the way. I’m guessing it’s either Maya, Morgan, or Casey. But in the meantime, Riley, how about you? What else for things you like around physical activity and PE . . . hello, Samsung. Thanks for coming. Is that Maya, Morgan, or Casey?

**MAYA:** Maya.

**MODERATOR 1:** Maya. Awesome. Cool. Thanks for coming. Maya. Cool. Maya, you in, are you in eighth grade?

**MAYA:** Yep.

**MODERATOR 1:** Perfect. Okay. Cool. So let me catch you up real fast. All you’ve missed is we introduced ourselves. We talked about the program, which is called Hoosier Sport, which we’re coming to WRV to try to start a physical activity and sport-based program at the school. At first, it’s going to be in PE class and a little bit after school, probably one day a week after school and then in a couple of PE classes. It’ll start in October or November. And really briefly, you’ll get paid after each of these sessions, $40, either an Amazon eGift card or a physical VISA card.

We’re recording these sessions, but we’re going to remove any identifiable information like names, that kind of stuff. We’re recording them so that we can like not miss any of the awesome information you guys are giving us. We want like a, so if we were taking notes, we’d miss all sorts of stuff. So that is the, do you have any questions before we keep moving forward?

**MAYA:** No.

**MODERATOR 1:** Okay. Cool. So let me get you involved a little, Maya. So the only question you really missed was, what are some things over the years, and since you’re in eighth grade, you have a little more experience. What are some things you’ve liked or not liked about PE at WRV?

**MAYA:** I don’t like the running the mile every Friday in PE. What I do like is like the most of the activities that we do in PE like the games and stuff.

**MODERATOR 1:** Okay. That makes sense. I imagine games are more fun than the mile. How, can you tell me, expand on the mile a little bit. I’m just curious. Why, what about it don’t you like? Which, what aspect?

**MAYA:** Like how it like, I don’t know, like how it’s like so like much laps around the gym. Knowing that gym is kind of like huge and like I don’t running because it like messes with my like asthma and stuff, so that’s another reason.

**MODERATOR 1:** Okay. Yeah. It totally makes sense. Thanks. Okay. So that’s PE. That’s some stuff you guys like. Leah, so, told me, yeah, so some sports you liked, some equipment you didn’t like. Is there anything else on PE that you think could be done better if we were to, like say we brought college students into WRV to lead some programs, what could we do a little better with PE?

**LEAH:** So I don’t know, actually. I think that’s all I have.

**MODERATOR 1:** Okay. Awesome. Anything else on PE, anybody? All right. Cool. So let’s talk sports. So we heard from Leah, Taylor, Riley. Maya, have you played any sports in the past, or do you play any sports now or not really?

**MAYA:** Yeah. I played sports.

**MODERATOR 1:** Cool. Which ones?

**MAYA:** I played volleyball, basketball, and that’s it.

**MODERATOR 1:** Volleyball, basketball. Okay, perfect. So let’s talk about that a little bit with all of you. So you told us about PE. What are some things that you did not like about your sport experience? So it really, just to your specific sports, and you all played different ones. And how about either, Taylor, would you be willing to go first for some things you didn’t like about softball?

**TAYLOR:** So I’d say, so I’m really bad at batting. And it’s just, you know, I practice a whole lot and, you know, nothing really about batting really helped. And of course, you know, I practice at home or I practice at softball and maybe throwing. Just . . .

**WOMAN:** [Inaudible, 00**:**10**:**48]

**MODERATOR 1:** Yeah. That totally makes sense, Taylor. Thanks for sharing that. How about someone else? What are some things you did not like about your sport experience, either last year or the last couple years?

**LEAH:** I, the fields are not very taken care of. They all have like tall grass or the, there were vines on the fences and stuff like that.

**MODERATOR 1:** Okay. That makes sense. Thanks, Leah. Riley or Maya, and hello, Casey.

**WOMAN:** Say you’re here too.

**MORGAN:** I’m Morgan. Hi.

**CASEY:** I’m Casey.

**MODERATOR 1:** Cool. Morgan. Oh, yeah, oh, awesome. Both of you. Morgan and Casey. Great news. So thanks for coming. Let me catch you up real fast. So Moderator 2 and I are here leading a program called Hoosier Sport, and we’re just having an awesome chat with these guys about sports and physical activity and PE class. And we’re going to going to talk a little bit about some like life skills and leadership and that kind of stuff, because we’re trying to bring our program, Hoosier Sport, to WRV. It’s going to start this fall at school. It’s going to start with sixth graders, but in the future, we’re looking to expand to other groups.

And then we’re also recording the session today, because we’re going to get it transcribed, which means we’re just, we’re going to get the words typed out so that we don’t lose the awesome content you guys are saying. We’re going to de-identify everything, so like we’re going to remove all the names and any personally identifiable information and stuff.

And we’re going to use it to try to find themes, or like common thing, common problems, today’s session, because today’s session is mostly about like problems or challenges or issues. And then, as we move through these five sessions, so today is session one, we’re going to move towards like creating cool ideas for what the program could look like.

After each session, everyone will get paid $40 each session, so either an Amazon eGift card or a VISA physical gift card. Before moving on, two questions for Morgan and Casey. First of all, do you have any questions about stuff, and second, would you prefer Amazon eGift cards or VISA physical gift cards that I would need to, and I’ll distribute through the school.

**GIRL:** Amazon gift cards.

**MODERATOR 1:** Did you say Amazon?

**GIRL:** Yes.

**MODERATOR 1:** Awesome. Okay. And did we get that for Maya already, Moderator 2? Yeah. Okay. Cool.

**MODERATOR 2:** Wait. Maya, did you say Amazon?

**MAYA:** Yeah.

**MODERATOR 2:** All right. Cool.

**MODERATOR 1:** Yeah. Cool. Amazon is a little easier, and then we can get it to you right away. Moderator 2 will send it to you tomorrow. So Morgan and Casey, any questions about anything before we move on?

**MORGAN:** No.

**CASEY:** No.

**MODERATOR 1:** Okay. Cool. And you’re both there, right?

**MORGAN:** Yes.

**CASEY:** Yes.

**MODERATOR 1:** Okay. Great. That’s awesome. Good to have you. So the only question you missed, we’ll start with you two . . . question . . . was about physical, PE class. So Leah, Taylor, Maya, and Riley were all sharing some things they did not like about PE. Would you two be willing to go, one after the other, and tell us some things you don’t like about PE class and how it’s run at WRV?

**MORGAN:** We just transferred to WRV this year.

**MODERATOR 1:** Okay. So how about PE in general, some things you don’t like about PE?

**MORGAN:** My old school, it was very pick and choose, where like it was very gender thing. Like he, our old PE teacher, he had very, he had lots of favorites, and it, like he would put most of the boys in like with most of the girls in the same, one team, like girls versus boys a lot more.

**MODERATOR 1:** Okay. Thanks for sharing that. And then how about, I don’t know if that was Morgan or Casey, but how about . . .

**CASEY:** It was Morgan talking.

**MODERATOR 1:** Okay. Cool. How about you, Casey?

**CASEY:** He’d put most of the boys on the same team and then the girls on the other team. He’d put like the stronger players on one team and then the weaker ones on another.

**MODERATOR 1:** Okay. Thanks for sharing that as well. And then how about some things you like about PE in general, like some things that have gone well in the past?

**CASEY:** I like PE class, because I don’t like sitting in class the whole time, because I get very bored. And I have, I’m really hyper, so I like PE class [inaudible, 00**:**16**:**06].

**MODERATOR 1:** Yeah. Great point. And how about your sister?

**MORGAN:** So I don’t have to sit in class in all day, so I can get out and run around for a little bit.

**MODERATOR 1:** Yeah. It’s nice not to have to sit all day and get away from screens sometimes. Very cool. And also, the other thing I should tell you too, just because you missed it at the beginning, was that there’s no right or wrong answer with this stuff. Like this isn’t a test or anything. We’re just looking for kids’ opinions on programming that will help us create a really cool program with you over the next bunch of weeks.

So then, how about Morgan and Casey, again, can you tell me a little bit about some stuff you either, that you did not like about, I guess I should first clarify. Did you two play sports?

**MORGAN:** Yes.

**CASEY:** Yes.

**MODERATOR 1:** Cool. Which ones?

**CASEY:** I ran cross-country, do soccer, basketball. I did cheer and I do travel softball and school softball.

**MODERATOR 1:** Okay. Cool. So both of you, could you tell me a little bit about some things you did not like about your sport experience, either last year or in the last couple years? It can be really specific or really general, just some things you didn’t like about your sport experience.

**MORGAN:** Me and my sister, opposites in general, so I’m more athletic, and that’s who I am. Oh, yeah, it’s Morgan speaking too. I’m more athletic than Casey, in general speaking. I do more sports. So my sports experiences have been great in general. Casey?

**CASEY:** My sports experiences are great.

**MORGAN:** Oh really?

**CASEY:** Yeah. I like my sports experiences.

**MODERATOR 1:** Okay. Thank you. Thanks for sharing that. And then how about, next question is around your PE, either PE teachers, teachers in general, or also your sport coaches. I’m looking for some things you have not liked about certain teachers. You don’t need to give me names or anything at all. Just in general, what are some things that you don’t like that teachers do? Because as we moved forward with this program in the future, we want to try to like train our students and coaches to do things that kids like. So, Maya, if you don’t mind going first, what are some things that you have not liked about either teachers, PE teachers, or coaches?

**MAYA:** How they like, I don’t know, put the girls to the side and like pick the boys most of the time.

**MODERATOR 1:** Okay. And how about anyone else? What are some things that you have not liked that coaches or teachers have done in the past?

**GIRL:** [Inaudible, 00**:**19**:**42] picks favorites [inaudible, 00**:**19**:**47]. I already answered that.

**MODERATOR 1:** Picking favorites. That makes sense. So that’s a common one. How about, yeah, Taylor, you look like you were wanting to say something.

**TAYLOR:** Levi(?) was, so since me and Leah were in the kind of, the same class, I don’t know if she had any problems, but Levi, our PE coach, he was like always definitely picking favorites. He always wanted to do the same stuff over and over.

**MODERATOR 1:** Great point. So, yeah, variation. Doing the same thing, that could get boring, right?

**TAYLOR:** Mm-hmm.

**MODERATOR 1:** Okay. Moderator 2, how about you? Any question in mind you’d like to pivot to?

**MODERATOR 2:** Going off Moderator 1, you said that you do the same things over and over. What type of things would you like to see more often?

**TAYLOR:** Well, I’d like, I mean, I like playing basketball. I do like playing dodgeball, soccer, maybe going outside and playing softball or baseball.

**MODERATOR 1:** Thanks, Taylor. Yeah, Leah.

**LEAH:** So one thing I did not like about the gym is, this is for the whole school also, there is roaches all over the place. So I just, that’s one thing I don’t like about it.

**TAYLOR:** I agree.

**MODERATOR 1:** Yeah. That’s no good. And, yeah, that’s no fun for anybody. Okay. How about, can you all tell me a little bit more about after school, what you, what, if you, I don’t know, has anyone here participated in the afterschool programming before at WRV or at any school, if you just transferred to WRV?

**TAYLOR:** Sometimes . . .

**GIRL:** I have.

**MODERATOR 1:** Okay. Cool. So we got a bunch of answers. So let’s get at least you three, and then we’ll see about Riley, Casey, and Morgan. So whoever, Maya or Leah, if you want to go first, and then we’ll come to you, Taylor, just about your experience with afterschool programs, wherever you want to go with that.

**MAYA:** I’ve had an afterschool program before for volleyball and basketball. For volleyball, when I normally stay after school, it like, we get there, and then the coach is never there until like maybe like 10, 15 minutes after we get there, because like they like expect us to go there and set everything up before the coach gets there. And then, I mean, I understand why, but like once we get started, like they like do let us like stop and get a drink or something except for when you have like really bad breathing problems.

And then at the end of the program, it like takes forever to be able to like go outside and wait for your parents. But like when your parents, like when you go outside, it takes forever for your parents to get there, and you got to sit there and wait forever. I mean, like it takes a long time. So like I kind of like doing afterschool programs, but at the same time, I don’t because of the time it takes.

So like for my basketball, I usually do, I went to Edgewood, and for my basketball at Edgewood, I’d go there, and then like hardly anybody was there. And then like hardly anybody would tell you where you’re supposed to go or what you’re supposed to do. They just like kind of expect you to do it as soon as you get there. And like, me, on the other hand, I don’t know what to do or anything, so they have to like tell me what to do, but they don’t do that. So that was kind of aggravating.

**MODERATOR 1:** Yeah. Really great points, Maya. You hit on a whole bunch of important afterschool programming stuff. Thanks for sharing that. Leah, how about you?

**LEAH:** So I went to the afterschool program in the elementary before. And so they had Legos, but there was only like only a little bit of them. So me, I’m personally a fan of Legos . . .

**MODERATOR 1:** Oh, nice.

**LEAH:** Fan of Legos. So I would like, if they had different sets, and then you can pick one, build it, and then when you’re done, take it apart, put it back in the box. So I just like Legos that much, so I was thinking of that.

**MODERATOR 1:** That makes sense, Leah. If you remember, could you tell me a little bit about, actually, wait. We’ll come back to that one. Let’s stay on this one for now. Taylor, you had said you had some info to share about afterschool programs.

**TAYLOR:** Yes. So I stayed after for, after school for softball, and I didn’t know which room to go in. They would always say, oh, go to the gym, you know, oh, go to this room, you know, and the teachers are like not even there. So, you know, what, where are you supposed to do, you know? You’re like, are you supposed to wait, you know, or like what room are you supposed to be in?

**MODERATOR 1:** Yeah. Really great points, Taylor.

**TAYLOR:** They had like last year, I had to go to at least three or four different rooms, and I like didn’t know where to go at all. So I kept on asking teachers and teachers, and they kept on saying, oh, just go to the gym. And our coach always said, oh, you know, go to this room or that room.

**MODERATOR 1:** Yeah. So that would be hard, if it’s disorganized like that. Leah.

**LEAH:** So for me, in softball, I would stay after school, so, hold on, I forgot what I going to say. Oh wait, yeah, okay. So our head coach was always sick. So we had to have a [inaudible, 00**:**27**:**30] coach coach us like most of the season, because our actual coach was sick like half of the season, and she wasn’t there very long. She was only there for a couple of days.

**MODERATOR 1:** Thanks, Leah. Great info. Riley, Casey, Morgan, anything you want to add about some things you did not like about afterschool programming, if you’ve participated in some?

**MORGAN:** This is Morgan. I did most like the sports. The afterschool things, they’re fine. It’s just most of our coaches would show up late. And like our head coach wouldn’t [inaudible, 00**:**28**:**17] for like basketball and things. [Inaudible, 00**:**28**:**26] like in general. Casey?

**CASEY:** Our coach was like never there, wasn’t always there on time. We’d be, we’d have to [inaudible, 00**:**28**:**39] school usually. That’s all.

**MODERATOR 1:** Good. Thank you. Riley, anything you wanted to add about afterschool programs?

**RILEY:** No.

**MODERATOR 1:** Okay. So then the, everybody else, Casey, like a time kind of question with those afterschool programs. I’m wondering if, one, if you got like a snack at all between going to school then going to the program? And I’m also wondering what you think is like the ideal time. Like if you think they should be shorter or longer, or finish at 4**:**00 or 5**:**00, so those two things. Experience with getting a snack after school and before the afterschool programming and then also time. Could one of you expand on that a little bit for me?

**GIRL:** [Inaudible, 00**:**29**:**35]

**MODERATOR 1:** Leah.

**LEAH:** So we would never actually get a snack, but when we were waiting to go to the gym or to ride the bus. But one time I stayed after school, my teacher went to the Dollar General and actually got us snacks, which was very nice of her. I wish they would have gave you snacks. And, well, I think they should have it earlier like they should have it at, our practice at 3**:**30 and end at 4**:**30, so we can still stay at school and it won’t be that long, because one time I had to wait until 5**:**00, when it said on my mom’s phone 4**:**00. So that’s all I got.

**MODERATOR 1:** Thanks, Leah. Taylor or Maya, you look like you want to add something.

**MAYA:** The timeline is like for like when I get to like, after school ends, my like session things normally start around like, I don’t know, like 3**:**15. Then when, I can always sit up in the office or in the cafeteria. And then for my volleyball practices, and I have to stay after school for them, I sit in the cafeteria and the cafeteria would like have snacks laying out for us.

Like and then like it takes forever for, after we get done with our snacks, we would have nothing to do for a while after eating, because the bus hasn’t showed up, and then we’d go outside, and we get on the bus. We get to the practice, we have a chance to grab water bottles and stuff and get a drink. And then we start our game, and then at the end, we get a snack . . . and a drink. And then on top of everything, it’s normally like, I don’t know, like 5**:**30 or something. And then by the time I get home, it’s almost 7**:**00, and I won’t have like that much time to do anything.

**MODERATOR 1:** Yeah. That’s late. That would be tough. Thanks for sharing that, Maya. Taylor, did you want to add something?

**TAYLOR:** Yeah. So like whenever we would have a snack, we would always just have milk over and over and over again, you know. And we couldn’t like have like no juice or, and the same whenever we have lunch. We always had to have juice or, because whenever, because I don’t, myself, I don’t really like milk. So at lunch, I, whenever I can, I try to get water, because I’m not really a milk fan.

**MODERATOR 1:** Yeah. And you’ve having to have a lot of milk. That’s no fun. Leah. Thanks, Taylor.

**LEAH:** Yeah. Same thing. I don’t like milk either. They should have a choice of juice and stuff like that, because now you have to buy it. You have to buy the juice, the bottled water. You can get free water, but it’s from the sink. And we’re all mad and everything because you have to pay for juice, and usually you don’t want to pay for like $5.00 for a little thing of juice.

**MODERATOR 1:** Yeah. Good point. Great point, Leah. Casey, Morgan, Riley, anything you want to add on that one?

**RILEY:** No.

**MODERATOR 1:** Casey, Morgan?

**MORGAN:** This is Morgan. [Inaudible, 00**:**33**:**42] our practices like for softball, it would start at 5**:**00. So we’d have like two hours between school. Like those Fridays, we’d have three. [Inaudible, 00**:**33**:**52] at Bloomfield(?), before I transferred, ended at 2**:**00 every Friday. But like snacks, you would have to go get snacks by yourself with your parents or something like that. Basketball, we had to get [inaudible, 00**:**33**:**52] snacks at any close gas station or something. I usually bring stuff and have extra stuff in my lunch box for like basketball and cross-country. [Inaudible, 00**:**34**:**29] something in my lunch box for after [inaudible, 00**:**34**:**34].

**MODERATOR 1:** Thank you. Let’s see. So let’s pivot a little. We have just a few minutes left, and then we’ll get you guys all out of here. So I have a question about, Moderator 2, do you think we should go into screen time or social stuff?

**MODERATOR 2:** Let’s talk about screen time.

**MODERATOR 1:** Okay. Screen time. So at school, and these two might go hand-in-hand. We’re trying to learn a little bit more about screen, how much time you guys spend on screens and also like sitting and non-active time. So I don’t know if most of your day at school you spend it sitting or most of the time you’re sitting and on a screen or not. But can you tell me about some challenges you have at school or even outside of school around screen times and like sitting and not being active, that kind of stuff? Taylor . . .

**TAYLOR:** We always, so when I was at . . . in fifth grade, they would have us sit down, and they would like we wouldn’t do everything on paper. And a couple of our classmates struggled with that, so I think we could possibly maybe do a couple things like not on electronics and maybe do some on paper.

**MODERATOR 1:** Thanks, Jasmine. Yeah. Great ideas.

**TAYLOR:** I’m not Jasmine. I’m Taylor.

**MODERATOR 1:** Oh, sorry. Sorry, Taylor. Sorry. I see your name there, Katelyn, but it’s Taylor. Thank you. Leah.

**LEAH:** Okay. Yeah. So when we go to school, I think we should take breaks and go outside sometimes. And maybe, if you’re working on your iPad a lot, maybe you could get a break and play games for a little bit. I don’t know, maybe for like a couple minutes, because when I get home, and we don’t get to play that much, you know, on our tablets and everything. I usually am on my phone for a couple hours.

**MODERATOR 1:** Thanks, Leah. Great info. Who else? Maya, Riley, Morgan, Casey, could you tell me a little bit about screen times, sitting time, school, home, that kind of stuff?

**GIRL:** [Inaudible, 00**:**37**:**30] on our computers, but then we’d learn math on computers. So we had some [inaudible, 00**:**37**:**34] computers. [Inaudible, 00**:**37**:**47] so it was like a good hour of each [inaudible, 00**:**37**:**55].

**MODERATOR 1:** Okay. And Maya, would you be willing to share a little bit about screen time, activity time, what that looks like for you? All right. So let’s see. Moderator 2, any last questions before we wrap up?

**MODERATOR 2:** I think that’s about it. Riley, do you want to say anything about it?

**RILEY:** Yeah.

**MODERATOR 1:** Yeah. Let’s hear it.

**RILEY:** Sometimes when I get home, my neck is really, really sore from the tablets in class.

**MODERATOR 1:** Yeah. That makes sense. Thanks for sharing that. Lots of head down position. Maya, did you have anything to add on that, on screen time, sitting time?

**MAYA:** Yeah. I was trying to, but like my phone was on mute. But I was, I don’t really spend much time on electronics, because they like messes with your head and stuff. But like kindergarten to like third grade, I’d like take naps all the time in class. And then from there to like maybe like fifth grade, we’d have to do everything on like paper, and I don’t like doing much of that.

But like sixth and seventh grade, I was in Edgewood, and they’d have like regular [inaudible, 00**:**39**:**29] break type things where you can have time to go outside and take a walk around the school and stuff. And I’d get [inaudible, 00**:**39**:**29] or something and I’d like go outside, and I’d like hang out underneath a tree or something. And during seventh grade, they don’t really have you on a tablet much. They like, they mainly have you work on paper.

And like when it comes to like reading books or something, I’m not like a fan of that, because like I get really bad headaches after reading, because my eyes don’t like focus on my book. It skips lines, and that’s why I am supposed to be wearing glasses, but I’m not. So, yeah, I don’t spend much time on my phone either at home, because I’m normally outside playing or hanging out with friends.

**MODERATOR 1:** Awesome. Thanks, Maya. Anyone else have anything to share before we wrap up?

**MORGAN:** This is Morgan. So [inaudible, 00**:**39**:**35] the seventh grade, [inaudible, 00**:**40**:**37] actual work, so like math class, most of our schoolwork was on like a Chromebook and stuff. So we didn’t have a lot of time [inaudible, 00**:**40**:**49] because most of our teachers [inaudible, 00**:**40**:**55] didn’t have to like sit there and [inaudible, 00**:**40**:**59] of paper, but like not a lot of other teachers did. So they just like [inaudible, 00**:**40**:**59] to us. And they would help us as much as [inaudible, 00**:**41**:**13] or like [inaudible, 00**:**41**:**19] at Bloomfield.

**MODERATOR 1:** Thank you. All right. So awesome job, everybody. I really appreciate that. You all nailed it. So this session, this is number one out of five. This one was a little more like we were trying to talk about, and for anyone that came in late, we were trying to talk more about like challenges . . .

Indiana University Sport Management

Hoosier Sport

Adult Design Session 1

**MODERATOR 1:** . . . in this study, at time you guys can, you can drop out. You can, you don’t have to answer any question you’re not comfortable answering. We’re just trying to create a really collaborative space. This is also very informal in the sense that it’s not like a test or something. Just trying to get valuable information from you all. Because the last thing we want to do is come into the school and think that we know everything and think we know what the kids need and want and families want and need and what the resources are. We just, we have a whole lot of learning to do.

So that’s why these sessions are going to be great. We’re also doing a small group of five to seven kids as well that will go in parallel to this. So on Thursday evening, Moderator 3 and Moderator 2 and I will be meeting with that small group of kids which is going to be really cool to hear what they say. It will probably take some time to get them to open up a little. But, yeah, we’re going to try to do the same thing with them, and it will be cool to see how your vision and kind of perceived challenges and ideas kind of flow with theirs as well.

So, before we dive into some questions, some ground rules just for, we’re trying to have a discussion and not a debate. I want, everyone’s encouraged to participate, but you don’t have to participate in any given question like I said. We don’t want one or two people to dominate the discussion, but at the same time, don’t be shy to say what you have to say because I’m sure you all have pretty good stories about the resources and physical activity stuff going on in other programming at this school.

And then one thing I try to really upgrade with it all, this is kind of flowing from the seek first to understand not to be understood, so trying to understand your perspectives with the programming at WRV. Also, we really want this to be a long-term collaboration, so like I mentioned at the very beginning, I’m just starting at IU, and I’m hoping that we can make this WRV connection go for a very long time.

It’s going to start small with sixth-graders, but a little bit about the vision is that we’re trying to use the power of sports. So sport is like the hook to get kids interested in programming, but then have broader developmental goals like maybe leadership or nutrition or physical activity, but framing it kind of around getting kids interested in sports rather than like an exercise intervention because no one wants to do that.

Mutually beneficial as well. So as you think, as you envision this program growing in the future, we’re starting something called a Service Learning course at IU. So the idea is for college students to come to WRV and deliver this programming in the future. So in this coming fall, it will be our small research team like me and Moderator 3 and Moderator 2 and a few others, undergrads, graduate students that will come to the school. And there’s some like constraints with the program this fall, and then we’re going to retest it in the spring, but the idea is to grow.

So we want to go to other grades and have more programming like, and there’s a really cool program called Husky Sport. If you guys leave here today and look up one thing, that’s probably the thing to look up. It’s Husky Sport at UConn at the University of Connecticut. They’re a really well integrated program with the community, so that’s kind of what we’re framing all this after.

I think those are the main points. Anything else, Moderator 2 or Moderator 3, before we dive in? Cool. Okay. So your turn. I want to be quiet and do some listening. So would someone be willing to start with, when you think about WRV Middle School, what do you think are some major concerns and issues related to children’s health and physical activity?

And I should tell you that today’s session, where we’re focusing totally on, the theme is problem identification, so we’re trying to figure out what the challenges are with physical activity and sports and programming at the school. Next session we’re going to get into some like creative ideas and brainstorming and stuff, but today is all about problem identification. So we really want to learn like what’s been tried in the past, what’s worked, what’s not worked. So that question, when thinking about WRV, what do you think are major concerns and issues related to children’s health and physical activity? Who would like to go first?

**ANITA:** All right. I’ll go first.

**MODERATOR 1:** Anita, perfect.

**ANITA:** I mean, I’m not wanting to step on any toes or hurt anybody’s feelings, but I do think sometimes parents with sports, they think of coaches with favorites or kids with money, things like that. It’s not always like that there, but I know that sometimes that’s the first thing sometimes that parents and people think of.

**MODERATOR 1:** Thanks, Anita. That’s awesome. Yeah, favoritism is big with sports, with any sport and physical activity stuff. Thanks for sharing that. Who else? What other concerns, issues related to children’s health and physical activity? Olivia.

**OLIVIA:** I think part of ours, our, at least as a teacher standpoint, we really don’t have what we would consider a true P.E. teacher. We have an aide that’s in there. And I do think, you know, it’s kind of been a revolving door, and so there’s not really a set curriculum for the kids, and so a lot of kids just sit out during P.E.

And I’ll walk by the gym, and, you know, there’s kids sitting on the bleachers. It’s like they don’t want to be active, or maybe they don’t like the thing that the teacher is working with. That’s just from my perspective, I look at it that maybe we don’t have a curriculum for the kids, and they get bored with it. I don’t, is that what you’re wanting us to talk about? I’m a little bit . . .

**MODERATOR 1:** Yeah. That’s perfect, Olivia. So that is excellent like insider information, which is awesome. So no curriculum, revolving door. Could you tell me a little more about that? Like is it, has, is there, was, did someone retire, or is there not the funding for a P.E. teacher, or was the, or was it just like not a good fit or something?

**OLIVIA:** I honestly can’t remember the last year we’ve had a true P.E. teacher. I think this is Levi’s second year, but I could be wrong. And he’s just an aide who hasn’t had any like education background. He’s great with the kids as far as I can tell, but I honestly can’t tell you the last time that we had a true P.E. teacher.

I think the only true P.E. teacher is was like when it was L&M, Mr. Alifant(?) and Mr. Tarell(?) were the P.E. teachers, and they were teachers that, they also taught like biology and health. The high school has a P.E. teacher, but like the elementary and the middle school don’t have a P.E. teacher.

**MODERATOR 1:** Yeah. That’s sounds like a really good opportunity, definitely a challenge to get anything going without curriculum and without someone consistent. What about, so the aide, is that like a, is that a full-time person that helps with various classes, or is he, he kind of is responsible for all grades P.E., or do you know any more about that?

**OLIVIA:** He floats. Like I know he has P.E. classes and he teaches, last year it was fifth, sixth, seventh, and eighth. And then he would float in with other teachers to help them as needed. Because I know last year he helped me. He’d come in and get papers for, to make copies and stuff. But it, he’s there from, I don’t know his hours to be honest. I know he’s there. He’ll be in today, but I don’t know what time he comes in.

**MODERATOR 1:** Thank you. That’s super helpful. How about Jasmine, Monica, Kim, any other thoughts on major concerns, issues you see outside of not having a dedicated P.E. teacher? Or you could even expand on that if you want to.

**JASMINE:** . . .

**MONICA:** . . . oh, sorry.

**JASMINE:** I know when my daughter played . . .

**MODERATOR 1:** Oh, no, Jasmine, we lost you a little.

**JASMINE:** Did you hear me?

**MODERATOR 1:** No. We heard you start and then it went away. But you’re back.

**JASMINE:** Am I back?

**MODERATOR 1:** Yep. You’re back.

**JASMINE:** Sorry. I’m sorry. I know like she didn’t get to play all the innings. We had some issues there. She didn’t get to really interact with a lot of the kids. Of course, she’s been at WRV for three years now. But there was not much interaction with the coaches, practices. I kind of felt like nothing for her to like, we would have to practice with her at home, which was fine because, you know, that’s what parents do, but there was no extra effort I guess you would say. I don’t mean that bad. I don’t. There was just no extra umph, I guess. I don’t know. We had it different when I was in sports. We had to play, and we had to practice and everything.

**MODERATOR 1:** That makes sense, Jasmine. And so that would be challenging. Does that seem like, did it seem like it was your daughter that like was being singled out in that way at all, or did you feel like that was an experience a lot of the kids were having?

**JASMINE:** No. There was, I think there was a lot more that just her that just didn’t get the help or the support or whatever from the coach or whatever they needed to try a little bit harder on or practice on.

**MODERATOR 1:** Yeah. That makes sense. Yeah. Some coach training opportunities might be there. Then, Monica, hi, dear, you wanted to add something.

**MONICA:** I was just going to say like my daughter is going into fifth grade, which before this year, would have gone over to the junior high, and they, they’re keeping her at the elementary this next year. But their P.E. is, they’re specials, so they don’t even do it every day. And depending on what, who they have in sometimes determines how much activity they have in those specials.

And so it’s been, and this isn’t just her, this is, you know, the whole class. And so she was really looking forward to going over to the junior high in fifth grade so that she could play basketball, but now she’s staying back and she’s not sure where that’s going to go. But at the same time, P.E. for her is not something that is a regular thing.

**MODERATOR 1:** Yeah. Thanks for sharing that, Monica. You’re all hitting on like such good topics, the resources or the teacher, the frequency of P.E., the coach training, or what’s actually happening at practice or in P.E. That’s great stuff. Kim or Anita or anyone, anything else on those concerns, issues? Even with their health more broadly like I don’t know if nutritionally, or, one of the school administrators mentioned being interested in leadership-like programming, so anything more broadly with their, with concerns or issues related to their health or their activity levels?

**KIM:** Since this is going to be my kid’s first year at WRV, I don’t have too much on that. I think one thing that’s going to make it difficult is the way the schools are spread out and transportation between the two. I’ve been told it’s really great, but I know that could affect some of the kids’ ability to do sports is transportation because we are so rural.

**MODERATOR 1:** Yeah. Great point, Kim. Could anyone tell me more about that, the transportation, in general, like what you would anticipate for, so say we tried the afterschool programming or something like that, what the transportation resources currently are or what the, or more about that issue? Like would some kids automatically not be able to do it because of transportation issues, or, I don’t know if there’s like a van or bussing with that, like could anyone share more about transportation?

**ANITA:** I believe there’s only the shuttles for the school. Like after school, there’s no shuttles for any of that I don’t believe, unless it’s like the, they stay after for an afterschool program. I know they do shuttle them back to the high school. But for most kids, unless their parents are willing to do the extras, they probably couldn’t get back and forth.

**OLIVIA:** Well, I know like we do have an afterschool program for the kids. There’s a little bus that does shuttle them to the high school, but that’s as far as it goes. They don’t shuttle them to their houses. And a lot of times, I think that limits a lot of parents. Because, you know, in society, we have parents that are working all the time, and so it’s hard on those parents, and, you know, it just takes away from the kiddos.

**MODERATOR 1:** That definitely makes sense. Thanks for sharing that. Could I probe a little the, so coach training and sport participation? So P.E. teacher, we talked about a little bit, and that definitely sounds challenging. Could you tell me any more about with sports, like how many kids are playing sports? What do you think of the quality of the sport experience at the school?

This, just to be like clear, we’re not trying to start like a new afterschool sports program or something, but we’re totally open to ideas. So anything more on coach training or experience or how many kids are taking advantage of getting involved in sports at the school?

**ANITA:** I think the first, from not just the school, but like every school, the first thing, sometimes like the coaches are a father or something like that. So I don’t want to sound bad, but sometimes when it’s daddy or mommy ball, that becomes a problem. So, because I feel like when it’s parents, I feel like sometimes the other parents feel like they can bully that parent to play their kid or something like that.

And, I mean, the softball program for the middle school is an example. The coach this year, he tried to do a great job. He really did. Sonny(?) put a lot into it and made it a little better this year. But at the end of the season, it became mommy-daddy ball. Parents were griping about kids not playing. You know, like we did travel ball on top of that, so we were doing ball seven days a week. So they would, like we had already worked it out with the coach from the beginning that we wouldn’t be at certain practices because we were at other practices, and he was okay with that because she was practicing ball.

But then some of the parents would get upset because, well, their kid’s not at practice, you know, but it wasn’t like they weren’t practicing. She really was practicing somewhere else, you know. So I just think sometimes parents have a, it’s not really the coach that’s the problem, it’s the parents getting involved is the problem.

**MODERATOR 1:** That makes sense. Thanks, Anita. How about thinking a little outside of physical activity in terms of physical activity and sport, but what about concerns during the school day with either nutrition or their activity time like how much they’re sitting, their screentime, any concerns there that you think, that you’d like to see a program come into the school?

Because the school, the principal and the school administrators we’ve talked to so far have been totally open to ideas around like policies or kind of like environment systems changes, not necessarily just individual behaviors, but anything you else, anything else you see that’s a big concern to you outside of just sport-related physical activity?

**OLIVIA:** So physical activity, I’ll be really honest, our schedules are tight. And when I say tight, I mean like we have, we each have a block to teach our certain topic, whatever it may be. We are a one-on-one school, and so screentime is a lot. I’m not going to deny it. A lot of times I’ll just say, shut off your iPads. We’re not, shut your eyes, I mean, it’s just too much for these kids.

I’ve kind of pushed trying to have what I would call a recess at the end of the day just to decompress. And we’ve worked that in, and it’s like it literally it’s just 20 minutes. But sometimes the kids just need to go outside. But if you go outside to our playground, it, no offense, it sucks. It’s terrible. They have nothing for those kids to do . . . are broken. It’s just bad.

**MODERATOR 1:** Yeah. That makes sense, Olivia. Thank you for sharing that. So you’ve started some recess. Could you tell me a little more about that? Is it daily? Is it all grades? Was it super challenging to get buy-in or a little bit about that process?

**OLIVIA:** We can’t call it recess.

**MODERATOR 1:** Okay.

**OLIVIA:** Can’t call it recess. And it’s only sixth grade. When fifth grade was there, they did have a recess, and we’ll call it recess for right now, but on paper I have to call it life skills. So we do life skills outside, and I do do life skills. We talk about how we should behave that kind of stuff, and then we play. But fifth grade did it every day. I can only do it 3 days a week because we do what’s called careers two days a week for that 20-minute slot.

But, you know, why couldn’t we implement, which we do have a careers program, but why can’t we possibly in that 20 minutes or whatever, for seventh and eighth grade even, because they have a what’s called homeroom, why couldn’t we implement something for the kids health-wise in that 20-minute slot or whatever so it’s not screentime all the time? But I’m also old, and so I don’t really care for the iPads.

**MODERATOR 1:** Me neither. Amen. So how about with life skills and careers, you mentioned, does anyone, Olivia, or anybody else have any thoughts on what some of those biggest concerns to you are or biggest things? The flipside of that would be biggest things you’d like to see kids in sixth grade working on, what are those topics or issues, like is it, yeah, I’ll leave that open before I throw ideas out there.

**MONICA:** I think life skills should be adding social skills because when we do so much screentime, and it is phone time and iPad time and everything, they don’t know how to communicate with each other. So then if it’s typed out on a screen, then they don’t know how to do anything, which means then they, and I don’t want to say they get backwards, but the shyness and the not being able to communicate in person becomes a problem.

And I think what Olivia does with the life skills, as she says it is, is great. But I don’t know that Olivia is not one of the only ones that does it. So I think it’s fantastic, and I think it should be more across the board, and it should be implemented into the program completely where everybody has to do it.

**MODERATOR 1:** Great ideas, Monica. Thanks for sharing that. Jasmine, Anita, Kim, anything on life skills, careers, social skills, and activity during the day, any of that kind of stuff, screentime?

**KIM:** Well, I know the kids have tremendous amounts of screentime. Even with as much, like my kids do travel sports as well, and as much as they are busy, the amount of screentime is unfathomable to me at this point. When I see their log at the end of the day or the end of the week of how much they’ve done, especially over summer, because they do, they have the best of friends online, and then now it’s trying to convert that to a physical friend in person.

Which, since we’re transfers to WRV, my daughter is friends with half the school it seems like already, but it’s because of the iPads and the text messaging and whatever the Snapchat, all the stuff they use. And now to try to turn that into a physical friendship, relationship as well, and how to deal with that in person, of if they’re not getting along or something there, they can just turn the screen off. Oh, I lost connection. You can’t do that when you’re in person.

So I, to me its kids need to learn how to deal with things in person and that’s, to me, that’s something that they don’t do anymore. Like I guess I’m old too, and you got in a fight, it may have been a fight, but ten minutes later, you all made up. But now everything is carried over to the next thing, whether it’s sports, academics, or anything, it’s all out there online for everybody to see all the time.

**MODERATOR 1:** Thanks, Kim. That, yeah, that’s challenging taking a big friend group that you have like established texting relationships with and turning that into something. Moderator 2?

**MODERATOR 2:** Could I add to that?

**MODERATOR 1:** Mm-hmm.

**MODERATOR 2:** Would all of you, thinking about the kids, tell me if you think that, do most kids think about, like do they acknowledge, can they acknowledge or talk about, okay, I’m on the screen a lot, okay, I’m not very active, or are they ignoring those behaviors? I’m just curious about how in tune they are with their own behaviors. I think, I don’t know if you speak to that. But what I’m saying is, oh, I guess I’m on the screen a lot, you know. Are they acknowledging the time they’re spending doing these things? And if not, I don’t know if you can speak to that or talk a little bit more about that, I’m just very curious.

**MONICA:** Well, I’ll start. My ten-year-old, I don’t let on the screen during the evening or whatever because she has it at school all the time. And she can tell me more about Google than I can. So let’s just clarify that. My two older boys, no, they cannot recognize that their head is buried in the phone and on their iPad and then their game system all the time. Now they are very active. They both play sports. But when it’s downtime, they are, they’re completely screentime. And I have to be creative and find ways to get them out and moving without them knowing that I’m doing it.

**MODERATOR 2:** Thank you, Monica.

**ANITA:** I agree with that because they’re not knowledgeable about how much time they’re spending on their screens. And with school going back, yeah, they’re going to be on their iPad a lot, I mean, and the phone like 100%. You know, she’s definitely not knowledgeable how much time that is.

Like when we were growing up, like Olivia and myself, we’re same year of school, I mean, we had classes like home economics, like things like that. Like they have took all of that mostly out of the schools now, and things like that kept us, you know, you learned a life skill, like she said, and that’s what they need more of to be honest.

Because at least they’re, they all have to participate in groups because the teacher usually puts you in groups. Guess what? There you are learning those social skills with other kids that you might not have done social things with, you know. So I think that would be more helpful.

**OLIVIA:** Right or wrong in my classroom, I call it, it’s called turn and talk when we do math, and I make them talk to each other. They’re, it’s not all on their iPads, and they have to do a problem and figure out together how to solve it. I do think the kids today lack social skills. You know, we play Bingo. You know, find somebody that did this on your board, you know. You know, you have to talk to them about it.

The kids just, and technology is great for a certain aspect, but it’s been a downfall for students because just like, I think it was Monica, I don’t remember which mama it was, that, you know, you can just turn off your phone, you know. They don’t understand how to, conflict resolutions, they don’t know how to do that when they get upset. They don’t know how to talk it through.

You know, when we go back to life skills, you know, I had the kids where they had to make a video of themselves making something at home because we don’t have, you know, the stove and everything here. And they talked about it. They did a presentation. They hated it to start with, but they got out of their bubble. I don’t know if this is a program that will help them with it, but I think anything we can come up with, it’s going to benefit them, absolutely.

**MODERATOR 2:** Thank you. As a follow-up to that, would it make sense to create programming around educating them about, you know, this is how many steps you should get a day? This is like, you know, just kind of talking to them about how much time, monitoring your screentime. This week we’re going to monitor how long we’re on the screens. Would they benefit from learning about some of these things and whether or not they act on that, that’s different, but just kind of bring it to surface?

**KIM:** I think it probably would be beneficial because I know not every parent does. Kind of that’s the joke, that’s the babysitter, oh, here’s the iPad. You see it when you go to a restaurant that, oh, well there, like kids screaming. Well, their mom pulls out the phone, hands them that, and they’re suddenly watching *Octonauts* on the loudest, you know, volume possible for the whole restaurant to hear with it.

But I think it would be good for kids to see that, that how many steps they take, and most kids, and not most kids, I know a lot of kids have the Apple watches, or they have phones now and that tracks it all, and they can easily see how many that is. So we kind of make it a contest in our house of who walked the most today and they kind of get to go back and forth on that to see who’s on top.

**OLIVIA:** I know a couple years ago, I could be wrong on the years by all means, it seems like our school purchased like those tracking for the kids, and they would, you know, like I don’t remember how they did it. I honestly can’t remember, but I remember them having these little devices, and then they tracked how many steps they took. But that’s been several years ago, and I have no idea where those little gadgets went.

But it’s holding them accountable, I think, is the best thing, you know. Yeah, we’ve got the watches, but some people can’t get those watches. Like when you guys were talking about it, I looked down at my watch how many steps I got. So, I mean, would they do it? I don’t know. Might, I don’t know, I’m going to go turn off the fire thing.

**MODERATOR 1:** Anybody else, anything on that, life skills, awareness? No. All right. Well, let’s move on. So next topic that I think you five are perfectly situated for. So how do you envision where parents being involved in physical activity and the behaviors like this?

So say we were working on like step program or an awareness of screentime or nutrition or that kind of stuff, where, Olivia, you mentioned not sure if we could work on that in this. I think right now we can see that there’s like a blank canvas, this program. I really, really value life skills and things outside of, like the whole reason that I love sports is to, all of the other developmental benefits of it. So we do want this program to be more holistic in that way.

But the thing is like you can’t do everything with one program. So, but with that in mind, what, how do you envision parents being involved in physical activity and this type of programming? What role do you think they should play in this?

**MONICA:** I honestly think that parents should be involved in it, but do it as a willing participant rather than, okay, so we’re going to do this because this is what’s best for you. If we’re setting the example, or the parents are setting the example and getting involved, then the kids would become more involved and want to do more things because they’re seeing the parents setting that example. But not to have the parents be like controlling, undermining, or as one of the other ones said, bullying other people because their kids are better.

And I’m one of the parents that sit in the stand and go, okay, so that’s my kid playing. I love watching him, but I’m not the one that’s trying to push him into being the best on the court. They have to be a sore loser as well as a sore winner, or a good winner, you know what I mean. I’m saying it wrong. But they can’t be, either way, they can’t, they have to be able to teach them that whether they win or lose, they’re supposed to be doing it with humility and respect. There, I’ve said it the right way. I apologize. But I think that the parents should be involved.

**MODERATOR 1:** Thanks, Monica. Who else? Kim?

**KIM:** I think the parents do need to be involved, but it, to make it mandatory, I think you’re going to get of lot of parents, a fair share of them that aren’t going to want to be, they’re forcing me, they make them go to school all day, and then I’ve got to come home and they make me do this. And it’s kind of one of those fine lines. If you make it fun for the kid where the kid wants to do it and tries to get their parents involved with it as well, is a good way to go. Otherwise, that’s the Catch-22 with all of this, I guess, is getting the rest of the parents to get on board to get their kids active.

**OLIVIA:** I think we’re always going to have the, some parents that just don’t want to do it. But if we get these kids excited, and they come home, the parents are going to be excited because their kids are excited. So maybe that, that’s the hook for these parents, you know. They see the joy that these kids have, and they’re just like, I want to feel this with my kid. This is a great opportunity to have bonding with my child.

Of course, I’m, you know, I’ve got a 22-year-old and 26-year-old, so the bonding is far, few, and in between. But when they were little, they would come home, and they’d be excited that they did this or that. And so, you know, they’re telling me all about it. So if we can get them excited, I think it’s going to carry over to the parents.

**MODERATOR 1:** Thank you. Heard some great themes there. Fun and excitement and you got to keep your autonomy, so being careful what’s forced and what’s told to, told that needs to be done. And, yeah, we’re, Moderator 2 and I, one of the things we said most in our house is autonomy and we like celebrate it all the time. So we really want this program to be built around autonomy as, whenever possible. Like give kids choices of which thing they want to do. That kind of stuff. So great advice there.

Anything else on that before we move on, parents, Moderator 3, Moderator 2, anyone else, parent involvement, role for parents, or we’re ready to move on? Good. Okay. So how about, so 7:45, yeah, think we can be done at 8:00 to just after 8:00. We’re making awesome progress. This information is so good. So how about, hmm, key resources or school policies. Let’s talk about key resources.

So who are some of the most important people or most important programs or most important resources of any kind that you’d like to see us integrated with? I was talking with Mitch Hobson the other day, and he mentioned something about, it was, I think it was their school lunch, or afterschool snack provided, something like that, that sounded like someone we definitely needed to connect with. So who do you, who and what resources do you think are like the best things at WRV, or other, yeah, other people that we, you think we should connect with? That was a long question, but I think you get what I’m saying.

**OLIVIA:** I think I’m confused. So are you wanting this people to want us to connect with or things?

**MODERATOR 1:** People, things, or programs, any of the above. Just looking for things that we would be silly not to connect with.

**KIM:** So like the, I know WRV has the free breakfast and lunch for all the students. Are you wanting like the name of the person who runs that or who got that provided to the school?

**MODERATOR 1:** Either.

**KIM:** Because that’s one of the fantastic things that WRV does.

**MODERATOR 1:** Cool. If you had a name, wonderful. If not, just knowing that that program exists is enough because that’s something that we could potentially partner with depending on a whole bunch of logistics. But, yeah, that’s a perfect one. So it sounds like they offer free breakfast and lunch every day to everybody.

**KIM:** I believe so.

**ANITA:** I think Renee Wiggington had a lot to do with that, in getting that started and looking for the, I don’t know if it was a grant or something that the state . . . for that. I think she had a lot to do with that, so that would be the one person, I think.

**MODERATOR 1:** Thanks, Anita. You know if after school, is there anything nutritional that goes on? No.

**OLIVIA:** So there’s days that I do the afterschool program, and literally sometimes they get like, you know, the cheese sticks and juice, and that’s it. It’s bad.

**MODERATOR 1:** Okay. So we’ve got an opportunity there. Nutritionally, how about, could you tell us a little more about what kids typically are bringing to school? What, like if many are, are they just solely relying on the breakfast and lunch there, or if there’s anything else going on? I guess if there’s a free breakfast and lunch program, most kids don’t bring food to school.

**OLIVIA:** So I can only tell you from my sixth graders, most of my kids last year did not eat school lunch. They still brought their lunch. They could go up and get a tray if they wanted to, but most of my kids last year, they brought their lunch. In my classroom, I know I’ve got some parents that I’ve had, kids don’t learn if they’re hungry, so I’ve always opened that up.

Monday through Thursday we have what they call healthy snacks. Whenever they’re hungry, they can eat in class. Now Friday, I’d let them kind of have more like a fun Friday and they bring junk food because, and most of them did, and if they didn’t, I always had snacks for them in class, that, because kids are hungry, and they don’t want to learn if they’re hungry. So the school still allows me to allow the kids to eat and drink in class because I like to eat, so.

**MODERATOR 1:** Me, too. Thanks, Olivia. And before we get towards like ideas and solutions and stuff, similar to how we talked activity and sport and screentime, could you tell me a little bit about what you five perceive are the biggest concerns, struggles, related to nutrition for kids? What, yeah, you’re all on the front lines so you know what some of the potentially most important things that they could improve upon.

**ANITA:** I mean, I think as a parent, like Olivia said, that was one of my daughter’s favorite things about her class is they got to eat in class if they brought the snacks. As a parent, like we have to kind of limit that choice on, because sometimes, I mean, most kids are going to choose the junk, so you have to limit on what you allow them to do.

Like I let her choose one junk thing in the lunch, but the rest is things that I know that, is, I mean, she has long days, afterschool practices, things like that. I make her choose things that are going to keep her going, and she’s not going to drop out, you know, mid‑practice. Because if she was probably to pack and she would probably, you know, choose not always the good things.

**JASMINE:** My daughter hates any kind of vegetable, so I have to like make something with it and then put it on it, like broccoli and cheese. So I’ll just do that. She hates broccoli period. But if it has cheese or ranch or other stuff with it, I know that she will eat the heck out of it. But if you just give her broccoli, she will say, nope, I’m not eating it.

And there’s other fruits and stuff too that she will not eat. But like I put them in different things because I tell her, if you’re not going to have fruits, you have to have a fruit and a vegetable period. No matter what it is. No matter how it’s cooked or anything. You need to have something with it. She don’t like it all the time, but I know she has to have a fruit and a vegetable and not all the junk.

**KIM:** Okay. I’m the bad parent then. My kids don’t go to school normally with fruits and vegetables unless it’s fruit snacks. My oldest gets, she makes her own lunch. She decides what she wants. Usually she’s pretty good with a peanut butter and jelly and I let her take a lot of the other snacks too because it . . . when it comes to the end of the day, if it’s a matter of eating or not eating, I’d rather have her eating than putting all these fancy wonderful things in the lunch box to say I’m a great parent, and I got the carrots and the peanut butter and all that in there, and my kids throw them away at school because they’re not going to eat it anyway.

But letting the kids know what they should eat, and my older one makes a pretty good choice with it. My younger one will probably do a school lunch just because she doesn’t want to make her lunch. They kind of have to take that on themselves.

We’ll guide them as they make their lunch a little bit, but it’s their choice. If they want to eat lunch, they know where the stuff is. They make it themselves, and they get it ready themselves. I mean, if something was happening, I could do, I would do it for them. But for the most part, that’s their, part of their daily responsibilities is getting up in the morning, making their lunch. I make sure they have it when they leave.

But other than that, I’m the bad parent that lets them go to school with a little bit of junk food because to me some food is better than no food. At least I know their bellies are full, and they’ll have energy to do it whether it’s proper energy or not. I know they’ll get the right meal at the end of the day, and they’ll get a better breakfast at school than we have time to do here since we’re also traveling from the Bloomfield School District in the WRV.

So the fact that they can eat school lunch there now is a wonderful thing for me because, one, I don’t have to pay for it which is awesome. But I know they’ll get a little bit more balanced meal I suppose than a granola bar on the drive in.

**MODERATOR 1:** Thanks, Kim, and everybody. Great info. Last question, I think, unless Moderator 2 or Moderator 3 had one, is around, Olivia, you mentioned you used to run some of the, I don’t know if it’s just the snack or some afterschool programming, I’m curious what the four of you, your experience with afterschool programming, wondering if that’s going to be feasible to get any kids there for an hour after school or not even feasible, or anything you can tell me about past afterschool programming, whether it’s been popular, a big flop, or what’s gone on there?

**OLIVIA:** So our after school is for kiddos that maybe are behind, and so they are working with one teacher that stays after until, oh, I think it’s 5:00. Honestly, I can’t remember, 5:30. And they’re with, I think it’s 5:00, that teacher and they have breaks. We, and when I’ve done it, I will only have, I never have more than seven or eight kids. So it’s, to me, it’s been a flop. Sometimes it’s a babysitting service for some parents because they’re not home. So maybe we could entice some of the kids to stay if we’re not just doing what I will call schoolwork. I don’t know.

**MODERATOR 1:** Yeah. How about all four of you, do you think if it, if there were, if there was something fun and the kids wanted to be a part of this cool Hoosier Sport program once a week to start with after school, do you think that’s feasible, or do you think it’s going to be like seven kids, and they’re just the ones getting babysat?

**ANITA:** I mean, I think some of it too is usually there’s a sport after school too sometimes. So I think that cuts into it as well. Because, I mean, unfortunately, the only time we took advantage of the program is when she had to stay for her grade. So, you know, that’s unfortunately how it is.

**MODERATOR 1:** That makes sense. Thanks, Anita. Jasmine, Kim, do you think similarly, going to be a tough sell to do anything after school or something different, if it were fun and enjoyable?

**KIM:** I think it might be a hard sell because as small of a school as WRV is, pretty much, I’m pretty sure all the sports are basically, there are no-cut sports. Like if you want to play on the team, you are going to be on the team. That they’re not going to tell you, oh, you don’t make the cut. I went to a much larger school, so if you didn’t make the team, you just didn’t play.

And that’s different in these smaller schools, where, if the kids want to play, that resource is available to every kid that can make the practices. And if they’re not making the practices, I don’t know that they would make the other afterschool activity. Because like she’s, the one that said, that there already is sports after school, and if you want to play, you can play. There’s not really a skill level that’s the minimum skill required to play on the school sports.

**MODERATOR 1:** Great point. Thanks, Kim. Jasmine, anything to add on that one?

**JASMINE:** I think the afterschool program helped my daughter out a lot. Because I know when, the previous school we went to, she was struggling really bad, and she was making all Fs, and they passed her with anything and everything. No help. No nothing. And I was going to get a tutor and all that crap.

But I switched to WRV, she started going to that program, I would say two or three weeks into it, and she, it took time to bring up her grades, but, I mean, it helped. It helped a lot, and she came a long way from where we used to go to school at. And it’s, I mean, she loves it. I mean, it helps her for learning disabilities, and, I mean, she does sports too, but, I mean, it helped her a lot.

**MODERATOR 1:** Awesome. Thanks, Jasmine. Yeah. We’re looking at it, and we’ll test it out this fall, and that’s the really great feedback. So I don’t have any other questions, but Moderator 2, you have one more?

**MODERATOR 2:** Yeah. I’m wondering if all of you could answer the question of, with the kids that you know or kids that you have or that you’re around, what’s an exciting program look like? So exciting that they’re like, I want to be in that. That’s where I want to be. I want to do that. What does that look like? What do we have? What gets them to say like I know it’s after school, but I want to go because it’s so cool. What do you think some key features are? An example can be like, oh, go ahead.

**OLIVIA:** I feel like I’m over like stepping, but the kids, at least the ones that I’ve been involved with, they like things. They, you know, they are reward-based. They like, I mean, I hate to say that, but they, whether it is some, they have to have that physical, it’s mine kind of thing. So if we can adapt that to the kids that, I think that would be the key for them.

**MODERATOR 2:** Super helpful. Yeah. We like stuff, right? We like goodies. That makes sense. What else?

**KIM:** I might be off base on it, but if the teams are winning, kids are going to want to be a part it. They want to be a part of something, especially if they bring that, the school recognizes their accomplishments in the sports at school during the day that more kids will want to be a part of it. Because, by nature, we all want to be accepted, and if playing that sport and getting on that team is what’s going to bring you acceptance, that’s kind of what a lot of kids will want to do. They want to be part of that winning team.

**MODERATOR 2:** Yeah. Thank you. Connected with others.

**MODERATOR 1:** All right. So 7:58. Awesome. We are right on schedule, and let’s wrap up. So two, I guess, the three things are a reminder that if you didn’t fill out the consent form yet, Moderator 3 will send that link out and the study information sheet. And if you have any questions, you can contact me or her.

The other one is for each of these sessions, we have $40 for each of you. So if you come to all five, that will be $200. But we need to figure out if you’d prefer a physical VISA card or an Amazon eGift card. If you do the eGift card, we can send that out tomorrow and just send it out right after each session. If you do the physical gift card, yeah, I don’t really want to send those in the mail just in case something happens to the gift card, so I think what would work best is I can come to, one of us, or some of us, can come to WRV once a month. So sometime in August and then again in September, and we could deliver them to you that way.

So I just need a preference from you. You can either just say it out loud or type it in the chat whether you’d rather, physical VISA cards or Amazon eGift cards. And we have both, so everyone doesn’t need to agree on the same thing. Just what’s your preference? And Moderator 3, you’ve got this? Cool. Olivia, Amazon, awesome. Jasmine, Anita, Kim, Amazon? Okay.

**JASMINE:** I would do Amazon.

**MODERATOR 1:** Okay. And Anita, okay, cool. All right, everybody Amazon eGift cards. We can probably send those out tomorrow, right, Moderator 3? Cool. So those will come to your email inbox. I think we have everybody’s information, text, and email, and that kind of thing.

And then the last thing is next session time. So, originally, we were thinking do these every two weeks, but I wanted to see if you all might be able to do it roughly weekly, and then if it doesn’t work for a number of people, we can lengthen it out. Because we’re trying to start the program in October, so if we finish these five sessions in August and into September, that would be perfect.

So any chance next Monday at 6:30 p.m. or 7:00 p.m. works for you four? If, I guess, if that doesn’t work, could you put your hand up to let me know? Olivia’s out, okay. Jasmine, Anita, Kim?

**KIM:** I should be good. The fact that they’re Zoom calls, we can kind of do them anywhere as long as there’s a cell signal, so, I’m good.

**MODERATOR 1:** Yeah. Would, did you want to say something, Olivia?

**OLIVIA:** Well, I might be available. Just depends. We’re kind of up in the air right now with my volleyball. We don’t have system, and so we’re practicing at Worthington, so I won’t know until the end of the week.

**MODERATOR 1:** Okay. Is 6:30 or 7:00, does anyone, let’s see, how to best ask this, 6:30 or 7:00, what’s better for each of you in general? Say you were able to come, Olivia.

**OLIVIA:** It wouldn’t matter. Either one would be fine for me.

**MODERATOR 1:** Okay. Jasmine, Anita, Kim?

**JASMINE:** 7:00 would be better for me.

**KIM:** I agree. So the later the better because that way they can get the practices done.

**ANITA:** Right. I should say I agree, so later is better.

**MODERATOR 1:** Beautiful. Cool. All right. So 7:00 next Monday. We’ll go with that. And if we get to see you there, Olivia, awesome. If not, that’s okay too. But we, ideally, we’d like to get, so we have seven parents that agreed to participate. It’s totally okay if all seven are here, but we want to have at least five. So that’s like our minimum goal.

So if we can’t get five, I’ll, I or Moderator will message you and we’ll figure out a different time, but I have a feeling Monday at 7:00 will probably work out. We got three yeses and a maybe and then we’ll let the other folks know. And then just for your own knowledge, the next session is going to be all about solution generation. So we’re trying to think about, of solutions and ideas and which Moderator 2’s question was a great segue into that. Like what would a good program look like? So this is where we, kind of, put the rubber to the road for figuring out what it looks like.

And then on Monday, we’ll have talked with the first kids’ group on Thursday night. So that’ll be cool to give you feedback on how their session went as well. So any questions before we take off, anybody? Beautiful. All right. Watch your inboxes tomorrow. Those gift cards will be coming. Thanks so much. This was incredible info. I totally appreciate all of you, and we’ll talk again soon.

**KIM:** Thank you.

Indiana University Sport Management

Hoosier Sport

Adult Design Session 2

**MODERATOR 1:**  Cool.  Oh, there’s Avery.  Awesome.  Let me catch her up to speed in just a minute.  Hello, Avery.

**AVERY:**  Hello.

**MODERATOR 1:**  Thanks for coming.  I’m just going to catch you up real fast and . . .

**AVERY:**  Okay.

**MODERATOR 1:**  Last week, basically we talked about challenges and issues and problems with physical activity and sports and nutrition and different like resources and things at WRV.  And then this week, we’re shifting to talk about ideas.  So today is all about brainstorming ideas for what the program can look like this coming fall.  And even the program this fall, like we’re going to have to revise and test it and see how it goes.  And we’ll learn a lot, I’m sure, from our first eight weeks at WRV.  But that’s what today is.  Any questions before we keep moving forward?

**AVERY:**  Nope.

**MODERATOR 1:**  Okay.  Cool.  Well, thanks for coming.  Last week, some of the things, just to jog your memory, everybody, that we talked about, we talked about sports programming at the school.  We talked about PE class, some lack of consistent PE teachers or a dedicated PE teacher, favoritism, interaction with coaches, lack of support or help from coaches, frequency of physical education classes, was it enough or not enough, coach training, sport participation, afterschool sports programming.  We talked a little bit about how should parents be involved in the program.  And then, yeah, that was most of our challenges.

So today I wanted to ask about PE class.  So one of the things we’re hoping to do is, even if you’re only going to be there for PE class and after school a couple days a week, if I’m not mistaken, Mondays and Fridays, starting in October.  But we want to try to have a larger impact, because physical activity is just, Olivia, you probably know this best, but maybe it’s a 50-minute class.  And you can only do so much with that amount of time.  So we want to try to influence these kids outside of that time.

So who, would anyone be willing to share some ideas for how to have a broader impact than PE, what that could look like?  For stuff, or ideas, or things they could do that we could maybe teach them, or train them, or show them in PE that they can do elsewhere.  Ideas?

**OLIVIA:**  Do you think we could have like competitions at home, and, because kids love to compete against each other and compete against themselves, what if we did something outside the school, where they like logged in certain fun things for them?

**MODERATOR 1:**  Yeah, I love that idea.  Thanks, Olivia.  Really, really great.  So competitions, yes, could definitely do that.  We could be as creative as we want with those.  I think that would be like a, what, until we hear from everybody, like a must-do in some capacity.  What are some ideas for what that could look like, for what types of things they could potentially be competing in?  And there might be 50 different answers.

**OLIVIA:**  I know, years ago, we used to have like a jump rope-a-thon.  I don’t even know if that’s possible.  They used to do like where they walked and they logged those in.  Those are the things, years ago, we used to do, so just ideas.

**MODERATOR 1:**  Cool.  Have those been at WRV, years ago?

**OLIVIA:**  They used to do a jump rope-a-thon in gym class a long, long time ago.  I don’t think they have that anymore.  I know in sixth grade we do the Presidential Challenge, but that’s just literally one day.  So maybe do something to work up to the day that we have our competition.  We used to do something with pennies, but I can’t remember what we did with them.  So it’s, I mean, just ideas.

**MODERATOR 1:**  I love that.  Great idea and that’s exactly what we’re looking for, ideas.  Monica, Jasmine, Avery, Kim, thoughts on that, on competitions, some things that might be fun for the kids to either compete against themselves, against others?  What kinds of, what does that make you think of, competition?

**KIM:**  I know we had the walk-a-thon, when I was in elementary school and junior high.  Where also, it could also run something like the library does with reading 15 minutes a day to what kind of activity you did for 15 minutes, whether it be running, swimming, jumping, or even an outside sport.

**MODERATOR 1:**  I love that.  What did the walk-a-thon look like in terms of, I’ve heard of, like it could be like laps, or steps, or mileage, or a pedometer, or tracking your own with popsicle sticks, or what did that look like for you guys?

**KIM:**  When I did it, we did it, we got sponsors for however many laps we could do.  And it was anywhere from $.50 to $1.00 a lap and it was always used as a fundraiser.  So no, we didn’t get the money, but it was a fundraiser for the school, so it kind of had a twopurpose.  It got us moving and kept [inaudible, 00**:**05**:**53].

**MODERATOR 1:**  Well, awesome.  And so you would fundraise for a time period, and then there was like a day that the walk-a-thon would happen at like the school track or something like that?  Cool.  Other thoughts, competitions, walk-a-thons, other key elements of those things or other things that you could compete on?

**MONICA:**  I really like the idea of the competitions and the things that you can do at home because that would actually get the parents involved as well.  I know we used to do the little 15-minute things and the parents sign off on the different things that we would do at home.  And it can even be, we did it as like game time.  And it could be physical activity or just family activity.  But I really like that idea.

**MODERATOR 1:**  Great idea.  Yeah, taking it home, getting other people involved, siblings or parents or something like that.  Jasmine, how about you, or Avery?  I don’t, unless I heard from Avery.  I’m not sure.

**AVERY:**  No.  I think it’s a good idea.  I was thinking something even like a, maybe get all the parents together and the kids one night out of maybe that whole ordeal where you do like maybe a sack jumping race with your parents or your sibling.  You know, bring them all together too, maybe just one night out of that week out of everything that you’re doing.

**MODERATOR 1:**  I love that.  Great idea, Avery.  Thanks.  Thanks for sharing.  Jasmine, how about you?  I know you have lots of great ideas over there.

**JASMINE:**  I would say like work out and stuff.  I can’t physically do a whole lot, because I’m pregnant right now.  But, I mean, I would, my husband can do with my daughter and stuff like that.  But anything like competition, I’m, I mean, she likes to do that too.

**MODERATOR 1:**  Cool.  And you’d get triple points for any steps taken.

**JASMINE:**  Oh, yeah.  No.  I’d get like triple, double, and everything all in one because I’ve got two of me.

**MODERATOR 1:**  Okay.  Great.  So hearing lots of good stuff on physical activity-based competitions.  Any ideas for learning or life skills?  Like someone mentioned reading.  Learning life skills, nutrition, things you’ve heard of, or liked maybe that you did that could be similar in a competition-type setting?  And I don’t mean that like as an intense winning competition, but more of a, like inclusive relatedness, friendship, social-connectedness competition.

**JASMINE:**  I think if like my daughter and like her friend or something was doing a competition, it would motivate her more than if it was just me saying, all right, Kristina, go get motivated.

**MODERATOR 1:**  Great point.  Yeah.  It would be, yeah, there might . . .

**JASMINE:**  If there’s more, I feel like if there’s more of them or more involved, then it would be better than it’s just like, I don’t mean this bad, just one child, you know what I mean, doing it.

**AVERY:**  Maybe a trivia-based.  Maybe a treasure hunt around town or something like that where they got to answer the questions and figure it out.

**MODERATOR 1:**  I like that.  Trivia-based is cool.  What could, any thoughts what that could look like?  Because that could be, it could be physical activity trivia, nutrition trivia, local, it could be any trivia, honestly.  But what could that look like?  Like training for a, an event that’s like a trivia competition one day at school or is it like a take-home activities?  What could that look like?

**KIM:**  I suppose a competition or a, like how many steps does it take to get, how many stairs are on the bleachers, or how many steps from the front gate to the bleachers, or from the cafeteria to the gym.  So they have to count their steps as they’re going, kind of an idea how far you are.  [Inaudible, 00**:**10**:**17] scavenger hunt thing [inaudible, 00**:**10**:**19].

**MODERATOR 1:**  Yeah.  I like that.

**KIM:**  So they’re actually doing the steps [inaudible, 00**:**10**:**24].

**MODERATOR 1:**  Thanks, Kim.  Thanks for sharing that.  How about, is there anything you could envision your kids, or kids you’re familiar with, participating for like for this in a nutrition space?  Like trying things at the school lunch, or do you think they would even be into that?  Like should it just focus on physical activity or do you think they’d be open to other types of competitions as well?

**MODERATOR 2:**  Maybe like trying [inaudible, 00**:**11**:**07] sorry.  I don’t want to interrupt.  Go ahead.  All right.  I’ll, I was just was saying that maybe how about having the kids try a new food each week and have a log.  Like, this week I’ve tried pumpkin and next week I’ve tried Greek yogurt.  Because as kids, I know a lot of kids can be picky.  So having a food log like, I tried this [inaudible, 00**:**11**:**33] a competition seeing how many new foods I tried that month or something like that.

**MODERATOR 1:**  Olivia, I saw you shaking your head or thinking earlier, do you think there’s any hope for this in a nutrition space, or should it focus more on physical activity, fun stuff?

**OLIVIA:**  I think it should be nutrition because obesity is widely predominant in little kids and adults.  So if we teach them young, I think it would be great.  I know like one of the health units that I teach is about the food guide pyramid and how many servings in each group.  Maybe we could implement that somehow.  You know, just trying to get the correct, you know, serving.  We do the plate is what we do.  In our book, which is old, it does the actual pyramid and all that, but I teach them with the plate.  I don’t, maybe we could do something with that.

**MODERATOR 1:**  Yeah.  I like that.  Great ideas.  Before we move on, anyone else, anything on competitions?  And Moderator 3 and Moderator 2 included.  Any other thoughts on competitions?

**MODERATOR 3:**  I know something that I used to use a lot when I tutored was those Jeopardy style kind of competitions.  And I know we used those a lot studying, when I was younger.  So I don’t know, maybe if, especially when it comes to kind of nutrition information, getting them prepared to take on a Jeopardy game.

**KIM:**  A game show [inaudible, 00**:**13**:**14] or something.

**MODERATOR 3:**  Yeah.  So it’s got that competition side and they’re also adding facts into their brain.

**MODERATOR 1:**  I like that.  That could even be team-based.

**MODERATOR 3:**  Yeah.

**MODERATOR 1:**  That would also encourage relatedness.

**MODERATOR 3:**  So that would add a whole, camaraderie and all that.

**MODERATOR 1:**  Yeah.  There could be, yeah, individual competition, or a class competition, or a team of any size, whether it’s partners or like three kids or something like that.  That’s awesome.  Thanks for sharing that.  Olivia, the, to my knowledge, there’s, is, there’s two groups of sixth grade kids, right, two classes?

**OLIVIA:**  Yes.

**MODERATOR 1:**  And they’re always separate, or how did the logistics of that look?

**OLIVIA:**  So there’s Mrs. Yoder’s class and then Mrs. York’s class.  I think we each have 20 students in each class.  But each of us see each class together.  Does that make sense?  Not, okay that did not sound right.  So I only see like Mrs. Yoder’s class for math and health.  And she’ll, whenever I’ve got her class, she’s got my class, my homeroom class.  But we don’t intermingle them.  They don’t even go to specials together.

**MODERATOR 1:**  Okay.  That makes sense.  So that’s their unit.  Those 20 kids, they, yeah, they don’t intermingle much.  Okay.  That’s helpful.  Just thinking of like teams.

**OLIVIA:**  Just at lunch.

**MODERATOR 1:**  Right.  Cool.  Do you, question.  Are they randomly selected to those groups?  I’m wondering if it’s ever a bad idea to pit one class against another class.

**OLIVIA:**  So it’s just randomly.  We used to do, we used to go by whatever the fifth grade teacher said.  Who would get the next and so you try to gel the kids together well.  But I know in the past, like I like playing games.  So for like some of the life skills, we went out and we would do, like last year was Mr. Hayes, was the other sixth grade teacher.  So it was Mr. Hayes’ class versus Mrs. York’s class, and we played whiffle ball one day, and we played kickball the next day, and we just did competitions like that.

**MODERATOR 1:**  Cool.  Thanks for sharing that.  That’s helpful.  Other ideas?  So moving on from competitions, what else, and unless something else comes up, cool, competitions, feel free to say it.  When you think of PE class or after school, before school, what are, and we have these groups of college students coming to WRV to teach kids about health and activity and sport and nutrition and life skills.  What are some things you think we would, we should, we need to have within this program?  Things we need to do, or should do, or you think are good ideas?

**KIM:**  Talking about how to deal with adversity during competitions, if you’re not winning, and things like that might be, is that something kind of what you’re looking for?

**MODERATOR 1:**  Yeah, definitely.  Because life’s hard, right?  So you’re like teaching them about those challenges.  And that’s a great one, that sport, like this whole program is designed for sports, like the tool, but within that, we could teach them, teaching them how to handle losing, or getting injured, or just struggles, you know.  Like everyone wants to, whatever.  Maybe they all want to be the starting player on the team, but there’s only so many positions.  So that’s definitely something.  How to deal with adversity.

**KIM:**  It’s even partly teaching some of the kids how to be humble with their winnings.

**MODERATOR 1:**  Yeah.  How to win, how to lose, how to play, like appropriate competitive behavior.  What could that look like to you?  Tell me more about that.  What, how could we operationalize that a little more?  When, like when would you see us doing that, or what could that look like?

**KIM:**  My, the one, out of my two kids, I have one that’s very sports-oriented and is usually, excels at pretty much everything she does.  One of her things, she loves to teach the littler kids and other kids how to do things.  We had a rec softball game where she was pitching and the other girl was in the bat, in the box right.  She stopped the game to put the batter in the box where she belonged and told her how to swing.

And, I mean, she still tried to strike her out, but I mean, she stopped the game just in sportsmanship on how to, because it’s a rec game.  It’s not, the intention wasn’t to annihilate the other team or strike everybody out.  It was supposed to have fun and everybody learn.  So teaching kids that that’s okay in that situation and things like that that you can help other kids.  And by doing that, you’re helping yourself as well, showing that you can teach something along the way.

**MODERATOR 1:**  Oh, I love that example.  Were they the same age, the, in the same grade, your daughter and who she was teaching?

**KIM:**  I think, it might, it was two years, so it was probably a grade below.  But I think it was even a kid from a different school.  I mean that was [inaudible, 00**:**18**:**33] rec league, we played through all the different counties, the surrounding areas here, so for youth softball.

**MODERATOR 1:**  Great idea.  So that sounds to me like some peer mentoring along with like appropriate like sportsmanship.  And could you all tell me more about that, what that could look like, or ideas around that peer mentoring piece?  Like what could work?  What wouldn’t work?  What have you heard of or seen in the past, in terms of helping other kids in, I don’t know, it could be areas they struggle.  Or just tell me more about your thoughts on that peer mentoring.

**JASMINE:**  I just know like I always tell my daughter like something positive or I motivate her, especially when she’s in any kind of sports or anything.  Like it’s okay.  You’ll do good, or just keep your head up and keep looking forward.  That’s one thing I always tell her.

And, I mean, it always helps because she’s always like, I’m never going to finish it.  I’m never going to get it done right, or whatever.  And I’m like, you just got to keep looking forward and keep your head up.  And, I mean, it’s, sometimes it’s hard.  I get it.  It’s not easy.  And just saying little quotes and stuff kind of helps her, and it helped, it helps, I think.  And it helps her, so . . .

**MODERATOR 1:**  Thanks, Jasmine.  Yeah.  Hearing that kind of stuff is helpful.  Olivia?

**OLIVIA:**  I also think, you know, like there’s going to be kiddos that are going to struggle, and there’s going to be kids that are going to excel.  And I know we’re kind of basing this, if I understood this right, like just for the sixth grade.  Is that correct for now?

**MODERATOR 1:**  For now.  And that, and that’s, but that could be flexible if we, if something was in place that affects the whole school or something like that.

**OLIVIA:**  So if we did just the sixth grade, maybe the kiddos that are struggling, pair them up with kiddos that, or may, you know, that understand the concept or understand the skill that we’re wanting and pair them up so you’ve got that team coach, that cheerleader that can guide them and help them.

**MODERATOR 1:**  I like that.  And can you give me an example, Olivia, or anyone else, of maybe a context?  I’m wondering, because like some kids might struggle in one area and be really strong in another area.  Like they’re really great in math and nutrition, but they’re not good at all at sports or something.  And then it might flip, you know.  So could you . . .

**OLIVIA:**  So some kiddos, I’ve noticed in the past, some kiddos that are not very great at sports, they’re really good at designing like a game.  So why can’t we have them design the game or some facts or whatever.  And they like do trials with the kiddos that actually want to do them.

**MODERATOR 1:**  Great idea.  I like that.  Who, what else?  All right, Monica.

**MONICA:**  I think that would help the, I think that would also teach them how to accept and tolerate differences.  And that’s kind of the mentoring thing too is we’re all different.  We all have different abilities, like what Olivia is saying.  So when pairing them up with, like she’s saying, it will help them learn how to accept that and learn how to respect it.

**AVERY:**  They’re not always going to be the best in everything they do.

**MODERATOR 1:**  I like it.  Kim, anything on that one?  Okay.  So with that, do you, a pretty specific question.  Do you think it’s a better idea to have kids paired up in this program in certain ways or have them in small teams of like maybe three or four?  What do you envision?  You all are more familiar with sixth graders than we are.  Which do you envision going better?

**AVERY:**  I thought just pairs.  That’s what I would say again.  I don’t know.  Just more one-on-one with each student with each other.

**MODERATOR 1:**  Thanks, Avery.  How about everybody else?  Pairs or teams?

**MONICA:**  I think it depends on what you have them doing.  There’s going to be some things that they’re going to do better as a pair.  And then there’s going to be some things that, when you’re having to think and plan and do stuff, that would be better with a team.  But I don’t think either one fits in every category.

**MODERATOR 1:**  Great answer, Monica.  It depends.

**KIM:**  It does.  And sometimes, if there’s a group, if it’s a group of three, one could be left out, and the other two are felt carrying, feel like they’re carrying all the weight of the one and not necessarily integrating that one into, help them get better.  So it, it is, I think it does, once again, just comes down to what the activity . . .

**MODERATOR 1:**  Thanks, Kim.  Yeah, I agree.  Great points.  Depends what you’re doing.  Sometimes it might be really good for a pair.  Sometimes you, it might be really complex, and you might need another person or two.  Anything else on peer mentoring before moving on?  Cool.

So I’m hearing peer mentoring strong, competition strong, inside the classroom, outside the classroom.  How about, what, actually, what else do you think is imperative to have as a part of the program along those similar lines of cool ideas you have?  What else?

**KIM:**  Maybe just learning things that they may not ever learn.  That we have all the typical sports, but maybe stride out a little bit and learn a little bit about other sports that they might not know.  Whether it be like cricket or something or badminton, or I don’t, I can’t even think of them.  But just more of the sports that aren’t mainstream.

**MODERATOR 1:**  Great idea, Kim.  Excellent.  Everyone else’s thoughts on that?  Any particular sports that jump out to you?  Do you like that idea?  Do you not like that idea?  Are there other types of new things to learn?  Other thoughts?

**MONICA:**  I think that would be really good.  Because if they learn different things that are outside the box, they might find things that they like that aren’t the norm, which gives them the ability to like things that’s not the same as everybody else.

**KIM:**  And it could help too, if it was something like shuffleboard, where it’s not a who’s really the most athletic and who has the most patience to win or to kind of calculate how much force it’s going to take to move an object.  So some of that is, could be the kids that are . .  .

**MODERATOR 1:**  We heard you until the very end, ‘til, but I think I got the gist of it.  But like inclusive and activities that highlight different types of strengths, different types of skills, not just always speed or how far you can run or whatever.

**KIM:**  Yeah.  Because some things it’s a matter of how much pressure to put on [inaudible, 00**:**26**:**37] sports do take some thought and . .  .

**MODERATOR 1:**  Yeah.  That last part was a little choppy, Kim, but I think we got the gist of it, learning new things.  Other thoughts along this same train of thought?

**JASMINE:**  Like learning things for life.  Like preparing kind of, you know what I mean?

**MODERATOR 1:**  Tell me more.

**JASMINE:**  Only me.  So like know that you don’t have, I mean, you can be competition wise or you can, you know, be good at some things, but you don’t have to like always succeed all the time, I guess.  It’s good to feel that you can like win and lose different things.  Not always have to be a winner, not always be a loser either.

**MODERATOR 1:**  Great point, Jasmine.  Thanks for sharing that.  Other thoughts, and Moderator 2 and Moderator 3 included, any other thoughts on learning new things, exposure to new stuff?  Any more details or thoughts on those?

**OLIVIA:**  So with like life skills, I know my sons have come out and showed the kids how to change a tire on their truck.  The kids watched him do it.  They asked questions of the boys.  We go out in the parking lot.  They usually jack up my truck or one of their vehicles and they do it.  They, the kids love it.  Because some kids are going to excel in academics, some are going to just be hands-on.  And I think if we can implement those things for those kiddos, where maybe they need hands-on rather than sitting at a desk or, you know, being in a gym.  Some, it’s just every kid is built different.  So if we can, you know, do those kind of things maybe, I think it would be kind of cool for the kids.

**MODERATOR 1:**  I love that example.

**AVERY:**  [Inaudible, 00**:**27**:**49] for most helpful or something like a secret award or something that they hand out too for most helpful or, I don’t know, something like that would be good for a kid.

**MODERATOR 1:**  Yeah.

**AVERY:**  Instead of just you won, you won . .  . the very best, you know.  Well, just glad that you were there that you can mentor somebody, help them, and you can win at that too.

**MODERATOR 1:**  Yeah.  Great idea.  Different types of awards I heard.  And, Olivia, hands-on things, definitely, when possible, I love the change the tire example, like applied life skills.  And with the awards, oh, sorry, after you.

**OLIVIA:**  So another silly one.  I noticed some of the kiddos that are going to be in my sixth grade class.  They, I haven’t seen them yet, of course, but I know they did Legos.  So what, you know, why, that would be manipulating their hands.  And who could do like the towers or something, I don’t know, with Legos even.

**MODERATOR 1:**  Yeah.  That’s super creative.  Awesome idea.  Others?  What else?  Lots of good stuff flowing.

**MODERATOR 2:**  But you, this just came to my mind.  So last week you guys mentioned home ec is not, you know, no longer available at school.  How about, I know I’m not, there’s no stoves and stuff, but like learning how to make parfaits or learning how to make overnight oats, like something quick and easy that you don’t need a stove.  That could be also hands-on for the kids that also like that part as well.

**OLIVIA:**  So Moderator 2, this past year we, I bought a griddle, and so the kids did, like I had a griddle at my house, so I brought it in, and the kids made grilled cheese and peanut butter and banana sandwiches for kids.  I mean, but it was just a small griddle that I had at the house, and that was one of my life skills.  We, they made cookies and we put them on the griddle.  It’s just, that’s a great idea with the home ec.

**MODERATOR 3:**  I know we talked a lot about, you know, adversity and perseverance.  And, you know, difference, I think one thing to really think about is tapping into some of these people that are kind of around that we could bring in to talk, you know.  There’s amazing hands-on people that love to do kind of STEM work with kids.

There’s people that, you know, athletes that would love to either Zoom in or come and talk about their adversity and what they’ve done and how they’ve achieved things, but they’ve also failed.  You know, bring in other faces so people can be like, that’s someone cool.  That’s not just like Moderator 2 that comes in every Tuesday and Friday, you know.  Because I remember being really inspired by like a scientist that came in and let me touch a brain.

I’m not saying we’re going to let little kids touch brains, but, you know, like things like that that like, you know, gets their hands dirty, gets new stories into them, as much as we could, you know, that exposure that maybe they’re not getting directly in the classroom or when they’re sitting on the Internet at home.

**MODERATOR 1:**  Awesome idea.  Yeah, guest speakers.  The griddle, very, very cool.  So yeah, trying new foods and what a, yeah, what an important life skill that they can just, that they can have the confidence to cook some basic things.  And that’s definitely lacking, because I see lots of college students on a regular basis.  So that would be very cool, nutritionally.

Could you, so let me open a question for either one.  What are some ideas for, so nutritionally, life skills, or even those guest speaker presentations, what are, what do you all think are some of the most, what do you think are some like attractive topics?  And maybe we start with nutrition, some ideas around that.  And then we could talk about like some cool speaking topics.  So nutritionally, what are some, what do you, what could you see as some potentially good idea topics for kids?

**MONICA:**  I think coming up with recipes that they could present to us with different types of healthy choices rather than junk food choices.

**MODERATOR 1:**  Thanks, Monica.

**OLIVIA:**  And I think nutrition is going to be a hard one for us, because we live in a very fast-paced world.  You know, let’s go to McDonald’s.  Let’s go do this instead having a sit-down meal.  And a lot of kids don’t get that.  But if we can teach them that here’s the pros, you know, versus [inaudible, 00**:**34**:**08] McDonald’s cheese [inaudible, 00**:**34**:**10].

**MODERATOR 1:**  Great point, Olivia.  I do think nutrition will be tough.  It had the, so we asked people about their interests in the survey that we did at the school, the adults and kids, about their interest in nutrition programming, sport and physical activity programming, and life skills programming, and nutrition was the lowest.  So that is, that’s the hardest.  Sport and physical activity was the highest, and in the middle, which was still really popular, was like new life skills programming.

Okay.  How about speakers?  When you envision kids wanting to hear from student athletes, scientists, mechanics, what do you think are some of the most popular things that would be received well or are important?

**OLIVIA:**  So in sixth grade, I had the sheriff come in.  The kids loved that, because he did talk about the drugs in our society, you know, in Green County.  We’ve had EMTs come in.  The kids loved it, because they brought in the dummy, and they got to practice CPR even though they weren’t doing it.  You know what I’m saying?  But they got to have the little dummy person there.

We’ve had dental hygienists come in talking about the teeth and how to get your teeth.  I’m trying to think.  There was one that I did two years ago that was a mechanic.  The kids weren’t real thrilled about the mechanic, just because he just stood there and talked, where the other people brought in props.  Is that, you know, so the kids love props.

**MODERATOR 1:**  [Inaudible, 00**:**36**:**02].

**MODERATOR 2:**  You’re on mute.

**MODERATOR 1:**  If the mechanic brought in a car engine or something, it might have gone smoother.  Yeah.  Great idea.  Hands-on stuff.  Totally.  Other ideas from folks?  Other topics you think would be super good to have somebody come speak on that would be interesting to the kids?

**KIM:**  Maybe something that they had to do differently to get them where they’re going, whether it was sports or science.  What kind of intrigues them to dig into that field whatever they’re doing.  If it’s a student, what they’re going to school, why they chose that as a school subject.

**MODERATOR 1:**  Great idea, Kim.

**OLIVIA:**  And I think about like our culture.  You know, you’re looking at, if they stay in Green County, and they don’t venture too far, you know, you’re looking at agriculture.  We’ve got doctors and dentists.  We’ve got sports therapists, you know, social workers.  These are things that are, that are, nurses that are in our area that were super easy and accessible to us.  That might be something interesting to the kids.

**MODERATOR 1:**  All right.  Awesome.  Thanks for that.  Let’s see.  So one thing I haven’t asked about, that I’m curious to get all your feedback on, that’s a little guided, is goal setting.  So can you tell me, or potentially any ideas for how we could incorporate some goal setting, and if you think that’s a good idea or bad idea, within the program?  And I ask that from my biased view of really liking goal setting.  Thoughts on how that could be incorporated, how you think it would be received, when you think it might be a bad idea or a good idea, any thoughts on that topic?

**KIM:**  I like goals, as long as they’re personal goals, because the whole class, each kid isn’t going to have the same goal.  As long as they can set their own goals of what they’d like to achieve, if it’s something to do with sports, what they’re, running what they want speed wise, if they want to get faster or jump higher.  Each kid just setting their own goals so they’re not, they’re in competition with themselves, not others.

**MODERATOR 1:** Great point, Kim.  Personalized, definitely.  What else is a good or bad part of that goal setting idea?

**JASMINE:**  I think goal setting is a good thing because that keeps them like thinking what, like what positive things I can do, what good things I can do, or what things I can accomplish in life or later.  And it’s always good to have something instead of just be like, oh, what are you doing today?  I’m just going to go home.  You know, it’s good to have some kind of goal or something to look forward to, I guess you would say.

**MODERATOR 1:**  Thanks, Jasmine.

**MODERATOR 2:**  [Inaudible, 00**:**39**:**34] Jasmine, how would you keep the kids on track with their goals?  Sometimes, you know, we don’t, we lose sight of our goals, and a month goes by and you’re not even on track.  So how would we keep the kids on track and constantly reminding themselves like this is my end goal.  Like how am I going to get there in a month’s time?

**OLIVIA:**  I love goals.  And I think one of the moms, I think it was, said, you know, but I think daily, daily and then weekly.  Like you see it immediate.  Do it immediate.  See the long term.  Because if the kids have a goal, and I love that you personalize the goals, however, I think maybe we allow like an adult to set those goals.  Because some kids want to do that bare minimum instead of going a little bit beyond them and trying to reach a little bit more.  So maybe each kid gets a personal goal.  I think it’s a great idea, because you can’t really lump them all into one, because it’s hard.

**MODERATOR 1:**  Great points, Olivia.  Other thoughts on goal setting?

**KIM:**  You could have more than one goal too.  Different levels of the goal.  A truly attainable goal and then one that you’re really shooting for the stars.  This is where I’d love to be and then maybe do smaller ones to show that they’re getting there, which is partly the daily goals.  Just kind of at the start it with what  like they’d like to achieve at the end, and then let them think out, what’s the progress going to look like possibly to get to that goal.

**MODERATOR 1:**  Thanks for sharing that.  Definitely.  Different types of goals.  Really good.  How about any last comments on goals before diving into another topic?  So wondering about, and will, we’re almost done, wondering about before school hours, like an open gym time.  Is that, do you think that’s something that would be feasible, that any kids would be interested in, based on transportation and school resources?  And any thoughts on considering having before school as like a time period we’re looking at at all?

**MONICA:**  I think before school would be a good idea.  Usually they end up just kind of hanging out before school anyway.  So if we had it being a kind of a structured activity, that they could look forward to, I think that would be a good idea.

**KIM:**  [Inaudible, 00**:**42**:**34]

**OLIVIA:**  I think before school, oh, sorry, Kim.

**KIM:**  No.  It’s okay.  I think physical activity before is sometimes a good thing, because it gets, it wears them out a little bit, where they might be able to be more focused in school too, because some kids don’t have that release at any time, and to kind of go in there, whether it be sports or even something early on, as a meditation or stretching or yoga-type thing, where it’s not even strenuous.

**MODERATOR 1:**  Great points, Kim.  Olivia, you had something.

**OLIVIA:**  So before school would be awesome, because sometimes kids are tired, and maybe it like gets their blood going a little bit, whether it’s just something little.  I mean, there’s been mornings literally my kids like are so sleepy.  So we go outside and we just walk a little bit and we do some, you know, jumping jacks just to wake them up.  So morning exercise, or whatever you, whatever you’re having in mind, would be awesome for the kids.  And plus, they sit in the gym.  A lot of them, they get there at 7**:**15 and they’re sitting there for 45 minutes, so . . .

**MODERATOR 1:**  Oh, so a lot of them get there at 7**:**15, and then class starts at 8**:**00.  Oh.

**KIM:**  Well, the way our bus routes are set up, you know, the way it works with our school [inaudible, 00**:**43**:**48] a lot of them do.

**MODERATOR 1:**  Oh, that’s great to know.  Okay.  Anything else on before school?  All right.  And how about Moderator 2, Moderator 3, any other questions?  Any other topics of ideas to try to chat a little bit about?  We had a lot of good stuff come up today, but anything else?

**MODERATOR 2:**  Yeah.  I wanted to go off Olivia’s.  So since there’s 45 minutes prior to school starting, I know when I was younger in school, prior to the school session starting, they had, they would leave out basketballs and soccer balls for the kids to play with.  Is that that, no, it’s not a thing.  That wouldn’t be possible . . .

**OLIVIA:**  Well, it could be possible, but, unfortunately, they have to sit in the bleachers, while they wait for their classes or whatever to be dismissed.  Part of that is because several years ago, the kids got really rambunctious, and some windows got busted.  And was destructive.  There was no guidance or supervision.  There was a lady that does like bus duty, but she’s only one person, and usually it’s the whole place is full.  So if we guidance, you know, it would be great.

**MODERATOR 1:**  All right.  One other, what, that you brought up**,** Moderator 2, a great point, was equipment.  So when you guys think of, so we have money in the budget for sports equipment, nutrition, like activities, food, some things like that.  What would you say that we’ve, and we asked these questions in the survey about like top sport equipment, but what jumps out at you as equipment needs of the school that are, of course, within these early stages, of being feasible?  So rather than like redoing the entire playground or something, some smaller things that are really needed and would be really attractive to the kids.

**OLIVIA:**  So easy equipment, not doing the whole playground, is we lack balls.  We’re talking basketballs, dodge balls, volleyballs.  Any type of ball, we don’t have it.  If we do, it’s very minimal.  There’s no, the equipment is very sparse.  So anything you would do would be awesome.

**MODERATOR 1:**  Cool.  Monica, Avery, Kim, Jasmine, anything that jumps out at you that you think would be a high priority for you for a type of equipment, even if it was a little bigger?

**JASMINE:**  [Inaudible, 00**:**47**:**00]

**AVERY:**  [Inaudible, 00**:**46**:**58]

**MONICA:**  [Inaudible, 00**:**46**:**58].  Go ahead with that.

**AVERY:**  Netting, like badminton.  I mean, just anything.

**MODERATOR 1:**  That makes sense.  Totally.  Badminton nets or pickleball nets.  I do love pickleball.  Monica, Kim, Jasmine, piece of equipment that jumps out at you as being super important?

**MONICA:**  I was just going to say I’m with Olivia.  Just about anything that you would do to give them something to be occupied by is going to make a difference.  Because like she said, they’re kind of limited to what they can do when they don’t have [inaudible, 00**:**47**:**37] they don’t have staffing to be in there to do all these activities.  But at the same time, showing them that somebody is going to initiate them to do stuff is going to make a difference as well.

**MODERATOR 1:**  Thanks, Monica.  All right.

**JASMINE:**  [Inaudible, 00**:**47**:**54]

**MODERATOR 1:**  Oh, after you, Jasmine.

**JASMINE:**  Basketball was a big thing, so . . .

**MODERATOR 1:**  Okay.  All right.  So Moderator 3, Moderator 2, anything else?

**MODERATOR 3:**  I don’t know if anything like this exists anymore.  That’s why I like, you guys have kids and I don’t.  But when, I remember when I was in gym class, when I was younger, it was very cool to get like a Dance Dance Revolution mat.  Is there anything that is like activitybased and cool that may be in that like realm of maybe equipment?  Just as like an idea because I don’t know what that is anymore, but, you know.  Do you [inaudible, 00**:**48**:**46].

**AVERY:**  I have a sixth grade kid, and I can’t keep up with what’s cool.

**MODERATOR 3:**  Okay.  Yeah, [inaudible, 00**:**48**:**51].

**MONICA:**  You can get DDR mats on Amazon still, just so you know.

**MODERATOR 3:**  That could be a thought.

**MONICA:**  Yeah.

**OLIVIA:**  You know, TikTok is huge for the kids.  What if, you know, you would teach them TikTok dance?

**MODERATOR 3:**  It wouldn’t be me personally, but yes.

**MODERATOR 1:**  I’m sure you could learn, Moderator 3.

**MODERATOR 3:**  You’ve never seen me dance.

**MODERATOR 1:**  I love that.  Good idea.  All right.  So 7**:**55.  Thank you all for sharing the awesome ideas.  I mentioned at the beginning we’ll send out a very smooth-to-fill-out survey tomorrow night or Wednesday morning that’ll be like asking just for your like one to ten, how good of an idea or how important of an idea do you think this is.  Like getting a DDR mat or, you know, something like those types of things.

And then does next, what I was thinking is it would be awesome if we could meet again next week.  And then I was thinking to probably take one week off so we could process a bunch of this information.  Does that sound good to everybody, if we meet next week and then take a week off after that?

**JASMINE:**  Yeah.

**AVERY:**  Yep.

**OLIVIA:**  What day are you thinking?

**MODERATOR 1:**  Monday at 7**:**00 maybe.  Nice.  Looks good.  Awesome.  Okay.  So Moderator 2 will send out a Monday at 7**:**00 invite for everybody.  That’ll be session number three of five.  And then likely, after we debrief and break it all down, we’ll probably take a week off after that to process some of the awesome info we’ve gotten so far.

And then, just for your own knowledge, we’ll be moving towards, session three will be evaluating ideas.  And then we’ll be moving towards operationalizing it into like what the actual program looks like and days of the week and times and how many staff are needed, and equipment we need to purchase, and just all those details.  So like curriculum like, okay, so if it’s important to teach them about healthy physical activity, what do we actually teach them?  And we’ll, that will give us a little bit of time to create that stuff, before we’re planning to start in mid-October for that first-eight week program.  So any questions about that logistic stuff?  Olivia.

**OLIVIA:**  I do know, I want to tell you that we went to a different special schedule meaning like, so they go to PE for a full week.  Then they will go to art for a full week.  So the kids won’t rotate back into like PE.  It could possibly be like four weeks before they go back into PE.  So we did start a new special, I don’t know if Mitch talked to you about that or not.

**MODERATOR 1:**  We did talk about schedule, and it seemed like that was the good time.  So, but I’ll reach out back to them.  And we meet later in August and then probably again in September.  So thanks for letting me know that, because, yeah, we want to make sure that class is going on.  But a whole, a huge thing about this is like that might be a time that we’re there and some after school.  But we want to try to have an impact outside of that class time too.

So as you’re all processing our chat today and thinking in the future, think of that, because like the 50-minute timeslot, we can only do so much.  But we want to try to have a bigger impact throughout the day and weekends or whatever.

So also, the gift cards, ever, did that go, did that not go well for anybody?  Is it, it was smooth?  Okay.  Cool.  So we’ll do the same thing again.  Moderator 2 will send them out before tomorrow night.  And then, and so Amazon eGift cards.  And then, Avery, will connect, Moderator 2 will connect with you, and we’ll make sure you and Braxton are getting the physical VISA cards.  And just in case anyone wants to switch, change your mind, you can do either option.  Amazon eGift card or VISA physical gift card.  But if we don’t hear from you, we’ll just stick with the same one and keep going throughout the process.  Sound good?  So Avery and Moderator 2 will connect.

**OLIVIA:**  Moderator 2, [inaudible, 00**:**53**:**11].

**MODERATOR 1:**  What’s that?

**OLIVIA:**  Moderator 2, are you on there?  She’s [inaudible, 00**:**53**:**18] I just got my eGift card.  She’s that good.

**MODERATOR 1:**  Dang.  Moderator 2 is good.  Moderator 2 and Moderator 3, we don’t, slouches don’t get into helping with Hoosier sport.  So they’re awesome.  So really good.  All right.  Well, thanks, everybody.  You’re all awesome.  If you have any questions anytime, feel free to message me or Moderator 2 a text or email or call or whatever.  I appreciate you taking the time to connect with us and just super valuable information.  And we’ll see you all again next Monday.  And a couple of you, we’ll see your kids on Thursday at 7**:**00.  Thanks, everybody.

**MODERATOR 3:**  All right.  Have a good night.

**MODERATOR 1:**  Bye-bye.

Indiana University Sport Management

Hoosier Sport

Child Design Session 2

**TAYLOR:** . . . is that, you know, we get to play games and, you know, we get to have some free time, you know, like on Fridays.  And basketball we sometimes play.  Soccer some, we play sometimes.  And the bad things that I’ve heard and I’ve like, I’ve heard people talk about is that our coach or our teacher just like just lets us do like whatever we want, you know.  And so I've seen these two kids.  They were goofing around.  And supposedly they got, like one got pushed, and a couple other people got pushed and knocked over and pushed to the ground.  And the teacher actually saw that, didn’t even do anything.

**MODERATOR 1:**  Oh, that’s no good.  So I’m hearing fun games are good.  You like the teacher to be engaged, so actually like paying attention, and if something went crazy, that they should potentially step in to like kind of fix that?  Cool.  Thanks for sharing that, Taylor.  Leah or Casey or Morgan, how about you, what are some of the most fun, best memories, best activities kind of stuff from PE class?

**LEAH:**  So when we played dodgeball, it was fun, because I just, I stood there, and nobody threw the ball at me.  So I would just like catch it and throw it.  I would get somebody out.  I’d just stand there right at the front, and nobody would get me.  That was fun.  That was real fun.

**MODERATOR 1:**  That sounds fun.  Dodgeball is awesome.  Thanks for sharing that too, Leah.  How about Casey, Morgan, some positive memories or some cool activities from PE class?

**MORGAN:**  Just, this is Morgan.  It’s just having free time.

**CASEY:**  This is Casey, and it’s probably capture the flag.

**MODERATOR 1:**  Oh, capture the flag.  Cool.  Tell me about that.  What’s capture the flag look like at your school?

**CASEY:**  It would be like the pennies, and then you go, and you'd have to pick them up and run across the basketball court to get to them to the other side.

**MODERATOR 1:**  Oh, I like it, yeah.  So you're usually in the gym?  What I’m hearing loud and clear are games.  Games are fun.  Very cool.  How about, tell me a little bit more about free time.  So I’ve heard, sometimes positive, sometimes negative, but I remember someone last session was talking about he preferred games that were organized over free time.  Could you, could someone tell me more about that, whether you like the free time, don’t like the free time, or if you'd like it if there were certain rules or something like that?

**TAYLOR:**  I think free time at school is fun, because I think my grade is responsible enough not to get each other hurt.

**MODERATOR 1:**  That makes sense.  Yeah, you need to, yeah, you don’t want anyone picking on anyone or something, like there’s no supervision or none of that.  How about with free time, could any of you tell me a little bit about what equipment is usually available during that free time or what some of the activities are that you do during free time?

**TAYLOR:**  Like in free time, we usually just mostly get like whatever we want out.  I usually get like a ball and, you know, play basketball.  Or, but like everybody either gets like a ball or like hula hoops or like softball catching, you know, with a ball and, so.

**MODERATOR 1:**  I like it, Taylor.  Yeah, so pretty much anything that’s available, you can take it out.  That’s awesome.  How about, so let’s put our creative hats on.  And Riley just walked in, so let me actually catch him up real quick, and then we will keep moving on.

**WOMAN:**  . . . or something like that.  And the mac and cheese will be done in a few minutes.

**MODERATOR 1:**  Hey, Riley, what’s going on, dude?

**RILEY'S MOM:**  Hi, this is Samantha.  Riley is not feeling real well tonight, but if it’s all right, can we still listen in so he doesn’t miss anything?

**MODERATOR 1:**  Oh, yeah, totally.  And then if you have any, if he has any ideas or anything, feel free to chime in or send it in the chat or anything like that.  Yeah, definitely.

**RILEY'S MOM:**  All righty, thank you.

**MODERATOR 1:**  Yeah, no problem.  Okay, so Taylor, Leah, Casey, Morgan, how about creative hats?  If you could design your dream PE class, what would that look like?

**MODERATOR 3:**  I can talk about my dream PE class if you want.

**MODERATOR 1:**  Oh, yeah, let’s hear it.  Totally.  Yeah.

**MODERATOR 3:**  Well, my ideal PE class would have, everybody would participate, unless they can’t for some reason, but everybody will be together.  And the professor or the teacher will give us the instructions, and we can get to choose the games.  It would include games, and it would include, well, depending on what we’re going to do, but I would like to do something active.  Like we can run, or we can jump, not just like sit down and talk, so something that challenged all of us, but it’s safe.

**MODERATOR 1:**  Thanks, Moderator 3, great ideas.  Leah, Taylor, Casey, Morgan?

**TAYLOR:**  I think what my, you know, dream gym would be like, I think it would have, you know, like everybody, you know, participating, and, you know, like whatever we have, maybe some basketball goals, different toys for other kids to play with, stuff like that.

**MODERATOR 1:**  Yeah, great idea.  Lots of participation, different options for different people, those are awesome ideas.  Leah, how about you, best PE class ever, what would you do?

**LEAH:**  So I would have the teacher, or the PE teacher would actually pay attention and do stuff with the kids and stuff.  And then like I would like there to be water balloon fights and stuff like that, like if you earn a water balloon fight or something, you know.  And then definitely would have dodgeball.  I would have capture the flag but with flags.  I think that would be about all.

**MODERATOR 1:**  Dang, that’s a good-sounding class.  Great ideas, Leah.  A couple things I want to ask you about.  You mentioned teacher paying attention.  Tell me about that.  What makes you say that?

**LEAH:**  Because our teacher at our school, he, if we’re playing dodgeball or anything, he’ll just sit on his phone the whole time.  Then . . . but he won’t do anything else.  He’ll just sit there on his phone the whole time on the bleachers.

**MODERATOR 1:**  Thanks.  Yeah, that’s an awesome idea.  So I would think once we launch this Hoosier Sport program at your school, it might be a good idea to consider that the college students and the people that come in to teach the program aren’t on their phone while they’re working with kids.  Great idea.  Awesome to hear it from you, though.  Like I think that sometimes adults don’t realize that kids are watching, and you’re always watching.  So great observation, Leah.  Casey and Morgan, anything to add on dream PE class?

**CASEY:**  No, not really.

**MORGAN:**  Not really.

**MODERATOR 1:**  Okay, cool.  So how about learning?  So games, totally, participation totally makes sense.  Can you all tell me a little bit about what you’d like to learn in either like PE or even like health class?  Because although we definitely want to play tons of games, we also want to learn some stuff.  So are there some things you’d like to learn about related to PE or health that you could potentially learn in a program like this?

**MODERATOR 1:**  What are you thinking, Taylor?

**TAYLOR:**  Well, I would like to learn some, a little bit about, have you ever had like two subjects into one?

**MODERATOR 1:**  I think so, but what are you thinking?

**TAYLOR:**  I’m like thinking, you know, like health and something else or math and something else.

**MODERATOR 1:**  I like where you’re going with it, like where you’re going with that, combining classes, combining different subjects.  Leah, how about you, anything you’d like to learn in health or related to PE or physical activity or anything that comes to mind?

**LEAH:**  I don’t know, actually.  Actually, I might, so you know phobias, right?

**MODERATOR 1:**  Mm-hmm.

**LEAH:**  I want to know which ones are which and everything.

**MODERATOR 1:**  Oh, that’s a cool one.

**LEAH:**  Like because I’m afraid of clowns and stuff like that.  So I don’t even know what phobia that is.  I want to know what phobia is.

**MODERATOR 1:**  I love that.  That’s such a creative idea . . .

**LEAH:**  And I’m also afraid of small spaces.

**MODERATOR 1:**  And, sorry, say that last part again?

**LEAH:**  I’m also afraid of small spaces.

**MODERATOR 1:**  Cool.  Yeah, so claustrophobia is that one.  And there’s probably a name for clown phobia.  But, yeah, that’s a great idea.  Why not, learning about phobias?  Maya, what’s up?

**MAYA:**  I just joined, I think . . .

**MODERATOR 1:**  Thanks for coming.

**MAYA:**  Sorry I’m late, because like I got busy trying to clean out . . . so.

**MODERATOR 1:**  Hey, no worries.  We’re happy to see you any time.  Thanks for coming.  So just to catch you up, Maya, you can hear me all right, right?

**MAYA:**  Yep.

**MODERATOR 1:**  Cool.  So last session, really quickly, last time we talked all about like challenges and problems and issues with PE and physical activity and nutrition and stuff.  Today, basically our whole theme is brainstorm ideas.  We’re trying to come up with as many ideas as we can.  We’ve been talking a little bit about PE class.  So let me ask you first, if you’re okay with that, what would an ideal or like a dream or like the best PE class look like to you?

**WOMAN:**  You need to eat, baby.

**MAYA:**  Well, it wouldn’t be a dream or anything.  It would be like to make it like better.  Like if we like cancel out the mile every Friday and add more exercising games into gym instead of the mile, because like I don’t like running that much.  Like I hardly run like on my own anyways.  So like it would be best if we just like . . .

**WOMAN:**  . . .

**MAYA:**  . . . the mile every Friday and add more exercising games.

**MODERATOR 1:**  Yeah, great idea.  Yeah, running is not for everybody.  That is . . .

**MODERATOR 2:**  And when you say the mile, are you graded on the mile?  Are you, like do you have to get a certain time when you run the mile?

**MAYA:**  No, we have to, once we run the mile, we have to like, we can’t stop running at all for any breaks or anything.  We got, we can’t stop running until our mile is over.  And like that’s what kind of bothers me, because like I have trouble breathing while running, so.  And I don’t know.  I think we get graded for the participations in the games we play.

**MODERATOR 2:**  I see.

**MODERATOR 1:**  Thanks, Maya.  On that note of running, one of things we're hoping to include in this program at lot is something called autonomy.  That’s where you have like choice in activity.  And someone mentioned like it would be nice to have different equipment options.  Maya or anyone else, if, so instead of running, what’s another physical activity you might prefer to do over running?  Because I totally get where you’re coming from with not running to run a mile all the time.

**MAYA:**  I mean, like at my old gym, we had, okay, at this gym, we only play like two or three games, and then we like restart it, go back over, and like start at the beginning.  Because we play dodgeball.  We play . . .

**WOMAN:**  Where you want to lay down . . .

**MAYA:**  . . .and then . . .

**WOMAN:**  Right there?

**MAYA:**  . . . yeah, basically that’s it.  And then like I’d like rather have more games instead of running, because like we can add, like in my old school, I had like kickball, dodgeball, football, tennis . . .

**WOMAN:**  Baby, I got to eat.

**MAYA:**  . . . and stuff like that.  I had volleyball and everything all day, like more exercising things than . . .

**WOMAN:**  . . .

**MAYA:**  . . .down to two games and a mile every Friday.

**MODERATOR 1:**  Thanks, Maya.  Great example.  So let’s shift gears away from PE a little bit.  So we’re going to be able to come to WRV two days a week for eight weeks, which is going to be awesome, for this first initial program.  But we want to try to have like other times of day where you all are being active and learning cool stuff.  So when you think of after school, can you tell me about some things y'all like to do after school?  And especially if they’re related to physical activity, but really just anything you like to do after school.

**MAYA:**  Playing outside.

**MODERATOR 1:**  Cool.  What kind of stuff outside?

**MAYA:**  Well, when I got home from school today, I played, well, I didn’t really play.  I was like mowing the yard and stuff, and I was cleaning out our pool.  And now I’m building . . . for our pool, well, was building, but now I’m not.

**MODERATOR 1:**  Cool.  Most of that is outdoors, right?

**MAYA:**  Yep, every, all of it was.

**MODERATOR 1:**  Oh, beautiful.  That makes sense.  Yeah, it’s, especially after a long school day, nice to get outside.  How about Casey, Morgan, what do you two like to do after school?

**MORGAN:**  This is Morgan.  I play tons of sports, so I usually play sports after school.

**CASEY:**  Yeah, same, I usually do sports after school.

**MODERATOR 1:**  Cool.  What sports do y'all play after school?

**MORGAN:**  I play travel softball, and I do cross-country, not cross-country, soccer.

**CASEY:**  I do soccer and basketball, but basketball is not going right now.

**MODERATOR 1:**  Nice.  So good point.  You guys play a lot of sports, and I bet a lot of students do.  And then sometimes there’s like an off season or like a transition time between sports where you don’t have that.  Could you tell me an activity or two you like to do after school in the off season or between sports?

**CASEY:**  I like watching movies most of the time.

**MODERATOR 1:**  Awesome.  Leah, Taylor, how about you two?  After school, what are some things you like to do?

**LEAH:**  I like to play with Legos.  Like I said that before, I like to play with Legos.  And I’ll only watch a movie if I get to pick it, because I'm just like that.

**MODERATOR 1:**  I love that.  Thanks for sharing that, Leah.  Great.

**WOMAN:**  Mac and cheese is done.

**MODERATOR 1:**  How about you, Taylor, after school, what are some things you like to do?

**TAYLOR:**  Well . . .

**WOMAN:**  . . . ball . . . ball.

**TAYLOR:**  That’s my mom, sorry.  Taylor.  I like, well, we’re usually like mostly in the cafeteria, you know, eating a snack.  But we sometimes go to the gym and play with like the after-school teacher.  They take us to the gym, and we can play.  And we either try to find a game, or we have free time.

**MODERATOR 1:**  Awesome, Taylor.  Thanks.  How about Moderator 2, Moderator 4, Moderator 3?  Two things for you.  Any question you have that comes to mind or any activities after school you’d like to share that you enjoyed when you were, whether it’s in college or high school or middle school or anything like that?

**MODERATOR 2:**  I’m going to go off Taylor.  When you say free time, it sounds like everyone gets a lot of free time.  Do you like free time?  Do you prefer free time?  Would you rather have that time structured with someone giving you a game to play or something along those lines?

**TAYLOR:**  I’m not really like, you know, having like a, let’s just have a free day.  I would like to, you know, get my energy going and, you know, maybe have a game here and there.

**MODERATOR 2:**  Would you say free time is usually what happens when it comes down to being active at school?  It’s a tough question.

**TAYLOR:**  . . . it is, yes.  But I just think whenever it does come down to free time, you know, I’m, you know, it’s an iffy-iffy okay.  You know, I’m not really okay with it, but I’ll still do it.

**MODERATOR 2:**  All right.  Thank you, Taylor.

**MODERATOR 3:**  For me, I have a question.  When you say your teacher is sometimes on the phone or he’s busy or whatever, what do the kids do?  Do, does he organize like a game for everybody to play and you guys play on your own, or he lets you do whatever you want to do, and he leaves a lot of toys and equipment that you can use while he’s doing what he’s doing, or how does that work?

**TAYLOR:**  Well, we first go to the gym.  You know, we sat down.  We, you know, we all . . . a game sometimes.  But what he usually does is he, we sometimes, though, it’s not, we, like he just says, just go do free time.  Sometimes he’ll just like say, you can have free time, you know.  You can do, you know, you can play with all the equipment.  And then he just sits down on the bleachers and play with his phone.

**MODERATOR 3:**  Okay.  Yeah, that’s good to know, because now we can make a plan and prepare some things to do altogether.  Do you prefer playing with classmates, or do you prefer playing alone?

**TAYLOR:**  I actually like to play with classmates.

**MODERATOR 3:**  Right.  And do you have, have you noticed if your classmates all get along together or there’s issues that they don’t want to play together, or they have no problem with doing activities together?

**TAYLOR:**  Well, they, some kind of get along.  Some don’t.  But I mostly get along with everybody.  I think Leah, I think she gets along pretty well with everybody.  I don’t know.  But I just see her Monday through Friday, and I kind of see that she interacts with, you know, other classmates and different stuff.

**MODERATOR 3:**  Okay.  That’s cool.  Thank you.

**MODERATOR 1:**  How about some others?  Let’s go off that question of Moderator 3’s about working together, playing together, getting along.  Can you all give us some ideas for some activities that you’ve seen go well with people working together, whether it’s in pairs or teams, or on the flipside of that, activities that haven’t gone well, like where that you’d avoid teamwork on?  What you thinking, Leah?

**LEAH:**  So I do get along with most other people, some not so much because Jordan, he is a troublemaker.  I do not get along very good with him.  Taylor . . . I get very . . .

**TAYLOR:**  Same with me.

**LEAH:**  . . . I, yeah, I get along good with Taylor.  So I get along good with most of the people.  But some people, like Jordan, I do not get along with.

**MODERATOR 1:**  What does that mean?  I have to ask.  What is it?  What are some of the things that Jordan does that are like no . . . good?

**LEAH:**  I don’t know.

**MODERATOR 1:**  You don’t have to share anything you don’t want to.

**LEAH:**  Nah.  Well, it’s fine.  I’ll tell.  So Jordan, sometimes he’ll just speak out loud.  Sometimes he’ll say stuff like . . .

**TAYLOR:**  Like just random.

**LEAH:**  . . . he thinks he’s being funny.  Yeah, and he’s annoying the teacher and everything by doing that.  And I do not, this one thing I do not like about him, he cusses a lot.

**TAYLOR:**  Mm-hmm.

**LEAH:**  Not in school, but he cusses on the bus, stuff like that.  I just think he . . .

**MODERATOR 1:**  Thanks, Leah.  Thanks for sharing that.  So that’s helpful for us, because we want to know what behaviors to avoid.  Classic boy thinking he’s funny is no good.  How about, I’m thinking of like being inclusive.  We want everyone, we want as many people to feel as welcome as possible in this program.  So can you tell me any ideas, any examples from the past of things that you suggest we do to try to help all the kids in the program feel welcome and feel a part of the group and feel, yeah, feel welcome and included?  Tell me a little bit about that.

**LEAH:**  You want me to do it or . . .

**MODERATOR 1:**  Sure, yeah, go for it, Leah.  And then we’ll come to you, Taylor, and then we’ll go to the others.

**LEAH:**  I just think maybe we could like get to know each other better, you know, say more stuff about us, say what favorite color, how many pets you have, what pets do you have, their names, stuff like that.

**MODERATOR 1:**  Awesome ideas.  Awesome.  Taylor.

**TAYLOR:**  I think we should like not go like so big, because, you know, people go so big, where like I think we should just like, you know, talk to them, communicate a little bit, see what they like, you know, because maybe some people don’t like going big.  Some people might be, you know, liking, oh, I just really want to communicate with this person instead of, oh, not just a huge welcome sign and saying, oh, my gosh, you’re actually here.

**MODERATOR 1:**  Thanks, Taylor, great ideas.  This is really good.  Yeah, getting to know each other is like so important.  How about Maya, Casey, Morgan, can you tell me some ideas or some things you’d like to see so that we make sure everybody feels included?

**MAYA:**  What are you talking about when you say . . . like game-wise, or?

**MODERATOR 1:**  Not necessarily.  Anything.  It could be in games.  It could be in like a learning activity.  It could be even just to be a part of the program.  Anything that, and it might be something that you guys have experienced in the past before.  We want people to feel, we want kids to feel included.  So that could be something that you didn’t like in the past that you learned from or just some ideas for us to help us make everybody feel included and welcome.

**MAYA:**  Yeah, like in gym class, I would like to see more like positivity and like for the other girls to be like actually kind of participate instead of like, because there’s like these two girls in my gym class that weren’t like participating.  They like stand up and pretend they’re playing the game, but they'd like, when we’re playing dodgeball, they'd hold on to the ball or something and won’t throw it until like all of us are out.  And then they like go up to the line to get hit on purpose and stuff.  Like I would like to see more like participation and stuff and like more kindness, more active, positive, and like just more like, more energy in gym instead of like so calm.

**MODERATOR 1:**  I love that, Maya, energy and enthusiasm.  Do you, can you think of an example, by chance, of a time where you have felt either a student or yourself or a teacher or something brought, like was kind, like brought good enthusiasm or good energy to the class?  Can you tell me what that looked like a little bit?

**MAYA:**  Yeah, there is this teacher.  I think some people in here know who he is.  His name is Mr. Williams.  And he like, every time where I’m in his class, he likes walks in, and he starts clapping his hands and stuff, and he starts like shouting out people’s names and saying like what’s up and stuff.  And like he like, he’s like a good, energizing person to be like around and play.  Like one day, me and him like and the class went outside, and he joined us in basketball and everything.  He like, he isn’t one of those teachers like that like stay back and talk to the other teachers.  He’s like one of the ones that like to do what kids are doing instead of what the adults are doing.

**MODERATOR 1:**  Oh, that’s so good, Maya.  Can anyone else tell me a similar story, whether it’s him or someone else, positive energy?  Leah.

**LEAH:**  So Ms. Morguson, she was a very, very, very nice teacher.  Like I can’t even explain how . . . was. Like she understanded like all of the kids.  Like she, if anybody wanted her to say like, I don’t know, like people say like bro, stuff like that, you know.  And then if they wanted her to say that, she would say it, and it would make everyone laugh.  And she’d say, oh, I’m not doing that again, but she does it again anyway . . .

**MODERATOR 1:**  . . .

**LEAH:**  She was a really, really fun teacher.

**MODERATOR 1:**  Great story.  That sounds super important.  Let’s keep this momentum going.  Casey, Morgan, or Taylor, can you share another example of a teacher or an experience or another student or yourself that’s like made it either fun or inclusive or welcoming?

**TAYLOR:**  So I like Mr. Williams.  So one time I actually had to stay after school.  He was my teacher for after school.  He, you know, he, he’s really fun to hang around with.  He really is.  He kind of lets us, kind of let us do what we want, but not all the time.  He’s not really strict, but he’s strict, you know, not taking advantage of him.  But I like how he is very like coming in and calling out people’s names and saying, oh, hi, how’s your day?  Did you get your energy going yet?  Or, you know, he would sometimes make a joke or two.  But that’s the teacher that I, not really like the most, but Ms. Morguson.

**MODERATOR 1:**  Thanks, Taylor.  It sounds like they really care.  That’s super important.  Casey, Morgan, how about you, any ideas for how we can make the program welcoming and inclusive and bring that good energy?

**MORGAN:**  No, not really.

**MODERATOR 1:**  All right.  Okay, that was excellent info.  How about let’s go, so we’ve got about ten more minutes or so.  I’d like to ask a little bit about competitions.  So with our adults, we had a Zoom session like this with a group of parents and teachers earlier this week, and they had some ideas around competitions and things.  So some things, like there’s a huge range of competitions, from like sporting games to things like walkathons or jump ropeathons or like walking events, things like that.

Are there any, I’d like for you guys to share some thoughts on competitions and some things that you might like or dislike about them.  And the competition, keep in mind, can be against yourself, or it can be like in teams against others.  Any ideas there?  Taylor.

**TAYLOR:**  So whenever I played soccer, the coach was like, we had like, we’ve never lost a game.  Like it was that competitive, that we like didn’t lose a game at all.  So, you know, I think it’s good to kind of lose and not just, oh, you’re a winner every single time.  I mean, you really don’t have to be a winner every single time.  It’s good to fail.

**MODERATOR 1:**  Yeah, great point.  It's good to learn how to win, good to learn how to lose.  And it's part of life.  Leah?

**LEAH:**  So I kind of lost track of what I was saying, what the subject was.  Hold on.

**MODERATOR 1:**  Competitions, any thoughts on things you’d like to see, whether it’s competing against yourself or others.

**LEAH:**  Yes.  Chess competitions.  So I want there to be like a chess club, where we could play against people, practice and everything, because I used to practice a lot in Ms. Morguson’s room, so.

**MODERATOR 1:**  Great idea.  Yeah, chess club, chess competitions.  It doesn’t, so this Hoosier Sport program, even though sport . . . in the title, it doesn’t need to all be about sports.  We want to teach life skills and leadership and goal setting and that kind of stuff too.  So chess is a awesome example.  Maya, how about you, thoughts on competition against yourself or others, some things that could be fun or some things in the past you might have liked, some things like that?

**MAYA:**  I’m not quite too sure.  When I was playing volleyball, I kind of liked how they weren’t like too pushy about it.  They were like calm and chill.  They actually like kind of went along with what some kids were saying and stuff, instead of like most people.  They were, most people are like, they push you to like keep going and stuff.

Like these coaches were like really nice, and they like let us stop, take at least one or two drinks, and then get back to our game.  And like they like, instead of like my last coach, my last coach was like making us put everything up after they had gotten it all out.  But they help us put this up, the volleyball net and everything, and so they’re like having us do everything.

**MODERATOR 1:**  Cool.  Thanks, Maya.  How about related to the competitions, whatever, whether it’s chess or soccer or walking, anything like that, what do you guys think would be some good, what’s the word, like awards or like prizes that are, I don’t know, feasible but that would be cool to you?  What are some, what would you like to win if you won a chess competition or if you, I don’t know, beat your, like if you had a step goal to get 5,000 steps in a day, and you did it for a month or something?  What are some prizes you might like to have or get?  Leah.

**LEAH:**  I would like, so whenever I play travel softball, I get these rings.  They’re big.  They’re really big rings, and they have like fake diamonds on them and everything.  And they’re just so cool.  And I think that maybe in some of those competitions, we could get some of those, because I just think those are so cool.

**MODERATOR 1:**  Awesome idea.  Other thoughts on prizes?

**TAYLOR:**  Maybe like, you know, maybe like, you know, maybe like if like, say that I won a game, you know.  How about for a prize, how about like a like $20 gift card to Dairy Queen or Subway or, you know?

**MODERATOR 1:**  Yeah, thanks, Taylor.  Great ideas.  Gift cards are never a, they’re always a fan favorite.  Maya, Casey, Morgan, prizes you might be motivated by or interested in if you won or if you like beat, got, reached your goals, whatever those goals are?

**MAYA:**  Maybe like a celebration thing to like congrats us by, when we win or something, because I’m not the type of person that like asks for things, for like prizes or something.  I just like to be like celebrated for stuff like that.  Because like I know people don’t have the money to be out buying things, because like they got bills and stuff.  So like I could just be like, come home and like maybe like choose dinner or something to be celebrated or something.

**MODERATOR 1:**  Awesome ideas.  Yeah, they don’t need to be, they don’t always need to be like material things.  That’s a great example, Maya.  Moderator 2, Moderator 3, any questions, lingering questions you’d like to ask?

**MODERATOR 3:**  I wanted to give an idea for competitions.  I didn’t know how to raise the hand, so I was just like waiting for you to ask.  I would love, I remember when I was in elementary school, we did a competition that it was military type.  And I remember they put some mud and some ropes like in . . . and you got to go under the ropes, and you'd get all with mud and dirty.  And then you have to climb something, not very tall because we were kids, but we climbed something.  And then when you got on the other way, you have to . . . this big tire of a truck, like this, and with your teammates, and you have to roll that tire to the finish line.

I don't know.  It was like a circuit, I guess, of different things that were not sports.  They were like skills.  It was different skills.  And I think that was very fun.  Until this day, I remember it very clearly, and I truly enjoyed it, and it was very secure.  It was not risky at all for your health.  Like there was no way somebody could get hurt.  And even parents were competing with us, so it was very fun.  If we did that, I would definitely try it.

**MODERATOR 1:**  Awesome idea.  Any thoughts on that obstacle course, circuit, that kind of thing?  Cool that the adults did it as well.  That’s fun.  On that note, Moderator 3 brought up parents involved.  Could you all tell me a favorite activity, if you have one, that you can do or would do with a parent or with a guardian or with a teacher, anything like that . . . or Maya, sorry . . .

**MAYA:**  We could maybe like go to a park or something or like water park or something.  Or we could like, I don’t know, go to, like my family likes to be going to the park or go bowling or skating.  But like I don’t know about your guy’s family.  But we like to choose something to eat out or something if we deserve it, if like we deserved . . . and we listen or anything like that.  Like they always tell us like we can go to the park, water park, splash pad, Jumpin’ Joeys, Urban Air skating rink or bowling, like family get-togethers, like family reunions.  Like I’m having a family reunion coming up soon anyways.

**MODERATOR 1:**  Awesome ideas, Maya.  That was like 15 ideas in 30 seconds.  I loved it.  We will, we’ll jot all those down.  Leah or Taylor or Casey or Morgan, other thoughts on the parent question?  Leah.  How about you Leah, or Taylor?

**GIRL:** So . . .

**MODERATOR 1:**  I couldn’t tell.  I think both of you were trying to say something, I think.  Taylor, you go first.  Then we’ll come back to you, Leah.

**TAYLOR:**  Okay, so I don’t know if Leah or Maya or if Morgan and them has any done, has done this before with their family.  I like to go laser tag.  That . . .

**LEAH:**  I've been laser tagged.

**MODERATOR 1:**  Great idea, Taylor.  Leah, what were you going to say?

**MODERATOR 3:**  I like that.

**LEAH:**  So I have done laser tag.  It is really fun.  But there was this one time I went to this theater in Tennessee.  And it was a 3D theater, so they would have a screen, and you would put these glasses on, and you would be in this chair.  And my dad, of course, had to pick the clown one.  It was scary.  So it was like you were on a rollercoaster, and your chair would move back and forth or front and back, and you would be strapped in.  It was really fun.  And it was like 3D glasses on and everything.  It looked 3D.

**MODERATOR 1:**  Awesome.  Casey, Morgan, anything you’d like to add about familyrelated physical activity?

**CASEY:**  No.

**MODERATOR 1:**  All right.  Moderator 2, Moderator 3, anything else before we wrap up, any questions, thoughts, ideas?

**MODERATOR 2:**  I just have one thing.  I think Leah might like this idea . . . competition.  Would you ever want to do like a tournament-style play of like dodgeball or capture the flag?  Is that something that would interest you?

**LEAH:**  Yes, very much.

**MODERATOR 2:**  That’s what I thought.  Maybe Taylor, I know you like dodgeball.

**TAYLOR:**  It’s Taylor.

**MODERATOR 2:**  Sorry, Taylor.

**TAYLOR:**  Do I look like my mom?

**MODERATOR 1:**  No, the name . . .

**MODERATOR 3:**  It's the name on the screen.

**TAYLOR:**  Yeah, I love dodgeball.  You know, I might get out, but, you know, at least I play, and, you know, at least I’m active.

**MODERATOR 1:**  Yeah.  Okay.  Moderator 3, anything?  Good?  Cool.  So everybody, awesome job tonight.  I so appreciate that.  We have, I don’t know, what do we have there, 100 ideas or so?  So great stuff.  We’re going to put together an easy-to-fill-out survey that just asks how much you like different ideas like on a one-to-ten scale, with one being you don’t like it at all, it’s a horrible idea, and ten being a tournament-style dodgeball sounds awesome, and so does an obstacle course.  So you’ll rate those really quickly.  It will be either on a phone or on a laptop.  Moderator 2 will send out the survey link when, Moderator 2, probably?

**MODERATOR 2:**  Probably tomorrow night if we get it . . .

**MODERATOR 1:**  Cool.  Either tomorrow night or, yeah, the following morning.  But that will have all the ideas from both the kids' session and the adult session on it.  We’ve got the survey half done, because we have the adult ideas on there.  And then we’ll send out the kids’ ideas too.  And you guys said some awesome stuff tonight, so I just so appreciate your help.  We’ll send out the gift cards, again, tonight or tomorrow, Moderator 2?

**MODERATOR 2:**  You’ll get them in like ten minutes.

**MODERATOR 1:**  Cool.  So the e-gift cards will be coming, Amazon, $40.  Or Riley will be sending, Moderator 2 is going to connect with you and your mom, Riley, about sending the Visa gift card.  And then could everyone potentially be available next Thursday again, same time, 7:00 to 8:00?

**GIRL:**  Yeah.

**MODERATOR 1:**  Beautiful.  Okay.  So we’ll send reminder text messages.  Next Thursday will be session number three out of five.  And then we’re probably going to take one week off after that, so next Thursday we’ll be on, and then we’ll have a week off.  We’ll message you, same time, same place.  I just appreciate you all so much.  Those were awesome ideas.  Thanks for coming, and we’ll see you again next week.

**MODERATOR 3:**  Bye, guys.  Thank you . . .

Indiana University Sport Management

Hoosier Sport

Adult Design Session 3

**JASMINE:** I’ve never played it.  I’ve never . . . I've never heard of it, so I don’t know anything about that.  But softball, basketball was my two big things.

**MODERATOR 1:**  Oh, awesome, softball and basketball.  Cool.  And, yeah, no worries about pickleball.  It’s relatively new.  I don’t even, I’m not sure how many of the kids would know about it or not.  But it’s just another random sport out there.  How about Kim or Monica, favorite sport to play and/or watch?

**KIM:**  I guess favorite to play would be soccer, and we also enjoy watching soccer.  Pickleball, I know . . . built a brand-new pickleball court or, pickleball court at their park.  So it’s a growing sport for sure.

**MODERATOR 1:**  Oh, nice, and in the county as well, that’s cool.  Soccer, can you tell me more about that, what’s the, what sparked that or what you guys like to, like I don’t know if there’s certain leagues you watch or participate or that kind of stuff?

**KIM:**  We like to watch the U.S. Women’s soccer, and then we watch a lot of the college soccer.  We watch the men’s soccer.  We usually watch pretty much all the soccer that’s on . . .

**MODERATOR 1:**  Oh, awesome.

**KIM:**  . . . subscription that we pay for is soccer.

**MODERATOR 1:**  Cool.  I like it.  That was heartbreaking.  Did you guys watch the World Cup game that went to the shootout?

**KIM:**  We don’t talk about that.

**MODERATOR 1:**  Yeah, that, oh, man, that ball.  Dang.  I won’t bring it up again today, probably.  But, dang, that was tough at 6:00 in the morning or whatever it was here.  Monica, how about you, favorite sport to play or watch or both?

**MONICA:**  Well, I played softball in school.  That was many, many years ago.  And my kids play soccer and basketball, so I really like watching like the high school level.  Well, my daughter is a manager at Purdue now, so I guess I kind of watch them too.  But pickleball is something that my daughter, one of my daughters has learned how to play, because it’s really big in Lafayette.  And so she’s, I guess, going to teach me how to play on the new one in Bloomfield.  And I know I’m kind of scared, but my family is very sports-oriented.  I kind of follow my kids, and I’m not as sports-oriented as they are.

**MODERATOR 1:**  Cool.  Thanks for sharing that, Monica.  So reason we’re asking about sports, now to get a little bit more specific with kids, so I’m interested in the three of yours opinion for if you were to pick, so we don’t have a number set in stone of how many sports we can, you know, teach and kind of build around, and we want them to be a bunch of things.

We want the kids to like them, so they need to be fun.  The equipment needs need to be there, so ideally less equipment intensive and more like, of course, we're not going to choose hockey or swimming.  But we could play like floor hockey or something like that.  I’m a Canadian, so we grew up playing that.  But some sports would be not a great fit for within PE class, and some would.

So in, you guys know your kids best.  Both boys and girls, we want these to be inclusive.  We can teach them in a very adaptable way, like a small-sided soccer instead of just like big games, that kind of stuff.  If you had to pick three or four sports that come to mind to teach, to use to teach all these things, the leadership, teamwork, goalsetting, physical activity, all this stuff we’ve talked about the last few sessions, what are the three or four sports that come to mind for you that would be a good fit in WRV PE?  Whoever wants to go first.

**JASMINE:**  I would say softball or baseball, whatever it is, and basketball and like dodgeball or something like that.

**MODERATOR 1:**  Great point, Jasmine.  Yeah, so it doesn’t need, the softball, baseball, excellent, and then dodgeball.  Like it doesn’t need to be a . . .

**JASMINE:**  I mean . . .

**MODERATOR 1:**  . . . professional sport.

**JASMINE:**  I played, when I was in high school, I played, I don’t know if you know what four square is.  And I liked that a lot.  My daughter hasn’t played that, though.  But that could be something too . . .

**MODERATOR 1:**  Yeah, that's . . .

**JASMINE:**  . . . much more popular game with a lot of . . .

**MODERATOR 1:**  Sorry, Monica, after you.  While Monica is getting that figured out, hello, Avery.

**AVERY:**  Hi.

**MODERATOR 1:**  So just catching you up, all you’ve missed is we started talking about sports and what might be a good fit at WRV.  We’re looking for three or four sports.  I’ll come to you last.  Jasmine just was, just went.  We’re thinking of what sports might be the best fit within PE class to use as like a teaching tool to teach them some sports skills but also teach them about leadership and teamwork and goalsetting and physical activity and all that.  So I’ll come to you after Monica and Kim.  So Monica or Kim, whoever wants to go next, three or four sports you think would be a great fit at WRV PE this fall.

**KIM:**  For PE I think the kids really like dodgeball.  For, I like softball, but for PE, it may be hard, because there’s a lot more gear that goes with it.  But soccer is a good one.  Basketball is with it, because it just requires a net or a hoop.  Or maybe some sort of obstacle course, like the, oh, not quite the IRONMAN but, or a CrossFit type thing, running, jumping, doing all the different skills throughout the class.  I guess that’s technically not a sport, but *American Ninja Warrior* type thing.

**MODERATOR 1:**  Great ideas, Kim.  Yeah, and we could totally turn than into something, like obstacle course, yeah, including some CrossFit type stuff.  So like a strength-based one would be good too, so it’s not, it doesn’t always have to be all about running.  And funny that you guys bring up dodgeball too, because the kids in our kid sessions have definitely been bringing up dodgeball.  How, Monica, I think that was, I think we missed what you said, Monica, if you don’t mind saying yours again, three or four sports.

**MONICA:**  Oh, that’s fine.  I thought like I lost you for a second.  But the four square that she was bringing up a minute ago, I have a . . . at my work, and the kids really like it.  And then I was thinking volleyball and then, of course, the ninja warrior thing too because . . . active.

**MODERATOR 1:**  Cool, *American Ninja Warrior*, I like it, and volleyball.  What’s the, so I come from, being from Canada, they play men’s and women’s volleyball.  What’s the, in, at WRV, how would the boys respond to volleyball?  Would they be like, heck, yeah, or would they be like, no, that’s a girls’ sport or . . .

**AVERY:**  No, my son is like begging for them to do it.  Like he loves it.  He’s, like he’s even wanting to put one in town if he can.

**MODERATOR 1:**  Oh, awesome.  Yeah, I love volleyball.  Okay.  Cool.  And then how about you, Avery, so if you had to pick three or four sports that you think would be a good fit at WRV within PE class, what would those be?

**AVERY:**  Well, I went to Shakamak, and a good one for us was whiffleball instead of baseball or softball, because you could do it inside.  And we played a lot of whiffleball.  I would say volleyball too or something like badminton.

**MONICA:**  I thought of another one.  But kickball was always fun when I was a kid.  We played that.

**MODERATOR 1:**  Great ideas.  Okay.  That’s awesome.  I think we’re going to end up picking, Moderator 1, probably like two to four.  We might have like a four-week, a month set on one sport and another month guided by like, or we could have two within.  So we’ll see.  But awesome ideas.  Thanks for those.  Let’s go to some survey results.  So the, did anyone, any of you not fill out the survey?  And it’s okay if you didn’t.  Moderator 1 can send you the link to fill out after this session.  Did anyone not fill it out, the one about program ideas?

**MONICA:**  I believe I did not.

**MODERATOR 1:**  Cool.  Okay, so Moderator 1 will send you that.  It will just be a link.  It will probably take four minutes, maybe five minutes, maybe three minutes to fill out.  No rush right now, but if you could fill it out after tonight, that would be great, after our session.  And then, because I’ll like recalculate these results, and we can go over them in our off week next week, because we won’t be meeting next week.  But let me share my screen with you, and I think you’ll be excited by the results.  Okay, so let me blow it up a little.  Can everybody see that okay?

**AVERY:**  I can.

**MODERATOR 1:**  Okay, let’s make it a little bigger.  How about now, is that better, Avery?

**AVERY:**  Yeah, that’s better.  I can read it now.

**MODERATOR 1:**  Cool.  So basically we have program ideas on the left, the score, one out of ten, and this is an average, on the right.  And I put the green one, so it scored a nine or higher.  The yellowish one scored an eight.  And then I left anything under an eight as a seven-point-something.

And so these results are, it’s just one little tool for us, and we had like 12 people respond that were either WRV participants like you all or a couple child participants, IU students, and then a couple IU faculty members.  So that’s why, Kim, your results will still be good, because there’s a few people we got to track down still, including a few, probably a few more WRV students to fill it out, a couple more at least.

But anyways, long story short, some of these things are like actual program ideas, and then some are more like concepts or like rules to include in the program.  Positive peer mentoring was our highest scoring one, so that was cool to see, 9.67.  And that would be something like a program strategy or an intervention strategy that we could like tangibly, next session, when we meet in two weeks, we can, we might talk a little bit about it today, but we’ll be able to talk more about what the details of that could look like, so like how would we partner up the kids or something like that.

And then, so the next one that looked like a pretty tangible, so I guess these 9.5s here, these were other like kind of principle ones.  That’s the people teaching the program to pay attention, making the program accessible to everybody, so it’s not just for athletes.  It’s not just for the most skilled kids.  Fun, super important, not surprising to see that one at a 9.5.  And then having IU athletes come talk to WRV students, I feel like that could be like another program strategy of ours.  Like maybe every week, we get one or two athletes from, either males or females, a different sport, to come chat with the students about whatever the topic is.

And then some more like principles, be inclusive, encouraging students, encouraging teamwork.  Another one that looked like a program strategy would be the short physical activity breaks during the school day.  So that one scored a 9.14, so nice and high too.  Good sportsmanship, of course, important.  Open gym time we talked about a little bit last time.  That one scored really high as well.  And then peer mentoring from kids that are in a higher grade, so that was another one that could be a strategy.

And I don’t want to go through all of these.  That will get a little boring.  Let’s look at some of the lower ones.  The walkathon didn’t score well.  And this doesn’t mean these are like totally excluded, but the jump ropeathon, fundraisers, nonphysical activity clubs, I thought that was kind of funny to see chess club score low, which is not overly surprising.  Nutrition competition scored low.

So, yeah, any questions or thoughts on those before we chat about them some?  Anything about the results you’re curious or interested in?  And Moderator 1 included, because I don’t know if you’ve seen these ranked yet either.  Okay, cool.  So let’s talk about some of these program ideas.  Positive peer mentoring, when you hear that, what does that, what, how do you envision that?  Positive peer mentoring, how do we like make that work at WRV?  What are some ideas for how we could implement that?  Positive peer mentoring, what could that look like?

**JASMINE:**  I think like having a student, some kids do this, like have a student or a principal or something dress up and like be at the front door and be like, have a great day, or something like that or say a little quote or play a little game with them or something.  I know when my daughter went to elementary, the cop up there, I don’t remember which one it was, he would play like a game with her to kind of get her brain motivated.  Like what is today?  Even though it would be Monday or whatever, it would still get her thinking and like looking forward and positive or something like that.

**MODERATOR 1:**  Yeah, that’s some really positive role modeling.  Would you, did you ever hear of the kids getting involved in that, like the children doing it for each other or to, or like a kid was assigned to the front door or anything like that with kids?

**JASMINE:**  I never got told any more, you know.  I just know that she would come home and tell me that her and the cop had that, they played a game.  I’m sure he played it with every kid, honestly.  I don’t know.  You know, I just took her to school, dropped her off, and then she went.  But she’s like, oh, it was, like her made her day a lot better, she said.

**MODERATOR 1:**  Oh, that’s awesome.  Thanks for sharing that, Jasmine.  Everybody else, when you hear positive peer mentoring, what does that look like to you, and whether it’s from past experience or what you think it should look like at WRV?

**MONICA:**  I think that peer mentoring . . . the extra, like the kids helping each other out, maybe setting up a buddy system where an older student is kind of checking in on the younger students, especially somebody that’s first starting school as opposed that’s like in the fifth or sixth grade.  Because then that way, they know that they have somebody that’s older that is pepping them up, talking to them, telling them what the day is going to be like, or telling them what the school is going to be like or how the schedule is going to be, just kind of helping them walk through the day-to-day system.

**MODERATOR 1:**  That makes sense, Monica.  Avery.

**AVERY:**  Yeah . . . well, whenever I was in high school, I think I was in probably ninth or tenth grade.  We had a system set up where we went and we did pretty much that.  I went to the third grade, and I read to them.  And then as they were taking their quizzes, I helped the teacher as, and I walked through, and if I see any kids struggling, I would sit down and help them.  It was really nice.  I mean, I have a kid, not a kid anymore, but a guy that’s told me that he remembered me doing that.  And we’re in our 20s, and he still remembered me doing that when he was in 3^rd^ grade.

**MODERATOR 1:**  Wow, powerful.  That’s awesome.  Do you think, so tell me more about that, anybody, if that should be like one year up or going to the elementary schools.  I guess I’m interested in both sides of it, because like a sixth-grader, for example, or even an eighth-grader, whatever, they can mentor down, and they can also be mentored from someone older than them.  So tell me more about that, more stuff like those systems.

**JASMINE:**  I think it can be high school or middle school or, I mean, honestly, to help out, like that one girl said, just to help out new students or anything.  I mean, I think it can be any level.  Because kids are always coming in or changing schools or something different, so.

**AVERY:**  I always liked it when an older kid like helped me, and like a high school kid.  I always remembered that when I was young.  I don’t know why it mattered more than a kid just a year or two older than me, but that’s just what, that was my, what I took out of it.

**MODERATOR 1:**  Yeah, great point.  I think I remember the same, like two or three years older.  I was, I definitely remember those.  And whether it’s from elementary school or whatever, you just really look up to them for whatever reason.  So how about, and how about Kim and Monica, what do you think about that with like same year, one year up, going to a different school, the mentorship both ways, like getting from someone older than you, mentoring down?  What does that look like to you?

**MONICA:**  I think it just always is, well, elementary, I think the high school is kind of cool to come in because they can kind of see.  It might be nice to do something that’s like four years apart, so start in like fourth grade and an eighth-grader.  That way, they kind of get the same peers almost.  So when they go to high school, they’re a freshman and senior, to kind of grow together.  And they could have that as a bond that hopefully they stay together as their set peer group.

But one thing with the, when you’re coming into school, they could have like one of the teams, whether the soccer team, volleyball team, welcome all the kids to school on any one day, just to give high fives as the kids get off the bus.  That’s always kind of fun, because the kids look forward to seeing that, the basketball kids.  The girls, the boys, it doesn’t matter the sport.  But I know that the younger grades really enjoy that.

**KIM:**  And I think, sorry, just real quick, for like our kids, they would need to mentor, you know, some kinds to feel better, you know, at least a couple years younger than them too, or a year at least.  Let them be the mentor.

**MODERATOR 1:**  Yeah, I love that.  Great ideas.  And mentoring fourth to eighth, that would be incredibly cool if they took that to then high school, because I’m sure some of these relationships would be good, some of them would be neutral.  So tell me more about that.  What do you think, in your past experience or just in your general ideas for assigning or partnering mentors, what could be a good strategy for trying to get them partnered up?  And should it be male-male, female-female, and what, how would you go about trying to pair kids up?

**KIM:**  I would say you could leave it up to a questionnaire.  Would they feel more comfortable with a same-sex peer or an opposite?  Leave that kind of up to either side, whether if the younger or the older peer.  I would start with that, so they can decide which is, which they’re going to be more comfortable with.

**MODERATOR 1:**  Thanks, Kim.  How about everybody else, Avery, Jasmine, Monica, same thing about ideas for pairing them up?  It’s not going to be perfect, but how can we at least try to make it promising?

**AVERY:**  Yeah, I mean, just like she said, you could do like a little, quick survey for each one of them.  But if that’s impossible, I would say probably the same sex so that the younger ones aren’t intimidated and the older ones don’t feel like they could, I don’t want to say this, but get in trouble or something.  I don’t know.  That’s a touchy subject.

**MONICA:**  Well, and the other thing too is in the questionnaire, you could have them answer questions about their likes, their dislikes, what their hobbies are, and you could kind of match them up to where they have like likely interests.  So they would have something to talk about, or they would, you know, they would mesh better than just matching them up based on age or whether they’re same sex or not.

**MODERATOR 1:**  Great ideas . . .

**WOMAN:**  . . .

**MODERATOR 1:**  . . . mentoring questionnaire.  That’s super good.  Avery?

**AVERY:**  I said that’s a good idea.

**MODERATOR 1:**  Yeah.  And it might even be good to like, like it can be tough if you get stuck with the wrong mentor, you know.  So it might be, there might be like a trial period or like a six month or something, or it might just, maybe it’s just for the year or for a semester.  And they could keep going, or they could try to switch like if it wasn’t going great.  But I love the questionnaire idea.  We could come up with some, like a few questions for like criteria of similar interests or what you’re looking for in a mentor or a mentee.

So awesome idea.  Peer mentoring questionnaire, Moderator 1, sound good?  Cool.  All right, we’re on it.  Yeah, that one scored highest, so that one is super important.  And that fits a little bit with, we had a couple items that were peer mentoring from kids that are in the same grade or in a higher grade.  And the higher grade, if I’m not mistaken, scored quite a bit higher.  Yeah, it was like in the high eights, where I was getting peer mentoring from the same grade is, was in the 8.0.  So, yeah, those results match what everybody is saying.  That’s awesome.  Anything else on that, Moderator 1, before moving on, peer mentoring?

**MODERATOR 2:**  No, I think that’s good.  I think we have good ideas.

**MODERATOR 1:**  Cool.  Okay, so next one, or actually, I do have one more question on that, actually, the topics.  So when you think of kids getting either peer mentored or doing peer mentoring, what do you think are a few of the most important things to do that on?  Avery, you mentioned reading as one.  That or what else, what are some of the, a few of the most critical topics?

**MONICA:**  Well, tutoring would be a good one too.  I know the reading to them but also showing, like being able to help with different classes or subjects that they’re having difficulties with.  And relationship building, I mean, I don’t know that you can really ask a kid to teach somebody how to have a relationship.  But if you’re overseeing it, then you’re helping both kids learn how to develop relationships in a healthy way.  And I think that’s really important because so many kids stay on their phones now, they don’t really know how to talk.  So I think that would be a huge thing for a mentor.

**AVERY:**  Oh, yeah.  Maybe like in that questionnaire, put something like the older mentor has overcome in their life, and then a child, if they’re going through the same thing or struggling with it, maybe match them up.

**MODERATOR 1:**  Great ideas.  Jasmine, Kim, important topics for mentoring between these grades?

**JASMINE:**  I think they kind of covered it.  Otherwise, I don’t really have anything else.

**MODERATOR 1:**  Cool.  All right, good.  Awesome, peer mentoring covered, check.  So IU athletes coming to talk to WRV students, that was the next highest like tangible program or intervention strategy that we could use.  And what, I wanted to ask a similar question with that.  Topics, so if you envision IU athletes, whether they’re boys, girls, whatever, any different sport, from rowing to basketball to volleyball, softball, what are the best topics you would like to see them coming to WRV to discuss?

**KIM:**  What it took to get there and what their day looks like, how much time they actually take studying, to kind of show how important that academics are.  Especially in college, you can be the greatest sports player there is, but if your academics aren’t there, most schools will pass you up.  To show them that it is important, that their grades do matter.

**MODERATOR 1:**  Great points, Kim.  What else, Avery, Jasmine, Monica, what would you like to, and you could reiterate the same thing if you feel the same or other ideas too that they, you’d like to see the topic of at WRV?

**MONICA:**  It may sound silly, but follow-through.  Yes, the grades are important.  Yes, the being good at sports and all of that is important.  But it has to be that person’s follow-through, because they have to, kids, younger kids, they have all these dreams about being on stage or being the biggest sports star that there is.  But having somebody from the college that comes in and says, you know, it takes a lot of work, and you actually have to be dedicated and devoted to it.  And kind of like what she was saying about the grades and everything, but it’s more than that.

You know, I can’t get my son up to go to practice every day.  He has to be willing to do it.  I can force him to play basketball, but if I force him to play basketball, then he doesn’t have the love of the sport.  And so that’s a big thing that kids miss when they’re younger, is they see all these people on TV.  And then if they have somebody from the school actually coming in and talking to them about real issues, about the follow-through and being dedicated to it, I think that would make an impact.

**MODERATOR 1:**  Thanks, Monica.  Avery or Jasmine, other things you’d like to see them come speak about?

**AVERY:**  I think they covered it.

**MODERATOR 1:**  Cool . . .

**JASMINE:**  I agree with Kim.  They need somebody to like show them and like how they got there and not just think that, oh, if I just go to practice, it’s a piece of cake now.  But it’s not.  You know what I mean?

**MODERATOR 1:**  Mm-hmm, yeah.  How about, interesting question, hopefully, on the flipside of that, so I’d hate for them to come to the school and act like they know it all.  So what would you, if we were preparing these students to come to WRV, what would you like to see them avoid or like not do or not talk about?  I don’t know if that’s a topic or just a behavior of theirs.  Yeah, what should they not do?

**MONICA:**  Brag.

**MODERATOR 1:**  Thanks, Monica.  Totally.  Humble, be humble when you come.  Anything else to avoid or not do?

**MONICA:**  Well, I think, and everybody should be included.  Not everybody is going to be the pro sport.  Not everybody is going to be the athletic, have the natural athletic ability.  So if they’re talking to everybody about it’s not just about athletics, it’s about what you can do with the education, so that way, the kid that is sitting there that can’t dribble and walk at the same time isn’t going to feel left out.

**MODERATOR 1:**  Really great point, Monica.  Yeah, it’s not just athletic talks and here’s how to be an athlete and that kind of stuff, because that will only appeal to a small number of them, I would think.  Okay.  Anything else on that one before we move on?  All right.  So short PA breaks or physical activity breaks during the school day and supervised open gym time in the morning, those two topics.  So let’s go to short physical activity breaks during the school day.  When you hear that, what, this would be a great question for . . . but Jasmine, you also work in the school, right?  And I'm not sure . . .

**JASMINE:**  Yes.

**MODERATOR 1:**  . . . about everybody else, but ideas for what you’d like to see that look like.  And that’s also where we’re incorporating all the, you four know better than I do how much screentime your kids have.  And we talked about that in the past.  So if you were to design the short physical activity breaks during the school day, what would you do?

**JASMINE:**  I would like do like an exercise maybe in the classroom with them to kind of get them a little motivated, go outside, take a little walk or something.  I mean, you don’t always have to play sports, or you don’t always have to play something, you know.  Or you can play like a game, like I said earlier, to kind of get their brains and stuff motivated in the mornings.  Because some of them, I know my daughter . . . she’s not awake most of the mornings.

**MODERATOR 1:**  Would it be fair to say these should happen especially in the morning or just in general throughout the entire day?

**JASMINE:**  I would say throughout the whole day.

**MODERATOR 1:**  All right, Avery, Kim, Monica, what would be, what would the short physical activity breaks look like to you, and knowing that, at least so far, the principal and superintendent have said they’ve been, they're really supportive of ideas for policy changes in the school?

**AVERY:**  Jumping jacks, I mean, maybe say your ABCs or something that you have memorized as you’re doing it.  I don’t know, just some 10-minute, maybe 15-minute, not very long, play a boardgame, like she said.

**MODERATOR 1:**  Thanks, Avery.  Kim?  Monica?

**MONICA:**  I think throughout the day would be good.  And like everybody is saying, it doesn’t have to be really long.  We have different things that we do with the kids that I work with, where even if it’s just five minutes.  And I’ll come up with ways of getting them to go and do stuff, like asking them to go to the room to get something just so that I know that they’re walking down the hallway and coming back, or asking them to go to the carport and pick something up for me.  Again, it’s a five-minute thing.  It changes the thought process in their head, and it keeps them moving, and then they’re just not sitting there staring off into space.

**MODERATOR 1:**  Thanks, Monica.  Kim, anything to add on that one?

**KIM:**  Not really, unless there was something maybe just not as much exercise space, but if everybody is doing their reading, and they’re reading off their Chromebooks, take the walk outside and at least do that activity outside.  Instead of being confined to a desk, they can at least be out in the grass or next to the playground.

**MODERATOR 1:**  Yeah, that makes sense.  And Kim, if you don’t mind, we can start with you on this one, since you’re rolling.  The, one of the next highest tangible program ideas that wasn’t just like a philosophy or something was the open gym time in the morning before school.  So if you were to design that, what would that look like?  Knowing that you, again, you all know best, whether everyone gets there at 7:15 or if it’s totally mixed or how we could make that most beneficial, that morning time from, I think it’s roughly 7:15 to 8:00 in the morning on some days.  How would you design that?  What are some important things?

**KIM:**  Well, with it being called open gym kind of gives the idea that it’s open for them to kind of do what they want as opposed to really structured.  Having a very loose structure that the basket, if you want to play basketball, we’ve got it set up over here.  If you want to read, finish extra homework, you can sit in the bleachers over here.  Just have more, have it broken down to different areas for the open gym so they get to decide what they want to do, not, you know, you’re here, you’re going to play basketball.  Because they may not really want to play basketball that morning.

**MODERATOR 1:**  Thanks, Kim.  So some loose structure but open, various options, various equipment ideas, but not just chaos.  Avery, Jasmine, Monica, other thoughts on how you would design that open time in the morning?

**AVERY:**  I’d say just like she said, just have, you know, the basketballs out and ready or the kickballs out, the bases.  When they get there, they can just grab what they want in their area and go ahead and start.

**MODERATOR 1:**  Thanks, Avery.  Jasmine, Monica, anything to add on that one?

**JASMINE:**  Just make sure like if they do do an open gym, like they’re not just sitting there playing on their phones.  You know what I mean?

**MODERATOR 1:**  Do you guys know if they ever have any like no phone time?  Like are they like, and is it up to the teacher, individual teacher to say you’re not allowed to use your phone during class, or is there any school policy right now that’s about their phone usage?  Do you guys know anything about those?

**MONICA:**  I think, from what I read today with one of the, they’re cracking down a little bit more on the phone time and not wanting them to be on them this much.  They got away from it at the high school level anyway, because my kids were always told to use their phones to look stuff up because of all the firewalls that they weren’t able to do on their iPads.  But we got notice today that it seems like they’re cracking down a little bit more on that.

**MODERATOR 1:**  Thanks, Monica.  And Monica, anything to add on the morning gym time, what you think would be the most beneficial?

**MONICA:**  Well, I think that the loose structure was, is a good idea.  And it’s kind of the *Catch 22* because then you have to be careful that it’s not real cliquish to where you have the, again, it’s not just about the sports, but then you also don’t want the basketball players all playing basketball and the other kids feeling like they’re not welcome to do the other stuff.  And so just making sure that the, I believe in loose structure, but I also believe in making people feel welcome.

**MODERATOR 1:**  Yeah, excellent point.  So that one will need to be really thoughtfully put together so it doesn’t turn out that way.  Structure, how popular?  So y'all four of you, one to ten, how popular do you think that before-school gym time would be?  Jasmine, one to ten?  Ten is super popular, all the students would want to go.  One is everyone would hate it.

**JASMINE:**  I would say at least a nine.

**MODERATOR 1:**  How about Monica, what do you think?

**MONICA:**  I think it would be between a seven and an eight.

**MODERATOR 1:**  Kim?

**KIM:**  I think it would be fairly popular.  I’d say probably about a seven.

**MODERATOR 1:**  Cool.  And how about you, Avery?

**AVERY:**  I think it would be about an eight.

**MODERATOR 1:**  Okay, cool.  So we think we’d have quite a bit of participation.  That’s awesome.  Let’s see.  So that’s the short physical activity breaks.  Moderator 1, do you have any other questions?

**MODERATOR 2:**  I was wondering if you guys would think handball would be popular, because that’s really cheap to make.

**KIM:**  It was popular when I was in school.  We also did tetherball, but I was, I grew up in a much warmer climate, where we were outside a lot more than you are here yearround.

**MODERATOR 2:**  All right, that’s good input.  Thank you.

**MONICA:**  I think that once they learned how to do it, they’d probably look at you funny to being with, because kind of like the pickleball, not everybody knows what it is.  And, but I think once you got them to see what it was, I think they would enjoy it.

**MODERATOR 2:**  Yeah, I agree, because I played it a lot as a kid, and it was like my favorite thing to do at recess.  And I was thinking if we, it’s easy to make.  You only need a piece of wood and a ball, and you have so much space at WRV that I thought maybe that would be a good idea.  But thank you, and I appreciate that.

**MODERATOR 1:**  Awesome.  And, hi, Jennifer.  Thanks for coming.  We’re just wrapping up.  I think we’ve got, I have one last question.  So I’m going to share my screen again with those survey results, and we’re going to look at some of the low ones just for a moment.  Okay, so I’ll go through these real quick.  Some of the ones that were in the sevens were engaging and, and I’m going to ask if there’s any of these that you feel really strongly about that we should definitely consider, if you’re like, what, how did that score low, what’s going on?  Because, again, it’s just like 12 responses, so it’s not that all of these are going to get cut, because some of these might be great ideas.

So I’ll read through the list, in case you can’t totally see it, of these ones in white, and then I’m going to ask if there’s any that stand out to you that you really think we should like not forget about.  So we have engaging in creative activities, like playing Legos, new activities kids don’t normally do, like chess, debate, science activities, incorporating nutrition, like tasting fruit cups or other healthy snacks, obstacle courses, IU students helping kids set goals, teachers helping kids set goals, nutrition-based competitions, treasure hunt-style competitions, parents helping kids set goals.

So all three of our adults helping kids set goals were relatively low, and that could just be that goalsetting was a little lower than some of the other more exciting things.  WRV student ambassadors identified to serve as leaders, and we might call those like WRV champions, something like that.  Fundraisers, jump ropeathon, nonphysical activity clubs, and then walkathon.  So anything there that jumps out at you as like, hey, that, we really need to make sure we still consider that?  Thoughts?

**AVERY:**  I thought fundraisers should probably be higher than that.  I know it doesn’t sound like much fun, but anything to help WRV, you know.  We . . .

**MODERATOR 1:**  Yeah, totally.  Yeah, not tons, it could be fun, though.  We could make it fun.  And, yeah, it is super important, money.  So Kim, Monica, Jasmine, anything else stand out there or any thoughts on the goalsetting stuff, any of those?

**JASMINE:**  I think like the trying the new foods or the fruit cups or something, I mean, I’m always game to try new stuff, new foods or whatever.  I know most kids aren’t, but I know like, I have my daughter.  I have two kids.  I’m working on my third one.  So I have them try everything.  So I think that would kind of be something cool.

**MODERATOR 1:**  Yeah, cool.  Thanks, Jasmine.  Kim, Monica, anything jump out from that list?

**KIM:**  I don’t know if it, I like the scavenger hunt thing.  And, I mean, it doesn’t necessarily even have to be a scavenger hunt.  But if they are partaking in the beforeschool open gym, have a checkoff that every week, your goal is to do five different things, not every day do basketball.  And have it checked off, and then at the end of the week, have a small prize, whether it’s something extra at lunch or a small, something of a snack bag type thing.  I like those.

**MODERATOR 1:**  Thanks, Kim, and cool ideas to make that fun and incorporate it into open gym.  Monica?

**MONICA:**  Well, I agree with everybody.  And to me, the obstacle course, again, we talked about the warrior type thing, and not be like a regular obstacle course but just something for agility and making it fun.  And I remember the obstacle courses when I was in school, and again that was many, many years ago, but they were fun.  And the way they make them now is even more intense, and I think it would get more people involved.

**MODERATOR 1:**  Yeah, I think you’re right.  Thanks, Monica.

**MONICA:**  I wanted to say the treasure hunt too, but I might be a little biased.

**MODERATOR 1:**  Yeah, okay, definitely.  Well, thanks for sharing, everybody.  Anything that was in that white section we are not just getting rid of, but then we also are at the point now where when we meet again in two weeks, we're, we want to really operationalize or like set out the details of the handful of most important things.

So we’ll, Moderator 1 and I will connect with everybody and the team over next week.  And then we’ll have, we have two sessions left, and we’re going to really try to solidify what this first Hoosier Sport program at WRV is going to be with more details, like the schedule and times and what things to include, what things we need to approach the principal and the superintendent about, if it’s something that requires like policy change or like teachers to be onboard or something like that.  And pretty much everything will, because we’ll also need like background checks on anybody coming from IU, all that stuff.

So I just appreciate all your efforts.  They’re totally awesome.  These ideas are so good.  I’m glad we have it recorded.  And again, if I haven’t said this to anybody, it’s all de-identified and just making sure we get all these ideas down to paper and don’t forget them, because my notes are not big enough.  How about you, Moderator 1, anything else before we go?

**MODERATOR 2:**  No, I think everything is great.  Thank you.

**MODERATOR 1:**  Awesome.  Well, thanks everybody.  Moderator 1 will send you a Visa or Amazon e-gift cards or the physical Visa card.  And then we’ll meet, can anyone not do Monday the 28^th^?

**MONICA:**  I can.

**MODERATOR 1:**  Did you say can or can’t?

**MONICA:**  I can.

**MODERATOR 1:**  Oh, awesome.

**MONICA:**  Able to.

**MODERATOR 1:**  Perfect.  Okay, great.  Well, we’ll shoot for that as our next session, 7:00 p.m., Monday the 28^th^.  We’ll be off next week.  And, yeah, thanks so much for coming.  Everybody have a great night.  And we meet with the kids on Thursday at 7:00 for their third session.  So thanks again.

Indiana University Sport Management

Hoosier Sport

Child Design Session 3

**TAYLOR:** Yeah.

**MODERATOR 1:**  Okay.  Cool.  So

[Simultaneous discussion]

**TAYLOR:**  No, I’m not . . .

**GIRL:**  . . .

**MODERATOR 1:**  Oh, and, Taylor, it might.  Sometimes if it’s a little loud it might mute your screen.  But feel free to unmute at any time.

**TAYLOR:**  . . .

**MODERATOR 1:**  Okay.

**TAYLOR:**  There.

**MODERATOR 1:**  So let me show my screen real quick.

**TAYLOR:**  No.

**MODERATOR 1:**  And, actually, wait.  Back up.

**TAYLOR:**  I wasn’t.

**MODERATOR 1:**  So first thing today, let’s talk about sports.  So I’m interested in your favorite sport to watch and your favorite sport to play.  And we’ll just go around the screen.  Leah, do you mind going first?

**LEAH:**  Yeah, sure.  So my favorite sport to play is softball.  My favorite sport to watch is football.

**MODERATOR 1:**  Oh, cool.  Okay, softball, football, awesome.  Then let’s go to Katelyn, how about you?  Not Katelyn, Taylor.

**TAYLOR:**  My favorite sport to play is softball.  And what I really like to watch is soccer.

**MODERATOR 1:**  Oh, nice.  What do you like about watching soccer?

**TAYLOR:**  It’s just I like different players, seeing them interact and like whenever they win.

**MODERATOR 1:**  Cool.  I like it.  And, Leah, how about you, where do you, what do you like about playing softball?

**LEAH:**  I like batting and catching popups the most.

**MODERATOR 1:**  Oh, nice.  That’s super fun.  Okay.  Casey and Morgan, how about you, same question, favorite sport to play and favorite sport to watch?

**CASEY:**  My favorite sport to play is softball, and I like watching soccer, because I don’t know.

**MORGAN:**  I like playing tennis, and my, and I like to watch soccer.

**MODERATOR 1:**  Cool.  Can you two tell me about what you like about watching soccer?

**CASEY:**  I don’t know.

**MORGAN:**  I like watching the women’s girls play.

**MODERATOR 1:**  Oh, yeah, like the women’s national team or the women’s anybody team?

**MORGAN:**  Mm-hmm, just like the women’s U.S. team.

**MODERATOR 1:**  Awesome.  Yeah, they’re super good.  Did you watch the World Cup?

**MORGAN:**  No, it was super, it was like 3:00 a.m., because it was like, it was in Australia, so the time was different.

**MODERATOR 1:**  Yeah, it was tough.  You had to get up really early.

**MODERATOR 1:**  Welcome, Moderator 3.  How’s it going?

**MODERATOR 3:**  Sorry, my full screen was blocked, so I, it like, it got frozen, so I had to turn off the computer and go again.  So sorry about that.

**MODERATOR 1:**  Hey, no worries.  We’re just going around the room.  And we’ll go to Moderator 2 next, and then we’ll come to you with your favorite sport to play and your favorite sport to watch.

**MODERATOR 2:**  Okay.  My favorite sport to play is, obviously, play water polo.  But my favorite sport to watch is probably volleyball, I think beach volleyball in particular.

**MODERATOR 1:**  Oh, beach volleyball, super fun, especially Olympic time.  Cool.  And how about you, Moderator 3?

**MODERATOR 3:**  For . . . track and field, the long jump, specifically, and triple jump maybe.  But to watch, I love basketball when it’s like a high-level basketball, and everybody is like screaming and, you know, it’s more intense.  Yeah, that one.

**MODERATOR 1:**  Nice.  What do you like about long jump?

**MODERATOR 3:**  I just like that little flying through space like.  It’s very fun how you can incorporate speed and control it to take off.  That is very hard to do, and I like challenges, so I guess that’s it.

**MODERATOR 1:**  Cool.  Flying through the air, that’s awesome.  Maya, how about you, if you’re there, favorite sport to play, favorite sport to watch?

**MAYA:**  I like to watch football and play volleyball.

**MODERATOR 1:**  Oh, nice.  Okay.  So we’ve got two football watchers.  Awesome.  What do you like about playing volleyball?

**MAYA:**  I don’t know.  I’ve just done it for a really long time.

**MODERATOR 1:**  Cool.  That makes sense.

**MAYA:**  And I . . . and I, so like the physical for the games today . . .

**MODERATOR 1:**  Yeah, volleyball is tons of fun.  So the reason I, we started with that is because we need, as a group, to figure out two to four, maybe three or four sports to design this first program around.  So the first eight weeks we come to WRB, we were thinking it might be something like four weeks on one sport and four weeks on another sport, maybe something like that.

So we wanted to brainstorm with you five what the best sports that sixth grade students might be interested in, and we want to, a couple things.  We’d want it to be fun, inclusive, be able to either have the equipment or purchase the equipment.  So we wouldn’t do something like ice hockey, because like we’d need an ice rink.  But we could do all sorts of stuff, all sorts of other ones.  I don’t want to put any ideas in your head.  So let’s go around the room one more time.  And if you had to pick three or four sports that you think would be the best fit at WRB this coming semester, what would they be?  And, Leah, do you want to go first again?

**LEAH:**  Sure.  Golf, softball, volleyball, and basketball.

**MODERATOR 1:**  Cool.  Can you tell me a little bit more about what made you choose even one of those or some of those?

**LEAH:**  So they just started golf, and I wanted to do golf, and I thought that was cool.  So that’s why I said that one.  Softball, I did.  It was pretty fun.  And then basketball, I’ve done it.  I did, I’ve done bitty ball.  And then volleyball, I just like to watch sometimes.

**MODERATOR 1:**  Awesome.  Very decisive.  Good job.  Who wants to go next?  After you, Taylor, three or four, even two, three, or four sports you think would be a good fit this coming semester.

**TAYLOR:**  I was thinking maybe like football, maybe some softball, you know, volleyball, and I would say golf.

**MODERATOR 1:**  Golf.  Cool.  Can you tell me about football?  What made you choose that first?

**TAYLOR:**  Well, our school really doesn’t, like they really don’t have much like sports that we can play.  It’s really mostly volleyball.  They just started golf, so golf, softball.  But you have to be on the school team.  You can’t be like on a community team.  You know, you have to be on the school team.  So why I choose football is because I think we don’t have, we do not have a football team at our school, so maybe like a football team.

**MODERATOR 1:**  Cool.  Yeah, great ideas.  Thanks for that background info.  And remember, all these questions, all night, there’s no right or wrong answers.  So we’re just looking for your opinions.  So thanks for sharing that, Taylor.  How about Casey or Morgan, whoever wants to go first, three to four sports you think would be a good fit at WRB this year?

**MORGAN:**  Soccer, basketball, track, and I want to, them to bring tennis to like WRB . . .

**CASEY:**  . . .

**MORGAN:**  . . . like junior high tennis.

**CASEY:**  . . .

**MODERATOR 1:**  Awesome.  So would you be willing to share what made you choose soccer, track, tennis?

**CASEY:**  Because I play, I want to do track, I’m going to do track this year.  Soccer, I’ve always done soccer.  And then I do tennis lessons, and it’s really fun.  I want other kids to like want to try it too . . .

**MODERATOR 1:**  I love that.

**CASEY:**  . . . want other kids to try.

**MODERATOR 1:**  Yeah, great ideas.  And those could be really inclusive sports.  When you think about track, what type of like events are you thinking?  Are you thinking about like jumping or sprinting or like throwing events?  Is there anything you’d think would be a, like most kids would enjoy learning?

**CASEY:**  I think sprinting would be probably the, or the hurdles jumps.

**MODERATOR 1:**  Cool.  Random side note, I used to teach at a speed school, which was all about teaching kids proper sprint form.  So that’s super fun.  I love that idea.  Then how about, tell me about soccer.  How do you think that would be received both by, I’m, we’re looking for something that both boys and girls would totally be into, do you think there’s any sport?  Let me see.  Hold on.  Let me rephrase that.  I’m making it too complicated.  Soccer and tennis, do you think they would be well received by both boys and girls?

**CASEY:**  Yeah.

**MORGAN:**  Yeah.

**MODERATOR 1:**  Okay.  Awesome.  How about your sister, three or four sports?

**MORGAN:**  Soccer, basketball, and track.

**MODERATOR 1:**  Cool.  And how about with track, what do you think would be the coolest events to teach, the most well received events?

**MORGAN:**  Jumping.

**MODERATOR 1:**  Jumping.  Cool.  High jump, long jump, or both, or triple jump, or all the jumping?

**MORGAN:**  Mm-hmm.

**MODERATOR 1:**  All?  Cool.  Okay.  Great ideas.  Okay.  And then, Maya, how about you, three or four sports you think would be well received by students a little bit younger than you?

**MAYA:**  Kickball, soccer, basketball, and softball.

**MODERATOR 1:**  What was the first one you said, did you say kickball?

**MAYA:**  Yeah.

**MODERATOR 1:**  Oh, cool.  Tell me about that.  Have you, yeah, what made you choose kickball?

**MAYA:**  Didn’t, like didn’t you ask what sports would be best for like people younger than me?

**MODERATOR 1:**  Yeah.

**MAYA:**  Oh, okay.  Then the kickball I thought because like, you know, it’s not like that active.  It’s like kind of simple.  You just like get the ball rolled to you and you kick it and run to a base.  And like you don’t have to like fully active yourself for the game.  It . . .

**MODERATOR 1:**  Yeah, I like that.  Great idea.  No, you wouldn’t have to be running all the time.  You know, like some sports you’d be running more than others, and that’s a good one.  Mm-hmm, I’ve got Corgis running around.  Okay.  Good.  So we’ve got lots of school sports.  Anything else on that, Moderator 2, Moderator 3, on sport ideas, sport questions?

**MODERATOR 2:**  I was going to say like field hockey and lacrosse, but those are not inclusive for both genders, but they give the opportunity for both boys and girls to play some stick sport.

**MODERATOR 1:**  Yeah, that’s cool and pretty minimal outside getting the, purchasing the sticks.  Moderator 3, anything else on sport ideas or sport questions?

**MODERATOR 3:**  I was going to offer track, but it was already offered, because I’m biased on that one.  And then I was thinking actually about tennis, but I don’t know if we have a facility for that.  Maybe pickleball can be substituted or . . .

**MODERATOR 1:**  Yeah, pickleball.  Yeah, tennis.  I’m guessing, so we’d probably need to do it in the gym, but, yeah, awesome.  Okay.  Great ideas.  We’ve got softball, volleyball, basketball, football, golf, track, tennis, kickball, field hockey, lacrosse, all sorts of good stuff.  So we’ll work with you guys and some adults over the next like six weeks or so on narrowing down which ones.  Then let’s move onto, let me show you some survey results that are cool in a simple way.  Okay.  Can you guys see the screen okay with, is it big enough?

**GIRL:**  Yeah.

**MODERATOR 1:**  Okay.  Awesome.  So this was a survey we sent out to some of you and to some adults and to some IU students like Moderator 2 and Moderator 3.  And we got, we had people rate what they thought were the most important program ideas.  These green ones were the highest-scoring ones, and they’re above a nine.  And then in yellow, you don’t need to memorize all these, but in the yellow are kind of medium, they are in the eights.  And then the white ones down here are sevens.

So this doesn’t mean like we’re not going to include any of the white ones or that we’re only going to do these green ones up here.  It’s just like one tool to try to figure out what everybody thinks are the most important things.  So the highestscoring one we’re going to talk about first is positive peer mentoring.  So that is, we’re trying to figure out what that would look like.  So that would be, let me ask you all a question.  I’ve asked something similar in the past, but can you tell me a story or an example of somebody either your age or a little older than you being a positive peer mentor for you?

**TAYLOR:**  You want to make . . .

**MODERATOR 1:**  Leah, any ideas?  Can you think of someone either your age or a little older than you that had a like a positive experience with you that like positively influenced you, yeah?

**LEAH:**  Jennifer, she is so nice.  I, she will get me to read a book, or she will get me to go to the library.  She’ll get me to do a lot of good things.

**MODERATOR 1:**  Cool.  Is she a, is she your age?  Is she a little older than you?

**LEAH:**  Yeah, she’s my best friend.

**MODERATOR 1:**  Oh, that’s sweet.  How awesome.  Jennifer sounds great.  How about what, wait, let me ask you one more question about that, Leah.  So Jennifer, what is it that she does that like helps you listen to her or helps you want to like do those positive things?  What is it about her that makes you feel positive about it?

**LEAH:**  She has high energy, so she’ll really get me to do it, or she’ll keep begging me to do it.  I’ll be like, okay, fine.

**MODERATOR 1:**  Okay.  Cool.  Good energy, persistent.  How about Taylor or anyone else, another story or example of someone either your age or a little older?

**MAYA:**  My friend . . . she, like when we’re in gym or something, she like, every time I like mess up on something she doesn’t like, she’s one of the ones that doesn’t like judge me for it or like laugh.  Like she is like helpful.  Like she basically taught me how to play volleyball.  She first joined volleyball, and then she wanted me to join.  And I told her, I was like, I can’t do that.  I don’t know anything about it.  And then she taught me for like a week straight, and then now I’ve been doing volleyball for almost two years, I’d say.

**MODERATOR 1:**  Oh, awesome, Maya.  What is it, what would you say it is about her that like allows you to like hear her and take on her friendship, her mentorship?  What does she do to make that happen?

**MAYA:**  I don’t really know what you mean by that question.

**MODERATOR 1:**  Let me try to rephrase.  How would you describe her personality or how she acts to . . .

**MAYA:**  She’s kind, respectful, friendly, trustworthy.  It’s, she’s like someone I can trust for like almost everything.

**MODERATOR 1:**  Okay.  Cool.  Thanks for sharing that, Maya.  Super helpful.  Taylor, do you want to go next?

**TAYLOR:**  Sure.  Well, is it okay if I do myself?

**MODERATOR 1:**  Yeah, of course.

**TAYLOR:**  So I love, and, I mean, I love reading.  And I have all different sorts of books.  I really love reading and doing different crafts.  And if like somebody gives me like a paper and scissors and tells me to make something, I will try to make something work.  But I am like I really like doing stuff.  I do.  I like playing with my little sister.  I like, of course, reading and doing crafts.  But that stuff makes me energized, because I think of it like so much that it just makes me energize and wants me to do that, like do everything.

Like if I wanted to make like my own like study group, then, you know, I would be reading and that.  I’d kind of be crafty.  I know I wouldn’t like see my sister a whole lot, but, you know, that like if I wanted to make something, then I have the energize to do it, because I’m thinking of that and not just, oh, let’s not, you know, let’s not play with your sister.  Let’s not read, and let’s not do this.  Let’s just be boring.

**MODERATOR 1:**  That makes sense, Taylor.  Thanks for sharing that.  How old is your sister?

**TAYLOR:**  She’s going to be two in December.

**MODERATOR 1:**  Oh, she’s very young.  Oh, cool.  Can you tell me an example or us an example of some time you have in the past helped somebody to either read or to make them something or what that looked like?

**TAYLOR:**  Well, I really like making my parents crafts and my friends crafts.  I really love to.  I like giving my sister a whole bunch.  But mostly I kind of get people like energized to like make their own like art group or reading group or, so that, it’s not . . .

**MODERATOR 1:**  Perfect.  Thanks . . .

**TAYLOR:**  . . . just, you know, I’m not just, oh, let’s not go teach somebody how to read.  Or because half of the boys in our class don’t even want to read, and some of the girls, like half of our girls and half of our boys don’t even want to read.  But it’s, I really like to read, so I like to make my own groups with Leah and Jennifer.  And I’m actually in one of their groups right now.

**MODERATOR 1:**  Nice.  That’s cool.  Great example of being a positive peer mentor and in reading, such an important topic.  So that’s a really good one.  How about Casey and, oh, Leah, after you?

**LEAH:**  Okay.  So the group actually is the reading group.  So I’ve been trying to get people to practice reading with us so we can get points, and we can keep reading, we can get our reading level up and all that stuff.

**MODERATOR 1:**  So either of you, could you tell me about how you pick people to be a part of that, because one of the questions later is how should we best like pair people up, or what kind of strategies have you liked in the past to try to find a good connection?  Because, you know, you don’t jive with everybody.  I forgot what the boy’s name was that’s really loud.  But it’s important to try to pair you up best.  So how do you pick your, those people in the groups that you target and anything you’ve learned about pairing up people to work with?

**LEAH:**  So what we did is we put no craziness for the sign-in paper to be in the group.  We put no craziness.  And so if it’s a girly book, the girls will get to read it.  If it’s a boy, a more boy book, the boys can go read it.  And then if it’s a book that everyone would enjoy, we would all read it together.

**MODERATOR 1:**  No craziness, that is a good, foundational rule to join the club.  Casey and Morgan, how about you, could you tell me a story about either you mentoring somebody or somebody mentoring you that’s either your age or a little older, little younger?

**MORGAN:**  Casey, go first.

**CASEY:**  I don’t really know.  I’ve got to think about it.  Morgan, you go.

**MODERATOR 1:**  Yeah, no worries.  Morgan, after you.

**MORGAN:**  Probably my best friend, Darla.

**MODERATOR 1:**  And what does she do or how does she, you know, tell me a little about what she does and how she does it that works well?

**MORGAN:**  Oh, geez, she helps me with my homework so I . . . do it.

**MODERATOR 1:**  Awesome.  Okay.  And, oh, by the way, so if you guys ever don’t know an answer, you can always say just like pass or you’re not sure.  Like don’t feel like you have to answer every question.  So it’s totally okay that your sister wasn’t sure of an answer for that one.

Let’s talk a little bit more about partnering people up.  So all of you have examples from classes where you’ve had to work with people that you’ve either liked or not liked.  If we, when we start Hoosier Sport at WRB this fall, one of the things, based on this survey and other stuff, is that peer mentoring might be a cool thing to include in the program.

But we’re wondering what you five think is the best approach for partnering people up.  Because like on one hand, we could just do it randomly.  On the other hand, we could make sure boys are with boys, girls are with girls.  We could try to find people that like similar things.  So what are some ideas that you have?  And, Taylor, after you.  I’m muted telling you that you’re muted.

**TAYLOR:**  So the way our class does, we really don’t partner up mostly, which I really don’t like.  So we, so I like to be with my best friends.  I do.  So, and half the people in my class don’t really get along with me.  I don’t know about Leah.

**LEAH:**  I do get along with you.  Don’t worry, girl.

**TAYLOR:**  I mean, other people like . . .

**MODERATOR 1:**  Okay.  How about you, Leah, ideas for what would be the best way, since we’re coming into this, and we don’t know all of you?  We know you five, so we could figure out how to partner you up best.  But with kids we don’t know, what would be the best way to partner, to pair people up with mentors?

**LEAH:**  Maybe first they could talk to each other first and get to know each other first, before they do that, before they get partnered and see if they’re compatible.  If not, they can switch partners.

**MODERATOR 1:**  That is an awesome idea.  So there’s this thing called speed dating that doesn’t need to be about dating.  It can just be about meeting new people.  And you have like 60 seconds with somebody, and you talk about X, Y, and Z.  So that could be something.  Great idea.  Maya or Casey and Morgan, ideas?

**MAYA:**  Can you repeat the question?

**MODERATOR 1:**  The question is if we were going to try to partner people up or kind of set up some kind of mentorship system at WRB, what would be some good ideas for pairing people up?  How would you do it?

**MAYA:**  Put an even amount of girls and boys on the same team so it’s just not like girls versus boys.  Because like I don’t know.  I just have a thing about that.  I don’t like that part when they just like put girls on one team and boys on the other so like to do like a mixture.  And then at WRB they put like an even amount of people on a team, and I just don’t think that’s okay and . . .

**MODERATOR 1:**  That makes sense.  Let me ask you five real quick, a quick question.  Do you think boys should only work with boys, or if, and, or and girls should only work with girls, or do you think it’s fine to mix it up?  Casey, Morgan, what do you two think?

**CASEY:**  I think it’s good to mix it up, because sometimes if you’re just . . . like the boys will just mess around, and sometimes the girls would, if you’re just doing all the girls.  So you have like an equal amount, so you’re still like having fun, but you’re not like distracted.

**MODERATOR 1:**  Oh, good point.  Yeah, it might be a little too silly.  Taylor, how about you, just boys with boys and girls with girls or mix it up?

**TAYLOR:**  You know, I really like hanging out with some of the boys, I’m not going to lie.  Some of them are okay.  Some of them are meh.  And some of them are just get out of here.  But I think we should mix it up.

**MODERATOR 1:**  Okay.  Cool . . .

**TAYLOR:**  Because I don’t really like, because some of the girls in my or our class, they love, and, I mean, they love to mess around.  Like I know them, and I like ask them if they get their homework done, and they say that they don’t even get their homework done.

**MODERATOR 1:**  Yeah, that makes sense, Taylor.  Okay.  So I’m hearing mix it up.  Let’s move on from peer mentoring and go to a next topic.  So one of the other things that was ranked really high, that is like a really tangible thing we could implement, is having IU athletes come from the University and talk with WRB students.  So just like Moderator 2 being a water polo athlete at IU and Moderator 3 being a long-jump athlete at IU, they’re perfect examples, some people that like talk to you about whatever you want to talk about.

It could be in small groups.  It could be in large groups.  It could be on any topic.  So I’m interested from you five in topics you think would be cool to have IU students come talk about with you, with small groups or with your classes, either one.  Who wants to go first?

**TAYLOR:**  I guess I will.

**MODERATOR 1:**  Taylor, after you.  Cool topics, what do you think?

**TAYLOR:**  I think we should do it in a smaller group.  That way, you know, because some other kids, you know, might get lost and say what was that again, you know, and then they don’t know what it was.  And I think what we should talk about is like kind of our future kind of ahead of us, you know, if we really want to go to college or IU or, you know, to the State and learn stuff, or should we just like, you know, I don’t know, just, oh, I don’t really want to go to college.  But me, I really want to go to college.  I really want to learn stuff, because my dream job is to be a veterinarian.

**MODERATOR 1:**  Cool.  Thanks for sharing that, Taylor.  Yeah, they could have some really good insight into that stuff.  Leah, how about you since you’re there?  What would be some cool topics you’d like to hear from IU students coming to WRB?

**LEAH:**  So hold on.  What was the question again?

**MODERATOR 1:**  What do you think would be some cool topics to have these really awesome students come from IU to talk with you guys at school?  What do, what would you like some topics to be?

**LEAH:**  Yeah, I do like Taylor talk about our future.  But maybe talk about how to get to know each other better, maybe even our teachers.

**MODERATOR 1:**  Cool.  And when you say get to know each other better, do you mean like social skills, relationship-building stuff, that kind of stuff?

**LEAH:**  Yes.

**MODERATOR 1:**  Okay.  Awesome.  Maya, Casey, or Morgan, how about you?  What are some, what are a couple cool topics or even one cool topic you’d love to have the content be about?

**CASEY:**  I would like to like, if someone like plays a sport like them get to talk to someone that plays their sport at like IU so you can kind of learn from them.  Instead of just talking to anybody, just talk to like someone that plays that sport that you want to like learn about.

**MODERATOR 1:**  Great idea.  Thanks for sharing that.  And your sister?

**CASEY:**  Morgan?  She’s in the bathroom.

**MODERATOR 1:**  All right.  We’ll pass on her.  Maya, how about you, some topics that might be cool to hear about from some college students that are total rock stars?

**MAYA:**  Maybe some like helpful information about like to like . . . your goal in like the sport you’re playing and like for them to like communicate like on like what to do better to like help you later in life in the sport or something.

**MODERATOR 1:**  Great idea.  And how about, oh, let me ask Moderator 2 and Moderator 3 first, actually.  What do you think?  Is there anything you want to ask about like in terms of other topics that would be good or not good?  Because, of course, like college and life and everything is about way more than sports.  So I do love coming to talk about sport, but then there’s also other stuff too.  So what questions, what question do you two have about other topics or other ideas so we can, because we’ve got the best source right here, these five?

**MODERATOR 2:**  Yeah, so when you guys are getting like into the middle school age era and then you go onto high school, I know when I was in middle school they kept talking to me about like doing extra, like being an ambassador for the school, doing more for your resume for college.  Would you guys be open to learning how to build your skills and your resume going into high school at all, or is that too early for you guys yet?

**MODERATOR 1:**  After you, Leah.

**LEAH:**  . . .

**MODERATOR 1:**  Oh, I thought you had your hand up.  Sorry.

**LEAH:**  Oh . . .

**MODERATOR 1:**  So what do you guys think, resume building for getting into high school and college.  Are you, is that on your, are you paying attention to that yet, or is that a little too early?  Taylor?

**TAYLOR:**  I don’t really, since I’m in sixth grade, I mean, people in this might be like in seventh or eighth grade.  But I don’t really think that’s too early.  I think, you know, they should be at least thinking of whether they want to go to college or if they want to go to high school.  So, but I don’t think it’s too early.  I mean, I was like maybe ten or nine or ten whenever I thought I really wanted to go to college.

**MODERATOR 1:**  Thanks, Taylor.  That makes sense.  Maya or Casey, Morgan, what do you think about that?

**MAYA:**  I’ve always wanted to go to college.  Like even though that I’m not like a big school fan, I just, like my mom suggested, like if I do go to college, it will like help with my life career or like the, like my dream or like what my goal is to do in life.  She said if I don’t go to college, then I won’t get like a good degree or anything or like a good job.

**MODERATOR 1:**  Thanks, Maya.  Leah, you want to add anything to that or no?

**LEAH:**  No, I’m good.

**MODERATOR 1:**  No, okay.  Cool.  Moderator 3, any topics you’re interested in asking about?

**MODERATOR 3:**  Yeah, I was actually curious in the same line as Moderator 2 about the future but in a different aspect.  It was about, if the kids at this age start thinking about scholarships for sports.  Because I know most, I’ve been a volunteer coach here at IU for two years now, and I’ve noticed the kids that come here, sometimes they think they don’t have enough talent or whatever to do a varsity team, universities.

And what . . . kids think about sports at this age and if you have been thinking about scholarships.  Because I come from a different country so it, college was not a topic for me until I was in high school.  But I know for some kids that I’ve taught, that during elementary school parents start talking about that.  So I was just curious about that.

And maybe we can, if your goal is to start mastering a sport at this age, this is more or less the age I started doing sports, maybe we can pay a little bit more of attention.  Instead of just doing a health-focused project, like maybe we can start training a little, at least in what we have.  If we have a water polo coach or a track and field coach present, we can help you get that little extra attention for development.

**MODERATOR 1:**  What do we think, scholarships, sport-specific details?  Casey?  Morgan?

**MORGAN:**  No, not really.

**CASEY:**  I want to be, I want to go to art school, but I don’t really want to play sports in college very much.  Yeah, I don’t really want to play sports too much, because I want to be a cartoonist when I grow up, so . . .

**MODERATOR 1:**  Cool.  Thanks for sharing that.  Anybody else?  Leah?

**LEAH:**  I would like to go to college and high school, because I want to be an engineer.

**MODERATOR 1:**  Cool.  I love that.  So I’m hearing a little more like life goals, kind of career path type things.  That totally makes sense.  Taylor, after you.

**TAYLOR:**  So, yeah, so I was talking about being a veterinarian.  I, and I really love dogs.  I really love cats.  I mean, if you, I mean, if I see an animal, I get so excited.  But I also love working on cars.  My dad teaches me how to like change the tires and do the oil and do the batteries and stuff.

**MODERATOR 1:**  Awesome.  Thanks for sharing that, Taylor.  So . . . ready to move on from that, Moderator 2, Moderator 3?  So we like the idea, and we’ll narrow in on the topics.  We have tons of cool college students that I think would be willing, that would be able to get come speak to all of you.  It should be super fun.  And I, yeah, I think some big groups would be okay.  I think it would be cool if we could also get some small group chats going, because then more people get involved.

How about a quick question, and then we’ll probably finish up with one more open one.  I’m curious if you five think that peer mentoring would be better if it’s all within the same grade or if you think it would be better if older kids mentored younger students.  Which of those two, in general, would be a better idea?

**MAYA:**  I think if older kids would like mentor like younger students to get them like ready for what they’re going to go through when they get older.

**MODERATOR 1:**  Yeah, good point, Maya.  Taylor, Leah, Casey, Morgan, do you think same grade, or do you think the different age model would be better?

**LEAH:**  I . . . a different age model would be better, because like what the other person said, it would be like better to like teach them what they know so then you can kind of go off that and . . . the older kid would do more of the teaching than as what kids in the same grade might just know the same thing so they wouldn’t be learning anything new . . .

**MODERATOR 1:**  Yeah, good point, really good point.

**TAYLOR:**  I think older kids should, because I just think they should be ready for what’s to come and everything.

**MODERATOR 1:**  Yeah, that makes sense.  They’ve been through it kind of like the college students coming to WRB too.  They would have been through some of the challenges that you all have been through.  Okay.  So I’m hearing older and younger would be better than same-same.  Awesome.

How about, so I have one last question, and then we’ll see if Moderator 2 and Moderator 3 have anything else.  I’m curious about short physical activity breaks during the school day.  So that was an item that scored really high that might be something we could try to implement at your school.

So you all are most familiar with your, how your class time is spent, how much screen time you have, like when do you get super tired, all that kind of stuff.  If you were to be in charge, you were the principal of the school, how would you design short physical activity breaks during the day?  What would that look like to you?  Leah, after you.

**LEAH:**  So what it would look like to me is I would think maybe a 20-minute, 30-minute break.  Because we stay inside for probably 6 hours and 45 minutes a day, and we only go out for 15 minutes, like isn’t very long, 10, 15 minutes.  It isn’t very long.  So I would have it 20 to 30 minutes for exercise, or we could split that up and have 2 recesses, have both 15 minutes’ recess.

**MODERATOR 1:**  Thanks, Leah.  Great ideas.  And do you think those, if it was just one break, do you think it would be better in the morning or the afternoon?

**LEAH:**  I would think the afternoon.

**MODERATOR 1:**  Cool.  Okay.  Next, if you were the principal, Taylor, how would you design these breaks?

**TAYLOR:**  All right.  So if I was the principal, I would decide like, so like we, okay, so I don’t know if our class even earned it, but we haven’t been going outside really much like every day.  But, because like I don’t even know if our class can earn it.  I don’t really know.  But so we have been like stuck inside.  Doggies.  So we have been like stuck inside . . . they’re cute.  We have been like stuck inside, and like we like we’ve been doing like work after work, and, you know, we haven’t really got taken outside.  So I think maybe we should do like 20 minutes in the morning and then maybe like 10 minutes in the afternoon.

**MODERATOR 1:**  Perfect.  Thanks, Taylor.  Thanks for sharing that.  Yes, Casey, Morgan, if you were principal, how would you design these . . .

**CASEY:**  I would do like a not a break in the morning, because like you just got there.  You don’t really need to.  Like not too much, because you just like you just got up.  I mean, you were outside probably getting off the bus and stuff.  So you’re still outside.  But like in the middle of the day, I would say you probably need like not too long of a . . . but a shorter break, like a short break so you aren’t wasting like school time . . . but you still have a break to go outside, get fresh air and . . . but as activity, we go outside sometimes, and also we have lifestyle class at the end of the day.

**MODERATOR 1:**  Yeah, good thinking.  So one other thing we’re thinking about doing is trying to get open gym time in the morning with like a little bit of structure.  So, yeah, that would be another reason why you wouldn’t need a break right away in the morning, because if you were active in the morning, then you’d be fine to go to class.  But, Moderator 2, Moderator 3, anything, any questions that could help clarify our fall program?

**TAYLOR:**  I’ve got a question.

**MODERATOR 1:**  Yes, Taylor, after you.

**TAYLOR:**  So you said we’re doing the open gym time.

**MODERATOR 1:**  Hopefully.

**TAYLOR:**  Yeah, hopefully.  What, like what time do you think?

**MODERATOR 1:**  We’re not certain yet.  We’re going to work on that with you guys and adults and the principal and superintendent in the next couple months.  But hopefully, maybe something like 7:15 to 8:00 or so, something like that.

**TAYLOR:**  Because like there’s so many buses I can ride to get to the elementary school, but there’s one that like it gets there at like 7:45.  I don’t know.  So because I’m like a breakfast eater, and then, you know, like it’s in a rush.  Like you got to go to breakfast, and you’ve got to go to class.  You know, we can’t have like somewhere to stop and like talk.

Like if I wanted to ask, because I’ve really been wanting to join the drama club, because I’m so good at dancing and singing.  But, you know, I was like, you know, I don’t really like to be rushed.  I like to get there, eat for about five minutes, maybe see if I can stop, see if I can get any info maybe, see maybe anything I can, you know, do or help people and then maybe go to class.

**MODERATOR 1:**  I like that.  That’s a good routine.  And I appreciate you not wanting to be rushed.  My wife, Vanessa, does not like to be rushed in the morning, so I know how that can make people grumpy.

**TAYLOR:**  And I have to get up at like 5:00 in the morning.

**MODERATOR 1:**  Oh, yeah, that’s early.  Leah?

**LEAH:**  Yeah, I don’t like waking up either.  If, my mom will not let me have coffee yet, so I am very, very tired in the morning.

**MODERATOR 1:**  Okay.  Well, that seems like a perfect reason why just a little sprinkling of activity before school might be super fun.  Moderator 3, Moderator 2, anything?

**MODERATOR 2:**  Yeah, I have one question.  So depending what, when we do it, say it’s after school, what would an ideal snack portion be for you, like an apple, a cheese stick and or more, PB&J and a fruit cup?  Like how much?  How hungry are you after school?  Leah, you want to go?

**LEAH:**  Sure.  So I am actually, I like to eat.  So I get really hungry after school, because usually our lunch isn’t that good.  Like the only lunch that I like is Pizza Hut pizza.  So I wouldn’t eat that much, and then I would come home real hungry.  So then I would eat like ten million gazillion snacks.

**MODERATOR 2:**  What type of snack?  What would you usually have after school?  What’s your go-to snack?

**LEAH:**  Well, I usually have gummies or trail mix.

**MODERATOR 2:**  And the trail mix you have, what does it have inside of it?

**LEAH:**  It has raisins, sunflower seeds, nuts, M&Ms, and M&Ms.

**MODERATOR 2:**  . . . a good part.  Thank you.  What about you, Taylor?

**TAYLOR:**  So I usually eat my lunch, not every day.  But, you know, I come home.  You know, I’m so hungry.  I preferably like dark chocolate stuff, because I don’t really like milk chocolate.  But there’s, like I like those protein bars with like salt, you know, peanuts, dark chocolate, and it’s like the bars.

**MODERATOR 2:**  Sounds good.  Thank you for sharing.  What about you, Morgan or Casey?

**CASEY:**  . . . snack is probably like maybe something like a granola bar or gummies or something little . . .

**MODERATOR 2:**  How big is your granola bar?  Is it the Nature Valley ones?

**CASEY:**  No, like you know the Dippies ones like the peanut butter or like the chocolate chip ones?

**MODERATOR 2:**  Oh, I know what you’re talking about.  Does your sister have something to say?  I see her in the background?

**MORGAN:**  I want Go-Gurt.  I love yogurt.

**CASEY:**  Yeah, and yogurt.

**MODERATOR 2:**  . . .

**CASEY:**  Like the . . . what is it, the snappable one?

**MODERATOR 2:**  The Chobani snaps, I think they’re called.  Anyone else?  Any more favorite snacks, after-school snacks?  Maya?  Well, thank you for sharing.

**MAYA:**  I mainly, I mean, when I get home I sit down, do my homework, and then I sometimes make a bowl of cereal, or I’ll just like wait for dinner, because like I’m not that much of an eater.

**MODERATOR 2:**  What type of cereal do you eat?

**MAYA:**  I eat the Fruity Pebbles but like the chocolate kind.

**MODERATOR 2:**  Okay.  All right.  I know what you’re talking about.  All right.  Well, thank you.  I have a good idea what you guys like to eat.  I wonder if Moderator 3 has anything to say.

**MODERATOR 3:**  Not really.  I enjoyed listening to all the comments the kids bring us.  And it’s fun, because they bring things you would not think of alone.  So I have really enjoyed these meetings, and I’m taking a lot of information from it.  So thank you for sharing.

**MODERATOR 1:**  Yeah, thanks, everybody.  I appreciate your time.  Thanks for coming.  And that’s one of the really cool things about including kids in research studies is you guys all have a perspective that adults miss.  Like we have all these blind spots that we don’t see, that you have all these things to add that are super important.  So I’m just happy you’re here and willing to share.  And then reminder that we won’t have a session next Thursday.  We’re going to take one week off, give you all a break, and give us a chance to break down some of the information you’ve said.

Indiana University Sport Management

Hoosier Sport

Child Design Session 3

**TAYLOR:** Yeah.

**MODERATOR 1:**  Okay.  Cool.  So

[Simultaneous discussion]

**TAYLOR:**  No, I’m not . . .

**GIRL:**  . . .

**MODERATOR 1:**  Oh, and, Taylor, it might.  Sometimes if it’s a little loud it might mute your screen.  But feel free to unmute at any time.

**TAYLOR:**  . . .

**MODERATOR 1:**  Okay.

**TAYLOR:**  There.

**MODERATOR 1:**  So let me show my screen real quick.

**TAYLOR:**  No.

**MODERATOR 1:**  And, actually, wait.  Back up.

**TAYLOR:**  I wasn’t.

**MODERATOR 1:**  So first thing today, let’s talk about sports.  So I’m interested in your favorite sport to watch and your favorite sport to play.  And we’ll just go around the screen.  Leah, do you mind going first?

**LEAH:**  Yeah, sure.  So my favorite sport to play is softball.  My favorite sport to watch is football.

**MODERATOR 1:**  Oh, cool.  Okay, softball, football, awesome.  Then let’s go to Katelyn, how about you?  Not Katelyn, Taylor.

**TAYLOR:**  My favorite sport to play is softball.  And what I really like to watch is soccer.

**MODERATOR 1:**  Oh, nice.  What do you like about watching soccer?

**TAYLOR:**  It’s just I like different players, seeing them interact and like whenever they win.

**MODERATOR 1:**  Cool.  I like it.  And, Leah, how about you, where do you, what do you like about playing softball?

**LEAH:**  I like batting and catching popups the most.

**MODERATOR 1:**  Oh, nice.  That’s super fun.  Okay.  Casey and Morgan, how about you, same question, favorite sport to play and favorite sport to watch?

**CASEY:**  My favorite sport to play is softball, and I like watching soccer, because I don’t know.

**MORGAN:**  I like playing tennis, and my, and I like to watch soccer.

**MODERATOR 1:**  Cool.  Can you two tell me about what you like about watching soccer?

**CASEY:**  I don’t know.

**MORGAN:**  I like watching the women’s girls play.

**MODERATOR 1:**  Oh, yeah, like the women’s national team or the women’s anybody team?

**MORGAN:**  Mm-hmm, just like the women’s U.S. team.

**MODERATOR 1:**  Awesome.  Yeah, they’re super good.  Did you watch the World Cup?

**MORGAN:**  No, it was super, it was like 3:00 a.m., because it was like, it was in Australia, so the time was different.

**MODERATOR 1:**  Yeah, it was tough.  You had to get up really early.

**MODERATOR 1:**  Welcome, Moderator 3.  How’s it going?

**MODERATOR 3:**  Sorry, my full screen was blocked, so I, it like, it got frozen, so I had to turn off the computer and go again.  So sorry about that.

**MODERATOR 1:**  Hey, no worries.  We’re just going around the room.  And we’ll go to Moderator 2 next, and then we’ll come to you with your favorite sport to play and your favorite sport to watch.

**MODERATOR 2:**  Okay.  My favorite sport to play is, obviously, play water polo.  But my favorite sport to watch is probably volleyball, I think beach volleyball in particular.

**MODERATOR 1:**  Oh, beach volleyball, super fun, especially Olympic time.  Cool.  And how about you, Moderator 3?

**MODERATOR 3:**  For . . . track and field, the long jump, specifically, and triple jump maybe.  But to watch, I love basketball when it’s like a high-level basketball, and everybody is like screaming and, you know, it’s more intense.  Yeah, that one.

**MODERATOR 1:**  Nice.  What do you like about long jump?

**MODERATOR 3:**  I just like that little flying through space like.  It’s very fun how you can incorporate speed and control it to take off.  That is very hard to do, and I like challenges, so I guess that’s it.

**MODERATOR 1:**  Cool.  Flying through the air, that’s awesome.  Maya, how about you, if you’re there, favorite sport to play, favorite sport to watch?

**MAYA:**  I like to watch football and play volleyball.

**MODERATOR 1:**  Oh, nice.  Okay.  So we’ve got two football watchers.  Awesome.  What do you like about playing volleyball?

**MAYA:**  I don’t know.  I’ve just done it for a really long time.

**MODERATOR 1:**  Cool.  That makes sense.

**MAYA:**  And I . . . and I, so like the physical for the games today . . .

**MODERATOR 1:**  Yeah, volleyball is tons of fun.  So the reason I, we started with that is because we need, as a group, to figure out two to four, maybe three or four sports to design this first program around.  So the first eight weeks we come to WRB, we were thinking it might be something like four weeks on one sport and four weeks on another sport, maybe something like that.

So we wanted to brainstorm with you five what the best sports that sixth grade students might be interested in, and we want to, a couple things.  We’d want it to be fun, inclusive, be able to either have the equipment or purchase the equipment.  So we wouldn’t do something like ice hockey, because like we’d need an ice rink.  But we could do all sorts of stuff, all sorts of other ones.  I don’t want to put any ideas in your head.  So let’s go around the room one more time.  And if you had to pick three or four sports that you think would be the best fit at WRB this coming semester, what would they be?  And, Leah, do you want to go first again?

**LEAH:**  Sure.  Golf, softball, volleyball, and basketball.

**MODERATOR 1:**  Cool.  Can you tell me a little bit more about what made you choose even one of those or some of those?

**LEAH:**  So they just started golf, and I wanted to do golf, and I thought that was cool.  So that’s why I said that one.  Softball, I did.  It was pretty fun.  And then basketball, I’ve done it.  I did, I’ve done bitty ball.  And then volleyball, I just like to watch sometimes.

**MODERATOR 1:**  Awesome.  Very decisive.  Good job.  Who wants to go next?  After you, Taylor, three or four, even two, three, or four sports you think would be a good fit this coming semester.

**TAYLOR:**  I was thinking maybe like football, maybe some softball, you know, volleyball, and I would say golf.

**MODERATOR 1:**  Golf.  Cool.  Can you tell me about football?  What made you choose that first?

**TAYLOR:**  Well, our school really doesn’t, like they really don’t have much like sports that we can play.  It’s really mostly volleyball.  They just started golf, so golf, softball.  But you have to be on the school team.  You can’t be like on a community team.  You know, you have to be on the school team.  So why I choose football is because I think we don’t have, we do not have a football team at our school, so maybe like a football team.

**MODERATOR 1:**  Cool.  Yeah, great ideas.  Thanks for that background info.  And remember, all these questions, all night, there’s no right or wrong answers.  So we’re just looking for your opinions.  So thanks for sharing that, Taylor.  How about Casey or Morgan, whoever wants to go first, three to four sports you think would be a good fit at WRB this year?

**MORGAN:**  Soccer, basketball, track, and I want to, them to bring tennis to like WRB . . .

**CASEY:**  . . .

**MORGAN:**  . . . like junior high tennis.

**CASEY:**  . . .

**MODERATOR 1:**  Awesome.  So would you be willing to share what made you choose soccer, track, tennis?

**CASEY:**  Because I play, I want to do track, I’m going to do track this year.  Soccer, I’ve always done soccer.  And then I do tennis lessons, and it’s really fun.  I want other kids to like want to try it too . . .

**MODERATOR 1:**  I love that.

**CASEY:**  . . . want other kids to try.

**MODERATOR 1:**  Yeah, great ideas.  And those could be really inclusive sports.  When you think about track, what type of like events are you thinking?  Are you thinking about like jumping or sprinting or like throwing events?  Is there anything you’d think would be a, like most kids would enjoy learning?

**CASEY:**  I think sprinting would be probably the, or the hurdles jumps.

**MODERATOR 1:**  Cool.  Random side note, I used to teach at a speed school, which was all about teaching kids proper sprint form.  So that’s super fun.  I love that idea.  Then how about, tell me about soccer.  How do you think that would be received both by, I’m, we’re looking for something that both boys and girls would totally be into, do you think there’s any sport?  Let me see.  Hold on.  Let me rephrase that.  I’m making it too complicated.  Soccer and tennis, do you think they would be well received by both boys and girls?

**CASEY:**  Yeah.

**MORGAN:**  Yeah.

**MODERATOR 1:**  Okay.  Awesome.  How about your sister, three or four sports?

**MORGAN:**  Soccer, basketball, and track.

**MODERATOR 1:**  Cool.  And how about with track, what do you think would be the coolest events to teach, the most well received events?

**MORGAN:**  Jumping.

**MODERATOR 1:**  Jumping.  Cool.  High jump, long jump, or both, or triple jump, or all the jumping?

**MORGAN:**  Mm-hmm.

**MODERATOR 1:**  All?  Cool.  Okay.  Great ideas.  Okay.  And then, Maya, how about you, three or four sports you think would be well received by students a little bit younger than you?

**MAYA:**  Kickball, soccer, basketball, and softball.

**MODERATOR 1:**  What was the first one you said, did you say kickball?

**MAYA:**  Yeah.

**MODERATOR 1:**  Oh, cool.  Tell me about that.  Have you, yeah, what made you choose kickball?

**MAYA:**  Didn’t, like didn’t you ask what sports would be best for like people younger than me?

**MODERATOR 1:**  Yeah.

**MAYA:**  Oh, okay.  Then the kickball I thought because like, you know, it’s not like that active.  It’s like kind of simple.  You just like get the ball rolled to you and you kick it and run to a base.  And like you don’t have to like fully active yourself for the game.  It . . .

**MODERATOR 1:**  Yeah, I like that.  Great idea.  No, you wouldn’t have to be running all the time.  You know, like some sports you’d be running more than others, and that’s a good one.  Mm-hmm, I’ve got Corgis running around.  Okay.  Good.  So we’ve got lots of school sports.  Anything else on that, Moderator 2, Moderator 3, on sport ideas, sport questions?

**MODERATOR 2:**  I was going to say like field hockey and lacrosse, but those are not inclusive for both genders, but they give the opportunity for both boys and girls to play some stick sport.

**MODERATOR 1:**  Yeah, that’s cool and pretty minimal outside getting the, purchasing the sticks.  Moderator 3, anything else on sport ideas or sport questions?

**MODERATOR 3:**  I was going to offer track, but it was already offered, because I’m biased on that one.  And then I was thinking actually about tennis, but I don’t know if we have a facility for that.  Maybe pickleball can be substituted or . . .

**MODERATOR 1:**  Yeah, pickleball.  Yeah, tennis.  I’m guessing, so we’d probably need to do it in the gym, but, yeah, awesome.  Okay.  Great ideas.  We’ve got softball, volleyball, basketball, football, golf, track, tennis, kickball, field hockey, lacrosse, all sorts of good stuff.  So we’ll work with you guys and some adults over the next like six weeks or so on narrowing down which ones.  Then let’s move onto, let me show you some survey results that are cool in a simple way.  Okay.  Can you guys see the screen okay with, is it big enough?

**GIRL:**  Yeah.

**MODERATOR 1:**  Okay.  Awesome.  So this was a survey we sent out to some of you and to some adults and to some IU students like Moderator 2 and Moderator 3.  And we got, we had people rate what they thought were the most important program ideas.  These green ones were the highest-scoring ones, and they’re above a nine.  And then in yellow, you don’t need to memorize all these, but in the yellow are kind of medium, they are in the eights.  And then the white ones down here are sevens.

So this doesn’t mean like we’re not going to include any of the white ones or that we’re only going to do these green ones up here.  It’s just like one tool to try to figure out what everybody thinks are the most important things.  So the highestscoring one we’re going to talk about first is positive peer mentoring.  So that is, we’re trying to figure out what that would look like.  So that would be, let me ask you all a question.  I’ve asked something similar in the past, but can you tell me a story or an example of somebody either your age or a little older than you being a positive peer mentor for you?

**TAYLOR:**  You want to make . . .

**MODERATOR 1:**  Leah, any ideas?  Can you think of someone either your age or a little older than you that had a like a positive experience with you that like positively influenced you, yeah?

**LEAH:**  Jennifer, she is so nice.  I, she will get me to read a book, or she will get me to go to the library.  She’ll get me to do a lot of good things.

**MODERATOR 1:**  Cool.  Is she a, is she your age?  Is she a little older than you?

**LEAH:**  Yeah, she’s my best friend.

**MODERATOR 1:**  Oh, that’s sweet.  How awesome.  Jennifer sounds great.  How about what, wait, let me ask you one more question about that, Leah.  So Jennifer, what is it that she does that like helps you listen to her or helps you want to like do those positive things?  What is it about her that makes you feel positive about it?

**LEAH:**  She has high energy, so she’ll really get me to do it, or she’ll keep begging me to do it.  I’ll be like, okay, fine.

**MODERATOR 1:**  Okay.  Cool.  Good energy, persistent.  How about Taylor or anyone else, another story or example of someone either your age or a little older?

**MAYA:**  My friend . . . she, like when we’re in gym or something, she like, every time I like mess up on something she doesn’t like, she’s one of the ones that doesn’t like judge me for it or like laugh.  Like she is like helpful.  Like she basically taught me how to play volleyball.  She first joined volleyball, and then she wanted me to join.  And I told her, I was like, I can’t do that.  I don’t know anything about it.  And then she taught me for like a week straight, and then now I’ve been doing volleyball for almost two years, I’d say.

**MODERATOR 1:**  Oh, awesome, Maya.  What is it, what would you say it is about her that like allows you to like hear her and take on her friendship, her mentorship?  What does she do to make that happen?

**MAYA:**  I don’t really know what you mean by that question.

**MODERATOR 1:**  Let me try to rephrase.  How would you describe her personality or how she acts to . . .

**MAYA:**  She’s kind, respectful, friendly, trustworthy.  It’s, she’s like someone I can trust for like almost everything.

**MODERATOR 1:**  Okay.  Cool.  Thanks for sharing that, Maya.  Super helpful.  Taylor, do you want to go next?

**TAYLOR:**  Sure.  Well, is it okay if I do myself?

**MODERATOR 1:**  Yeah, of course.

**TAYLOR:**  So I love, and, I mean, I love reading.  And I have all different sorts of books.  I really love reading and doing different crafts.  And if like somebody gives me like a paper and scissors and tells me to make something, I will try to make something work.  But I am like I really like doing stuff.  I do.  I like playing with my little sister.  I like, of course, reading and doing crafts.  But that stuff makes me energized, because I think of it like so much that it just makes me energize and wants me to do that, like do everything.

Like if I wanted to make like my own like study group, then, you know, I would be reading and that.  I’d kind of be crafty.  I know I wouldn’t like see my sister a whole lot, but, you know, that like if I wanted to make something, then I have the energize to do it, because I’m thinking of that and not just, oh, let’s not, you know, let’s not play with your sister.  Let’s not read, and let’s not do this.  Let’s just be boring.

**MODERATOR 1:**  That makes sense, Taylor.  Thanks for sharing that.  How old is your sister?

**TAYLOR:**  She’s going to be two in December.

**MODERATOR 1:**  Oh, she’s very young.  Oh, cool.  Can you tell me an example or us an example of some time you have in the past helped somebody to either read or to make them something or what that looked like?

**TAYLOR:**  Well, I really like making my parents crafts and my friends crafts.  I really love to.  I like giving my sister a whole bunch.  But mostly I kind of get people like energized to like make their own like art group or reading group or, so that, it’s not . . .

**MODERATOR 1:**  Perfect.  Thanks . . .

**TAYLOR:**  . . . just, you know, I’m not just, oh, let’s not go teach somebody how to read.  Or because half of the boys in our class don’t even want to read, and some of the girls, like half of our girls and half of our boys don’t even want to read.  But it’s, I really like to read, so I like to make my own groups with Leah and Jennifer.  And I’m actually in one of their groups right now.

**MODERATOR 1:**  Nice.  That’s cool.  Great example of being a positive peer mentor and in reading, such an important topic.  So that’s a really good one.  How about Casey and, oh, Leah, after you?

**LEAH:**  Okay.  So the group actually is the reading group.  So I’ve been trying to get people to practice reading with us so we can get points, and we can keep reading, we can get our reading level up and all that stuff.

**MODERATOR 1:**  So either of you, could you tell me about how you pick people to be a part of that, because one of the questions later is how should we best like pair people up, or what kind of strategies have you liked in the past to try to find a good connection?  Because, you know, you don’t jive with everybody.  I forgot what the boy’s name was that’s really loud.  But it’s important to try to pair you up best.  So how do you pick your, those people in the groups that you target and anything you’ve learned about pairing up people to work with?

**LEAH:**  So what we did is we put no craziness for the sign-in paper to be in the group.  We put no craziness.  And so if it’s a girly book, the girls will get to read it.  If it’s a boy, a more boy book, the boys can go read it.  And then if it’s a book that everyone would enjoy, we would all read it together.

**MODERATOR 1:**  No craziness, that is a good, foundational rule to join the club.  Casey and Morgan, how about you, could you tell me a story about either you mentoring somebody or somebody mentoring you that’s either your age or a little older, little younger?

**MORGAN:**  Casey, go first.

**CASEY:**  I don’t really know.  I’ve got to think about it.  Morgan, you go.

**MODERATOR 1:**  Yeah, no worries.  Morgan, after you.

**MORGAN:**  Probably my best friend, Darla.

**MODERATOR 1:**  And what does she do or how does she, you know, tell me a little about what she does and how she does it that works well?

**MORGAN:**  Oh, geez, she helps me with my homework so I . . . do it.

**MODERATOR 1:**  Awesome.  Okay.  And, oh, by the way, so if you guys ever don’t know an answer, you can always say just like pass or you’re not sure.  Like don’t feel like you have to answer every question.  So it’s totally okay that your sister wasn’t sure of an answer for that one.

Let’s talk a little bit more about partnering people up.  So all of you have examples from classes where you’ve had to work with people that you’ve either liked or not liked.  If we, when we start Hoosier Sport at WRB this fall, one of the things, based on this survey and other stuff, is that peer mentoring might be a cool thing to include in the program.

But we’re wondering what you five think is the best approach for partnering people up.  Because like on one hand, we could just do it randomly.  On the other hand, we could make sure boys are with boys, girls are with girls.  We could try to find people that like similar things.  So what are some ideas that you have?  And, Taylor, after you.  I’m muted telling you that you’re muted.

**TAYLOR:**  So the way our class does, we really don’t partner up mostly, which I really don’t like.  So we, so I like to be with my best friends.  I do.  So, and half the people in my class don’t really get along with me.  I don’t know about Leah.

**LEAH:**  I do get along with you.  Don’t worry, girl.

**TAYLOR:**  I mean, other people like . . .

**MODERATOR 1:**  Okay.  How about you, Leah, ideas for what would be the best way, since we’re coming into this, and we don’t know all of you?  We know you five, so we could figure out how to partner you up best.  But with kids we don’t know, what would be the best way to partner, to pair people up with mentors?

**LEAH:**  Maybe first they could talk to each other first and get to know each other first, before they do that, before they get partnered and see if they’re compatible.  If not, they can switch partners.

**MODERATOR 1:**  That is an awesome idea.  So there’s this thing called speed dating that doesn’t need to be about dating.  It can just be about meeting new people.  And you have like 60 seconds with somebody, and you talk about X, Y, and Z.  So that could be something.  Great idea.  Maya or Casey and Morgan, ideas?

**MAYA:**  Can you repeat the question?

**MODERATOR 1:**  The question is if we were going to try to partner people up or kind of set up some kind of mentorship system at WRB, what would be some good ideas for pairing people up?  How would you do it?

**MAYA:**  Put an even amount of girls and boys on the same team so it’s just not like girls versus boys.  Because like I don’t know.  I just have a thing about that.  I don’t like that part when they just like put girls on one team and boys on the other so like to do like a mixture.  And then at WRB they put like an even amount of people on a team, and I just don’t think that’s okay and . . .

**MODERATOR 1:**  That makes sense.  Let me ask you five real quick, a quick question.  Do you think boys should only work with boys, or if, and, or and girls should only work with girls, or do you think it’s fine to mix it up?  Casey, Morgan, what do you two think?

**CASEY:**  I think it’s good to mix it up, because sometimes if you’re just . . . like the boys will just mess around, and sometimes the girls would, if you’re just doing all the girls.  So you have like an equal amount, so you’re still like having fun, but you’re not like distracted.

**MODERATOR 1:**  Oh, good point.  Yeah, it might be a little too silly.  Taylor, how about you, just boys with boys and girls with girls or mix it up?

**TAYLOR:**  You know, I really like hanging out with some of the boys, I’m not going to lie.  Some of them are okay.  Some of them are meh.  And some of them are just get out of here.  But I think we should mix it up.

**MODERATOR 1:**  Okay.  Cool . . .

**TAYLOR:**  Because I don’t really like, because some of the girls in my or our class, they love, and, I mean, they love to mess around.  Like I know them, and I like ask them if they get their homework done, and they say that they don’t even get their homework done.

**MODERATOR 1:**  Yeah, that makes sense, Taylor.  Okay.  So I’m hearing mix it up.  Let’s move on from peer mentoring and go to a next topic.  So one of the other things that was ranked really high, that is like a really tangible thing we could implement, is having IU athletes come from the University and talk with WRB students.  So just like Moderator 2 being a water polo athlete at IU and Moderator 3 being a long-jump athlete at IU, they’re perfect examples, some people that like talk to you about whatever you want to talk about.

It could be in small groups.  It could be in large groups.  It could be on any topic.  So I’m interested from you five in topics you think would be cool to have IU students come talk about with you, with small groups or with your classes, either one.  Who wants to go first?

**TAYLOR:**  I guess I will.

**MODERATOR 1:**  Taylor, after you.  Cool topics, what do you think?

**TAYLOR:**  I think we should do it in a smaller group.  That way, you know, because some other kids, you know, might get lost and say what was that again, you know, and then they don’t know what it was.  And I think what we should talk about is like kind of our future kind of ahead of us, you know, if we really want to go to college or IU or, you know, to the State and learn stuff, or should we just like, you know, I don’t know, just, oh, I don’t really want to go to college.  But me, I really want to go to college.  I really want to learn stuff, because my dream job is to be a veterinarian.

**MODERATOR 1:**  Cool.  Thanks for sharing that, Taylor.  Yeah, they could have some really good insight into that stuff.  Leah, how about you since you’re there?  What would be some cool topics you’d like to hear from IU students coming to WRB?

**LEAH:**  So hold on.  What was the question again?

**MODERATOR 1:**  What do you think would be some cool topics to have these really awesome students come from IU to talk with you guys at school?  What do, what would you like some topics to be?

**LEAH:**  Yeah, I do like Taylor talk about our future.  But maybe talk about how to get to know each other better, maybe even our teachers.

**MODERATOR 1:**  Cool.  And when you say get to know each other better, do you mean like social skills, relationship-building stuff, that kind of stuff?

**LEAH:**  Yes.

**MODERATOR 1:**  Okay.  Awesome.  Maya, Casey, or Morgan, how about you?  What are some, what are a couple cool topics or even one cool topic you’d love to have the content be about?

**CASEY:**  I would like to like, if someone like plays a sport like them get to talk to someone that plays their sport at like IU so you can kind of learn from them.  Instead of just talking to anybody, just talk to like someone that plays that sport that you want to like learn about.

**MODERATOR 1:**  Great idea.  Thanks for sharing that.  And your sister?

**CASEY:**  Morgan?  She’s in the bathroom.

**MODERATOR 1:**  All right.  We’ll pass on her.  Maya, how about you, some topics that might be cool to hear about from some college students that are total rock stars?

**MAYA:**  Maybe some like helpful information about like to like . . . your goal in like the sport you’re playing and like for them to like communicate like on like what to do better to like help you later in life in the sport or something.

**MODERATOR 1:**  Great idea.  And how about, oh, let me ask Moderator 2 and Moderator 3 first, actually.  What do you think?  Is there anything you want to ask about like in terms of other topics that would be good or not good?  Because, of course, like college and life and everything is about way more than sports.  So I do love coming to talk about sport, but then there’s also other stuff too.  So what questions, what question do you two have about other topics or other ideas so we can, because we’ve got the best source right here, these five?

**MODERATOR 2:**  Yeah, so when you guys are getting like into the middle school age era and then you go onto high school, I know when I was in middle school they kept talking to me about like doing extra, like being an ambassador for the school, doing more for your resume for college.  Would you guys be open to learning how to build your skills and your resume going into high school at all, or is that too early for you guys yet?

**MODERATOR 1:**  After you, Leah.

**LEAH:**  . . .

**MODERATOR 1:**  Oh, I thought you had your hand up.  Sorry.

**LEAH:**  Oh . . .

**MODERATOR 1:**  So what do you guys think, resume building for getting into high school and college.  Are you, is that on your, are you paying attention to that yet, or is that a little too early?  Taylor?

**TAYLOR:**  I don’t really, since I’m in sixth grade, I mean, people in this might be like in seventh or eighth grade.  But I don’t really think that’s too early.  I think, you know, they should be at least thinking of whether they want to go to college or if they want to go to high school.  So, but I don’t think it’s too early.  I mean, I was like maybe ten or nine or ten whenever I thought I really wanted to go to college.

**MODERATOR 1:**  Thanks, Taylor.  That makes sense.  Maya or Casey, Morgan, what do you think about that?

**MAYA:**  I’ve always wanted to go to college.  Like even though that I’m not like a big school fan, I just, like my mom suggested, like if I do go to college, it will like help with my life career or like the, like my dream or like what my goal is to do in life.  She said if I don’t go to college, then I won’t get like a good degree or anything or like a good job.

**MODERATOR 1:**  Thanks, Maya.  Leah, you want to add anything to that or no?

**LEAH:**  No, I’m good.

**MODERATOR 1:**  No, okay.  Cool.  Moderator 3, any topics you’re interested in asking about?

**MODERATOR 3:**  Yeah, I was actually curious in the same line as Moderator 2 about the future but in a different aspect.  It was about, if the kids at this age start thinking about scholarships for sports.  Because I know most, I’ve been a volunteer coach here at IU for two years now, and I’ve noticed the kids that come here, sometimes they think they don’t have enough talent or whatever to do a varsity team, universities.

And what . . . kids think about sports at this age and if you have been thinking about scholarships.  Because I come from a different country so it, college was not a topic for me until I was in high school.  But I know for some kids that I’ve taught, that during elementary school parents start talking about that.  So I was just curious about that.

And maybe we can, if your goal is to start mastering a sport at this age, this is more or less the age I started doing sports, maybe we can pay a little bit more of attention.  Instead of just doing a health-focused project, like maybe we can start training a little, at least in what we have.  If we have a water polo coach or a track and field coach present, we can help you get that little extra attention for development.

**MODERATOR 1:**  What do we think, scholarships, sport-specific details?  Casey?  Morgan?

**MORGAN:**  No, not really.

**CASEY:**  I want to be, I want to go to art school, but I don’t really want to play sports in college very much.  Yeah, I don’t really want to play sports too much, because I want to be a cartoonist when I grow up, so . . .

**MODERATOR 1:**  Cool.  Thanks for sharing that.  Anybody else?  Leah?

**LEAH:**  I would like to go to college and high school, because I want to be an engineer.

**MODERATOR 1:**  Cool.  I love that.  So I’m hearing a little more like life goals, kind of career path type things.  That totally makes sense.  Taylor, after you.

**TAYLOR:**  So, yeah, so I was talking about being a veterinarian.  I, and I really love dogs.  I really love cats.  I mean, if you, I mean, if I see an animal, I get so excited.  But I also love working on cars.  My dad teaches me how to like change the tires and do the oil and do the batteries and stuff.

**MODERATOR 1:**  Awesome.  Thanks for sharing that, Taylor.  So . . . ready to move on from that, Moderator 2, Moderator 3?  So we like the idea, and we’ll narrow in on the topics.  We have tons of cool college students that I think would be willing, that would be able to get come speak to all of you.  It should be super fun.  And I, yeah, I think some big groups would be okay.  I think it would be cool if we could also get some small group chats going, because then more people get involved.

How about a quick question, and then we’ll probably finish up with one more open one.  I’m curious if you five think that peer mentoring would be better if it’s all within the same grade or if you think it would be better if older kids mentored younger students.  Which of those two, in general, would be a better idea?

**MAYA:**  I think if older kids would like mentor like younger students to get them like ready for what they’re going to go through when they get older.

**MODERATOR 1:**  Yeah, good point, Maya.  Taylor, Leah, Casey, Morgan, do you think same grade, or do you think the different age model would be better?

**LEAH:**  I . . . a different age model would be better, because like what the other person said, it would be like better to like teach them what they know so then you can kind of go off that and . . . the older kid would do more of the teaching than as what kids in the same grade might just know the same thing so they wouldn’t be learning anything new . . .

**MODERATOR 1:**  Yeah, good point, really good point.

**TAYLOR:**  I think older kids should, because I just think they should be ready for what’s to come and everything.

**MODERATOR 1:**  Yeah, that makes sense.  They’ve been through it kind of like the college students coming to WRB too.  They would have been through some of the challenges that you all have been through.  Okay.  So I’m hearing older and younger would be better than same-same.  Awesome.

How about, so I have one last question, and then we’ll see if Moderator 2 and Moderator 3 have anything else.  I’m curious about short physical activity breaks during the school day.  So that was an item that scored really high that might be something we could try to implement at your school.

So you all are most familiar with your, how your class time is spent, how much screen time you have, like when do you get super tired, all that kind of stuff.  If you were to be in charge, you were the principal of the school, how would you design short physical activity breaks during the day?  What would that look like to you?  Leah, after you.

**LEAH:**  So what it would look like to me is I would think maybe a 20-minute, 30-minute break.  Because we stay inside for probably 6 hours and 45 minutes a day, and we only go out for 15 minutes, like isn’t very long, 10, 15 minutes.  It isn’t very long.  So I would have it 20 to 30 minutes for exercise, or we could split that up and have 2 recesses, have both 15 minutes’ recess.

**MODERATOR 1:**  Thanks, Leah.  Great ideas.  And do you think those, if it was just one break, do you think it would be better in the morning or the afternoon?

**LEAH:**  I would think the afternoon.

**MODERATOR 1:**  Cool.  Okay.  Next, if you were the principal, Taylor, how would you design these breaks?

**TAYLOR:**  All right.  So if I was the principal, I would decide like, so like we, okay, so I don’t know if our class even earned it, but we haven’t been going outside really much like every day.  But, because like I don’t even know if our class can earn it.  I don’t really know.  But so we have been like stuck inside.  Doggies.  So we have been like stuck inside . . . they’re cute.  We have been like stuck inside, and like we like we’ve been doing like work after work, and, you know, we haven’t really got taken outside.  So I think maybe we should do like 20 minutes in the morning and then maybe like 10 minutes in the afternoon.

**MODERATOR 1:**  Perfect.  Thanks, Taylor.  Thanks for sharing that.  Yes, Casey, Morgan, if you were principal, how would you design these . . .

**CASEY:**  I would do like a not a break in the morning, because like you just got there.  You don’t really need to.  Like not too much, because you just like you just got up.  I mean, you were outside probably getting off the bus and stuff.  So you’re still outside.  But like in the middle of the day, I would say you probably need like not too long of a . . . but a shorter break, like a short break so you aren’t wasting like school time . . . but you still have a break to go outside, get fresh air and . . . but as activity, we go outside sometimes, and also we have lifestyle class at the end of the day.

**MODERATOR 1:**  Yeah, good thinking.  So one other thing we’re thinking about doing is trying to get open gym time in the morning with like a little bit of structure.  So, yeah, that would be another reason why you wouldn’t need a break right away in the morning, because if you were active in the morning, then you’d be fine to go to class.  But, Moderator 2, Moderator 3, anything, any questions that could help clarify our fall program?

**TAYLOR:**  I’ve got a question.

**MODERATOR 1:**  Yes, Taylor, after you.

**TAYLOR:**  So you said we’re doing the open gym time.

**MODERATOR 1:**  Hopefully.

**TAYLOR:**  Yeah, hopefully.  What, like what time do you think?

**MODERATOR 1:**  We’re not certain yet.  We’re going to work on that with you guys and adults and the principal and superintendent in the next couple months.  But hopefully, maybe something like 7:15 to 8:00 or so, something like that.

**TAYLOR:**  Because like there’s so many buses I can ride to get to the elementary school, but there’s one that like it gets there at like 7:45.  I don’t know.  So because I’m like a breakfast eater, and then, you know, like it’s in a rush.  Like you got to go to breakfast, and you’ve got to go to class.  You know, we can’t have like somewhere to stop and like talk.

Like if I wanted to ask, because I’ve really been wanting to join the drama club, because I’m so good at dancing and singing.  But, you know, I was like, you know, I don’t really like to be rushed.  I like to get there, eat for about five minutes, maybe see if I can stop, see if I can get any info maybe, see maybe anything I can, you know, do or help people and then maybe go to class.

**MODERATOR 1:**  I like that.  That’s a good routine.  And I appreciate you not wanting to be rushed.  My wife, Vanessa, does not like to be rushed in the morning, so I know how that can make people grumpy.

**TAYLOR:**  And I have to get up at like 5:00 in the morning.

**MODERATOR 1:**  Oh, yeah, that’s early.  Leah?

**LEAH:**  Yeah, I don’t like waking up either.  If, my mom will not let me have coffee yet, so I am very, very tired in the morning.

**MODERATOR 1:**  Okay.  Well, that seems like a perfect reason why just a little sprinkling of activity before school might be super fun.  Moderator 3, Moderator 2, anything?

**MODERATOR 2:**  Yeah, I have one question.  So depending what, when we do it, say it’s after school, what would an ideal snack portion be for you, like an apple, a cheese stick and or more, PB&J and a fruit cup?  Like how much?  How hungry are you after school?  Leah, you want to go?

**LEAH:**  Sure.  So I am actually, I like to eat.  So I get really hungry after school, because usually our lunch isn’t that good.  Like the only lunch that I like is Pizza Hut pizza.  So I wouldn’t eat that much, and then I would come home real hungry.  So then I would eat like ten million gazillion snacks.

**MODERATOR 2:**  What type of snack?  What would you usually have after school?  What’s your go-to snack?

**LEAH:**  Well, I usually have gummies or trail mix.

**MODERATOR 2:**  And the trail mix you have, what does it have inside of it?

**LEAH:**  It has raisins, sunflower seeds, nuts, M&Ms, and M&Ms.

**MODERATOR 2:**  . . . a good part.  Thank you.  What about you, Taylor?

**TAYLOR:**  So I usually eat my lunch, not every day.  But, you know, I come home.  You know, I’m so hungry.  I preferably like dark chocolate stuff, because I don’t really like milk chocolate.  But there’s, like I like those protein bars with like salt, you know, peanuts, dark chocolate, and it’s like the bars.

**MODERATOR 2:**  Sounds good.  Thank you for sharing.  What about you, Morgan or Casey?

**CASEY:**  . . . snack is probably like maybe something like a granola bar or gummies or something little . . .

**MODERATOR 2:**  How big is your granola bar?  Is it the Nature Valley ones?

**CASEY:**  No, like you know the Dippies ones like the peanut butter or like the chocolate chip ones?

**MODERATOR 2:**  Oh, I know what you’re talking about.  Does your sister have something to say?  I see her in the background?

**MORGAN:**  I want Go-Gurt.  I love yogurt.

**CASEY:**  Yeah, and yogurt.

**MODERATOR 2:**  . . .

**CASEY:**  Like the . . . what is it, the snappable one?

**MODERATOR 2:**  The Chobani snaps, I think they’re called.  Anyone else?  Any more favorite snacks, after-school snacks?  Maya?  Well, thank you for sharing.

**MAYA:**  I mainly, I mean, when I get home I sit down, do my homework, and then I sometimes make a bowl of cereal, or I’ll just like wait for dinner, because like I’m not that much of an eater.

**MODERATOR 2:**  What type of cereal do you eat?

**MAYA:**  I eat the Fruity Pebbles but like the chocolate kind.

**MODERATOR 2:**  Okay.  All right.  I know what you’re talking about.  All right.  Well, thank you.  I have a good idea what you guys like to eat.  I wonder if Moderator 3 has anything to say.

**MODERATOR 3:**  Not really.  I enjoyed listening to all the comments the kids bring us.  And it’s fun, because they bring things you would not think of alone.  So I have really enjoyed these meetings, and I’m taking a lot of information from it.  So thank you for sharing.

**MODERATOR 1:**  Yeah, thanks, everybody.  I appreciate your time.  Thanks for coming.  And that’s one of the really cool things about including kids in research studies is you guys all have a perspective that adults miss.  Like we have all these blind spots that we don’t see, that you have all these things to add that are super important.  So I’m just happy you’re here and willing to share.  And then reminder that we won’t have a session next Thursday.  We’re going to take one week off, give you all a break, and give us a chance to break down some of the information you’ve said.

Indiana University Sport Management

Hoosier Sport

Child Design Session 5

**MODERATOR 1:**  Awesome.  All right, Taylor.  So question was best experiences.  Hoping you can tell us something that you’ve liked about this whole process.

**TAYLOR:**  Well, I like that I get to learn and talk about like different kinds of stuff like how we are trying to, try to get this program in the school.  And that’s what I like about this.

**MODERATOR 1:**  Thanks, Taylor.  I appreciate you sharing that.  And if you don’t mind, could you tell me, so you liked talking about some stuff, are there, can you give me some examples of things that you’ve enjoyed talking about, even a topic or two?

**TAYLOR:**  I like how we have been talking about like soccer, basketball, volleyball, tennis, different kinds of stuff like that.  And just me talking in general to all these, to all you guys, it’s kind of fun.

**MODERATOR 1:**  Beautiful.  I’ll take kind of fun any day.  Perfect.  So thanks for being courageous and going first.  And, Leah or Maya, if one of you want to go next, anything you can tell us about something you’ve liked with this whole codesign process, and you can take that anywhere you want.

**MAYA:**  Well, I liked it because we did get to tell you guys about the stuff that we really wanted to tell somebody, and, you know, we might have been too afraid to say it.  And then here we can just say it if we need to.

**MODERATOR 1:**  That is a very cool example.  So that would be like you’ve been, you have a voice, you have a platform to say these things that are on your mind.  Like you probably think about them in school . . . have a place to share that.  So that’s cool.  Leah, can you give us an example of something you remember either you or someone else sharing that you just thought it was cool that you could express that?

**LEAH:**  That I wanted Legos in the school.  I just really wanted Legos in the school.

**MODERATOR 1:**  Perfect.  And random follow-up question for you.  Do you, why don’t I circle back to this, actually, Leah, do you often get asked your opinion on things like this, like your math teacher or PE teacher or other people, do you get asked your opinion often on this type of stuff or not, or is this pretty unusual?

**LEAH:**  Not really.

**MODERATOR 1:**  Okay.  Cool.  So let’s go to Maya, if you’re there.  Best experience.  So tell us something that you liked about this whole codesign process.

**MAYA:**  . . . like how you’ve been trying to get us to be more like energetic and more outgoing on what like sport we play, and like how to like, I don’t know, make classes better than what they are, I guess.  I don’t know how to explain that.

**MODERATOR 1:**  That makes sense.  So you like the idea that we’re trying to get things to be more fun, more improve your classes, just trying make your experience better, does that sound right?

**MAYA:**  Yeah.

**MODERATOR 1:**  Cool.  Well, that, I’m glad you feel that way, because, yeah, this stuff is all about you guys.  We want to improve that stuff at WRV.  So that is excellent.  Thanks for sharing that, Maya.  And then Casey and Morgan, how about you, anything to add on things you’ve enjoyed about this process?

**MORGAN:**  We liked that you let us like share your ideas on it.

**MODERATOR 1:**  Awesome.  And how about you, Casey or Morgan?

**CASEY:**  I liked that you don’t have to be a sports person.  Not everybody, like you didn’t get like dis-included from it.

**MODERATOR 1:**  Awesome.  Yeah.  Great suggestion.  Great comment.  Yeah.  We want to include different opinions and different points of view, and you are all on the frontlines of these classes, so you’re the perfect people to know what different people want.

Next question for everybody is what are, and this can be related to specific stuff we’ve covered or other like topics at school, that kind of stuff, what are some things, maybe one or two things, that you think you can describe in your classes that adults might not see as clearly or as easily?  Taylor, after you.

**TAYLOR:**  Well, so we, so in our class we have, kids always play around a lot who like to talk whenever the teacher is talking, and they don’t, like sometimes they don’t see that a lot that me and other people do see.

**MODERATOR 1:**  Thanks, Taylor.  Good point.  That reminds me of like a lot of kids might be a certain way in front of the teacher, but then when they’re around you, they’re different, and you would get to see their like more real behaviors.  So that’s a great one.  Leah, how about you?

**LEAH:**  So there is this kid in my class.  He likes to eat his lunch during class.  The teacher doesn’t see that, and a lot of other people see it.  And he gets, he eats all of his lunch during class, and then at lunch, he doesn’t have anything to eat.  And he’s loud when he eats, which distracts all of us, so.

**MODERATOR 1:**  Great example, Leah, a loud eater.  How about Maya?

**MAYA:**  What was the question?

**MODERATOR 1:**  What are some things that you think, I’m trying to get at the really important part of including kids in this whole research process, so the question is what are some things, a thing or two, that you think you would be able to see in your classes that adults might not know or might not see?  So what are some, like what’s unique about your perspective compared to all of us other adults here?

**MAYA:**  I’m not so sure on that one.  That’s a trick question . . .

**MODERATOR 1:**  Hey, no worries . . . but if you think of something, just put your hand up, or I’ll swing back to you.  Casey, Morgan, how about you, what are some unique things that you think you see that adults might miss?  After you, Casey or Morgan.

**CASEY:**  This is Casey.  And some kids in like our class like sometimes like, what is it, sometimes they do stuff that they’re probably not supposed to do.  And sometimes the teacher doesn’t see that.

**MODERATOR 1:**  Yeah.  Great point.  It’s like behavioral stuff.  That totally makes sense.  Does your sister have anything to add?

**MORGAN:**  I don’t know.

**MODERATOR 1:**  Okay.  Awesome.  And before I go onto the next one, Moderator 3, Moderator 2, or Moderator 4, do you have a question that you want to add, or do you want, or feel free to pitch it back to me?

**MODERATOR 2:**  I think you guys brought some great points.  Do, for going off Maya or Leah, when the kids are doing something disruptive in class, does the teacher ever notice, or it just kind of goes under the rug?

**MAYA:**  The second option.

**LEAH:**  She doesn’t know.  He just hides it in his desk, and.

**MODERATOR 2:**  Oh, that’s frustrating.  And do you think the teacher just kind of doesn’t want to know, or like have you guys told the teacher at all?

**LEAH:**  We haven’t told the teacher at all because we’re all too scared to.

**MODERATOR 2:**  All right.  I understand.  All right.  Well, thank you.  Maybe Moderator 4 or Moderator 3 has . . .

**MODERATOR 1:**  Moderator 4 good.  Moderator 3, good too?  Cool.  Okay.  So simple question here.  Curious about your experience with Zoom or in person.  So I’ll ask all five of you, do you think we should do these sessions on Zoom or in person in the future, and then why did you choose that answer?  Who wants to go first?  Leah, after you.

**LEAH:**  I think in person maybe would be better.  I know that meets, they would be, it would be easier to get on, but in person, I think would be better because you can actually, it’s not making, like if you’re glitching, if anything like that happens, you don’t know what they’re saying.  In person, you can’t glitch in person, so.

**MODERATOR 1:**  Hopefully not.  That makes sense.  Thanks, Leah.  Casey or Morgan, you have your hand up?

**MORGAN:**  I like to . . . the Zoom, because if you can’t get it in like during the school time, you can get busy, and you won’t be able to like make it or anything.  Like because my sister has softball all the time, and we also have soccer and other sports.  So it would be pretty hard if it wasn’t like in the school day or anything.  And then you’d be missing out on school time too if we went in person.  So I think it’s better on Zoom.

**MODERATOR 1:**  Thanks for sharing that and great reasons.  Maya, how about you?

**MAYA:**  I think maybe it would be better in person because we can actually see, like try to get more interest from somebody.  Like one girl said, like the glitch part and like having bad service or reception.  I’m not sure.

**MODERATOR 1:**  Thanks, Maya.  And then last but not least, how about Casey and Moran, or Taylor?  I forget.  Taylor, right?  You didn’t.  Yeah.  Taylor, after you.

**TAYLOR:**  Well, I think doing it in person would be a lot better.  That way, you know, I can still, you know, I can still see you, but, you know, I haven’t actually got to like meet you in, actually in person.  And, you know, some people like meeting people in person, or some people just like to do Zoom.

**MODERATOR 1:**  Great points.  So you all raise awesome ideas.  We will definitely look at both options in the future.  I think my gut tells me from your responses that if it were in, if it had to be in the evening, that it’s probably better on Zoom because you can do it from home and around your busy schedules.  But it would also be super cool if we could do it in person at school so you could leave class and come to a codesign session down in some classroom with a group of your friends.  So we’ll look at those options in the future.  We definitely want to keep doing this stuff, and we want to keep hearing your opinions on what we should do.

And then once we do run this first program this fall starting in mid-October, we’ll definitely want to get some feedback on it too.  So that’s another opportunity where we might be able to hear from you and learn from all of you.  With that said, thanks for sharing that.  And then let’s talk a little about challenges or barriers.  So was there anything that could have gone more smoothly or been better with these sessions, or any ideas you can think of when we do these next time that would be awesome?

**MAYA:**  I didn’t quite get like the question.

**MODERATOR 1:**  The question, Maya, is was there anything that could have gone more smoothly or been better with these sessions, or any ideas for things we could do in the future to improve?  After you, Taylor.

**TAYLOR:**  I think we could possibly improve on like different kinds of stuff for this.  You know, like everything is like smoothly, but like I think we could like make a couple things different maybe.

**MODERATOR 1:**  I like that, Taylor.  And don’t worry, none of you will hurt my feelings.  We are looking for ideas.  So, Leah, how about you?

**LEAH:**  Well, it could have run more smooth if it didn’t glitch, so because there’s a lot of people glitching on here.  It’s mostly glitching with Maya and so.

**MODERATOR 1:**  Yeah, that makes sense.  So and your idea for in person would solve glitching, because you said that people don’t glitch in person.  So great idea.  Casey, Morgan, you have your hand up.  What you thinking?

**CASEY:**  . . .

**MODERATOR 1:**  Speaking of glitching, we can’t quite hear you Casey and Morgan.  Maya, anything to add?

**MAYA:**  Maybe like if there would be more like adults on here to talk and like, instead of . . . like the same people back and forth.  Like there’s one or two people, the ones that didn’t like talk when they, we ask them a question, maybe we could like get people that will, I don’t know, like join in like the conversation.

**MODERATOR 1:**  Really cool point.  Yeah.  We do want people that are contributing and talking.  Awesome idea.  I’ve been super thankful to all of you, because you have been like eager and willing to speak up.  It would have been really weird if you were just silent the whole time.  So awesome.  Great point.  We want people that are communicative.

And then also another thing that could happen is like someone could talk way too much and like steamroll the whole discussion, and nobody has done that either.  So you’ve all been like super good to connect with.  Casey, Morgan, let’s try that again.  How about you?

**MORGAN:**  This is Morgan, and I think it all went smoothly.

**MODERATOR 1:**  Morgan, and it all went smoothly.  All right.  I’ll take that too.  Awesome.  Okay.  Let’s see.  Let’s shift to our next question.  We’re going to make a Uturn and talk about science.  So really interesting question for you.  I can’t wait to hear what your creative juices say.  But I just want to hear from the five of you.  What comes to mind, describe it as clearly as possible, when you think of a science experiment?  What do you think of?  Leah, after you.  There’s no right or wrong answer.

**LEAH:**  I’m thinking of like elephant toothpaste and chemicals and stuff like that.

**MODERATOR 1:**  Perfect.  And before I go to the next person, what’s going on, elephant toothpaste and chemicals, what are some things that are happening in your mind when you see that?

**LEAH:**  Elephant toothpaste is like just a foam where you mix chemicals and it just makes a big old foam.  It’s really cool.

**MODERATOR 1:**  Okay.  Cool.  Chemistry.  I like it.  Taylor, did I see your hand up?  Tell us about what you think of when you think of science experiment.

**TAYLOR:**  I think of like volcanoes erupting.

**MODERATOR 1:**  I like it.  Very clear answer.  Nicely done.  Casey, Morgan, how about you?  As many details as you can.

**CASEY:**  This is Casey.  And when I think of science, I think of . . .

**MODERATOR 1:**  Say that one more time.  I heard most of it.

**CASEY:**  When I think of science of think of mouse trap . . .

**MODERATOR 1:**  Did you say mouse track eyes?  Okay.

**CASEY:**  . . . mouse trap cards.

**MODERATOR 1:**  Okay.  Something close, close to that.  I think mouse trap something.  It cut out just a little too much.  Maya, how about you?

**MAYA:**  I think of like there’s stuff that you put in like a bowl, and then it like basically explodes into a big like foamy cloud, and it’s like different colors, and it gets like really big.

**MODERATOR 1:**  Awesome.  Thanks, Maya.  Casey and Morgan, do you have, do you mean to have your hand up?  I can lower it if you didn’t.  Oh, cool.  Okay.  Good stuff.  Thanks for sharing that.  The reason I asked that is because this is, all this is part of a larger science experiment, but often people don’t see it that way.  But we’re all either scientists or on our way to being scientists, and you’re part of a really cool science project.

So my next question related to that for you is what have you learned about science from being a part of this study?  And I’ll give you a little bit of a lead on that too.  You wouldn’t have learned a bunch about elephant toothpaste and volcanoes erupting and mousetraps, but you’ve probably learned other things.  So what were some of those?  After you, Taylor.

**TAYLOR:**  Well, I think . . . whenever I think of that I think of like learning something new.

**MODERATOR 1:**  I love that answer.  Really great point.  So just a reminder for the next person, we’ll go to the same question. . What have you learned about science from being a part of this science study?  And I love that, yeah, Taylor, great idea, you learned something new, totally.  Leah, is that a, or would you be willing to go next?

**LEAH:**  Well, I learned a lot of things.  I shouldn’t be scared to tell an adult something.  I don’t know what else though.

**MODERATOR 1:**  Cool.  Hey, that’s a super good one.  And I really appreciate that about you’re willing to share honesty throughout this stuff about what’s going on at school, and what you’d like to see and stuff, and that you won a golf cart and all those good things.  Maya, how about you, what have you learned about science from being a part of this science study?

**MAYA:**  I don’t know.  Like to be like more communicative and more talkative than what I normally am.  I guess like more communication.

**MODERATOR 1:**  Would you say you’ve been more communicative or that you learned that you are able to be more communicative?

**MAYA:**  Yeah.

**MODERATOR 1:**  Awesome.  Yeah.  You’ve done a great job communicating.  I really appreciate every time you speak up.  Casey, Morgan, how about you two, what have you learned about science from being a part of this study?

**MORGAN:**  This is Morgan, and I learned that everything, everybody is different.

**MODERATOR 1:**  Tell me more about that.  In what way?

**MORGAN:**  That like everybody has like different opinions on things like on sports and like different things that the school might need.

**MODERATOR 1:**  Oh, that’s beautiful.  Great answer.  Super important.  We have like different, yeah, different opinions, different ideas, different strengths, different weaknesses.  Just awesome.  And did your sister have anything to add?

**MORGAN:**  No.

**MODERATOR 1:**  Okay.  Very nice.  Then let’s go to, I think this is probably our last like sciency question.  Now that you’ve been a part of this research study, or I’ve been calling it a science study, how could you see this experience shaping your future experiences with science?  I’ll say it again for you.  Now that you’ve been a part of this study, how could you see this experience shaping your future experience with science?

**MAYA:**  Considering that I am not that good at science, it probably would like maybe like upgrade it.  Like help me more on what I’m doing in science.  Because I used to get like Fs in everything in science, but now I’m getting more Bs and Cs.

**MODERATOR 1:**  Heck, yeah, Maya, that is awesome.  I am so stoked that this is opening your eyes to a different type of science.  Really cool.  Thanks for sharing that.  Leah?

**LEAH:**  So since I was talking about stuff like this, there’s, I’ve done much better in science, much, much better.  If we have a science project, I’ve been doing better in those.

**MODERATOR 1:**  Very nice, Leah.  What has led to that, or why has that happened, would you, in your opinion?

**LEAH:**  Really, I don’t know.  I just learned some things, and.

**MODERATOR 1:**  Very cool.  A term I haven’t used yet, but this whole type of science we’re doing is called behavioral science.  And there’s some other like ways to describe it and stuff, but a lot of the stuff you guys were saying was more like chemistry and biology and things.  But, yeah, this is all about people and behaviors, so it’s called behavioral science.  Taylor, how about you, if you remember the question, or I can repeat it if you need to.  Oh, you’re muted, and I, yeah, we don’t want to miss these good answers.

**TAYLOR:**  Now I’m unmuted.  I am thinking about, since I’m talking about science, it’s, I would say it’s at least getting my grades up a lot higher, I think, and I am working a lot harder in that.

**MODERATOR 1:**  Awesome, Taylor.  Thanks for sharing that.  Casey, Morgan, how about you?

**CASEY:**  What’s the question?

**MODERATOR 1:**  The question is, now that you’ve been a part of a research study, how could you see this experience shaping your future experience with science?

**CASEY:**  . . . like sports activities to like help with sports . . . or with the science part, like the physical or stuff.

**MODERATOR 1:**  We caught some of that, but I missed some of that too, unfortunately.  It sounded like a super good answer.

**CASEY:**  . . . if you . . . a science project that you . . . and stuff with it.  So it would help with like the human body and like the mental . . . side and like all that stuff.

**MODERATOR 1:**  Yeah.  Awesome.  I definitely heard help with the body and the mental side of things.  Very good.  So, Moderator 2, Moderator 3, Moderator 4, anything you want to add on reflecting on the process, anything else?  And it’s okay if you say no.  Just want to make sure you have a voice.

**MODERATOR 4**:  Yeah.  I wanted to ask the girls, just at the start of this, did you have any fear that probably you were going to be involved in this, and if at all, what were some of those fears that you had when we initially contacted you to be part of this?

**MODERATOR 1:**  Can I add, before you go, Leah, I just want to add something to that.  So fear, and then also if you could tell us what allowed you to overcome that to say yes would be awesome.  After you, Leah.

**LEAH:**  Well, I really didn’t know until like the day before because my mom didn’t tell me.  And I was like, oh, man, oh, man, what am I going to do?  And she’s like all you have to do is answer questions.  I was like relieved after I heard that.

**MODERATOR 1:**  Awesome.  Thanks for sharing that, Leah.  Yeah.  And you got $200 along the way.  Who would like to go next?  Taylor, after you.

**TAYLOR:**  So my mom told me the day of, so Thursday.  So she told me that, and I was a little nervous.  I could tell.  But since I overcome that by actually telling the questions that you were answering, I thought myself they were going to be like super-duper hard, like really, really hard.  But then you asked me the question, and then it made a lot more sense and not, oh, you’re going to have to answer this super-duper hard question.

**MODERATOR 1:**  Really, really good point, Taylor.  Thanks for sharing that.  Maya, Casey, or Morgan, how about you?

**CASEY:**  I like that there was no right or wrong answers on it.

**MODERATOR 1:**  Really good point.  And how about your sister?  You’re right.  There are no right or wrong answers.

**MORGAN:**  The same thing.

**MODERATOR 1:**  Cool.  How about you, Maya, last but not least?

**MAYA:**  Can you repeat the question?

**MODERATOR 1:**  Tell us about if you were like fearful or scared to join these sessions in the beginning, and if you were, what were those things, and if you were, how did you overcome them?

**MAYA:**  At first, my mom had mentioned these meetings to me, and I was like, no, I don’t, like, no, I’m not communicative like people or talk to people.  And she was like, I mean, she said it’s good for you . . . you can, like I don’t really know how to explain it.  She said that it helps me in school.  And like at first I was struggling in school, so I was like, okay, maybe I should like try this out or something.  And then so I did.

And she was like, because I don’t talk . . . because people like to like judge me a lot, that’s what like one of the reasons why I was scared to get on here.  And so I got on here, and like I heard that everybody else was talking.  So like I was like, why not?  I was like nobody is judging you . . . so like why not just talk?  So that’s basically how I got on here.

**MODERATOR 1:**  Awesome job, Maya.  Thank you for sharing that.  We are all rock stars, and we are nearing the end.  I’m going to, so I have a different topic, question.  One of the things we, and I’m going to ask you to reflect or give us some examples or idea examples or reflection about things you remember us talking about over the past four sessions, five sessions.

So one of the things we wanted, we want to include and want to continue to talk about is a thing called autonomy, and that is basically giving people choices for things, so not forcing you to do things.  So I’d like to come at least to two or three of you at least and see if you remember anything, any topics or conversations we had that were around giving you choices.  So could you . . . Leah, after you.

**LEAH:**  Okay.  So when we were talking about gym, you’d say would you rather have this, or would you rather have this, then so . . .

**MODERATOR 1:**  Really good.  And do you want to stop there, or do you have another one or two that are at the top of your mind?

**LEAH:**  No, not really, but.

**MODERATOR 1:**  Yeah.  Cool.  How about Taylor, and then we’ll come to you, Maya.

**TAYLOR:**  I liked how you said, do you either want to have it in Zoom, or do you want to meet in person?

**MODERATOR 1:**  Really good example.  Yeah.  And that just happened a few minutes ago.  Any others on the top of your mind or want to pass?

**TAYLOR:**  I’ll pass.

**MODERATOR 1:**  Okay.  How about you, Maya, remember some choices?

**MAYA:**  Yeah.  I had them all on my mind.  How you said like I think this was like the second or third meeting we had where you were like what do you think like your gym or somebody could do to make like it like better in a way, or like what game do you like most, or what is your guys’ favorite, like what games you play, what do you do after school, or like what do you do to energize yourself?  Or there was like a lot of others, but I can’t remember them.

**MODERATOR 1:**  Really good examples, Maya.  Thank you.  And then I have a very similar question, just a different thing than choices.  So this one, there’s a thing called competence, and that is basically mastering or working on your skills or breaking things into small steps because you want to get better at them, things like that.  Do you remember any examples of us asking you about that stuff or topics that were around competence?  Moderator 3 or Moderator 2, do you have a simpler word for competence that would be helpful in this context?

**MODERATOR 3**:  Understanding.

**MODERATOR 1:**  Understanding.  Good one.  So, yeah, let me just let you three or four or five sit on that one for a little bit.  So any examples where we talked about topics where we wanted to gain understanding or gain skills or master things or get better at stuff?  Leah, after you.

**LEAH:**  I don’t know if I said this at all, but like that I get better at sports, but.

**MODERATOR 1:**  Yeah.  Great example.  So, yeah, we’ve talked about all sorts of different sports.  And we would, obviously, we wouldn’t want to just throw you into a game if we haven’t taught you how to shoot yet, you know, so great example.  Taylor, Maya, any other examples?  After you, Taylor.

**TAYLOR:**  I was thinking about like volleyball, basketball, like dodgeball and Legos and stuff like that.

**MORGAN:**  When we were talking about goals . . . goals . . . Casey, and I when we were talking about like superheroes and like what superheroes would help you for like this . . .

**MODERATOR 1:**  Yeah.  Good point Casey or Morgan.  I did hear, we did talk about that with goalsetting and setting goals towards trying to get better at stuff and superheroes.  So great points.  Maya, how about you?

**MAYA:**  I don’t really know any more off the top of my head right now.  I might later, so.

**MODERATOR 1:**  Okay.  Cool.  Yeah.  No worries.  That’s good.  So that was autonomy and competence.  Those are two super important things.  And then the next one I want to ask you about real quick is relatedness.  That’s like socializing and being connected to people around you or not connected.  It could be . . . like it could be we all have some relatedness being here together, but we could also have the opposite.  Leah, examples that you remember?

**LEAH:**  Talking to a friend, a friend getting you to go somewhere.

**MODERATOR 1:**  Yeah.  Can you tell me more about that, some more details, talking to a friend, when that came up or like what we were talking about?

**LEAH:**  I think maybe it was our second or third meeting that we talked about friends, how they, is it helpful how they influence you.  And, [name], as I said, really nice like . . .

**MODERATOR 1:**  Yes, totally.  I remember that.  She was the great role model, right?  Very cool.  Okay.  Awesome example.  Taylor, how about you?

**TAYLOR:**  I was thinking about like family.

**MODERATOR 1:**  Yeah.  Tell me what.  We definitely have.  What have we talked about with family, or . . . do you remember any of the topics we were discussing?

**TAYLOR:**  Like what did your family or friends help you with?  Like how I said like me and my little sister used to, so our mom, we she would help me on my math, and, you know, I would try to at first, and then she would help me, and.

**MODERATOR 1:**  Thanks, Taylor.  Maya, how about you, relatedness or social stuff?

**MAYA:**  I’m not very social, so I’m, like I’m hardly ever social to anybody, so.

**MODERATOR 1:**  Nothing wrong with that.  You might be an introvert, which is someone that like recharges on their own or likes to be on their own a little bit more than recharging in large groups.  Nothing wrong with that.  I’m also an introvert.  Casey, Morgan, how about you, relatedness, do you remember us talking about any like social engagement, connecting with those around you topics in our last few sessions?

**CASEY:**  I am like, with the college people coming in . . .

**MODERATOR 1:**  Yeah, really good one, college people coming in, building relatedness with them.  Awesome example.  Okay.  And then last question from me, I think.  So this one is around, how should I describe this, your school and your policies and your whole environment.

So do you remember us talking about, and can you give us an example or two, things around like recess or sports equipment or your facilities for your physical activity and sport or your like physical activity like time?  These are things that are called like policies and environments.  So do you remember any topics that we had around that stuff?  Taylor, after you.

**TAYLOR:**  So I don’t know if it was like the third, I think it was like the, yeah, the first meeting we talked about like the playground and how it needed some repair and we need swings and new basketball goals.

**MODERATOR 1:**  Excellent job, Taylor.  Great memory too, by the way.  Good work.  Maya or Leah?

**MAYA:**  Can you repeat the question?  I can’t really hear . . .

**MODERATOR 1:**  The question is, do you remember us talking about and can you give us an example or two of things we’ve talked about related to school policies or your school environment or even the community environment, that kind of stuff?  And, Leah, you can go first, and then we’ll come back to you, Maya, and . . .

**LEAH:**  I remember when we talked about the playground.  That place really needs fix, like really needs fixed.

**MODERATOR 1:**  Great example, Leah.  Yeah.  That’s on our radar.  We need to figure out that kind of stuff.  Maya, how about you?

**MAYA:**  I agree on the playground part, because of, like the slide, my first day there, the slide was broken.  And like a couple days later, I went outside and like it was all boarded up and stuff.  Then the basketball goals are, there’s like some basketball goals where it’s just metal and there’s no net.  There’s no rim for it.  There’s some that are okay, but they’re like kind of like all like banged up and stuff.

And like the grass area, like there’s a basketball goal, and then there’s like that little court.  And then like right where the grass is supposed to start, it’s like dirt instead of where the grass is supposed to be.  So you have to walk down like a dirt hill to get to the grass.  And like, yeah, it’s like really messed up outside.

**MODERATOR 1:**  Good points, Maya.  I do remember chatting about that stuff.  We’ve got some work to do.  How about you, Taylor, you have your hand up, right?

**TAYLOR:**  Yes.  So I was thinking about the slide, the way Maya was talking about how it was all boarded up.  So whenever I came to middle school for fifth grade, the slide, so the thing, the pole that was holding it up was going inside the slide.  So whenever somebody slid down it, they would have to deal with that whenever they slid down.  And then they took it off because different people were going on their belly, their back, of course, on their butt, and different stuff.

**MODERATOR 1:**  That sounds no fun.  And one thing that’s really apparent from this stuff is that the playground and the environment is really important to you guys.  And that is very cool, because I think like you guys probably know that best when we were talking about what you might know and the adults might not know, is you’re the ones that have to like that actually play on that stuff every day.  So it’s super valuable to hear your opinion and hear your insight on it.

I don’t have any other questions.  Moderator 3, Moderator 2, you’re, are you good?  Cool.  So I just want to close by saying thank you so much.  Moderator 2 will send your gift cards out.  I really have appreciated all the awesome information you guys have provided over the last five sessions.  It’s just been so much fun to work with you in this research study.  I hope you guys can all be proud that you’ve been a part of a research study now.  They’re awesome.

And, again, this is behavioral science, and not all science needs to be about volcanoes, not that there’s anything wrong with that.  But really cool experiences down the road.  I hope to see all of you at school when we start coming there in October.  And definitely stop by and say hi.  You’ll see all of us, Moderator 3, Moderator 4, and me all at different times, if you are all at the middle school.  I just I wasn’t sure if Maya, you’d be there.  But, yeah, any other thoughts, Moderator 2, Moderator 3, or Moderator 4 before we go?

**MAYA:**  I have a question.

**MODERATOR 1:**  Question?  Let’s hear it.

**MAYA:**  So about the gift card things, is there a way that I can switch instead of Amazon this time just like do the other thing this time?

**MODERATOR 2**:  Yeah.  I just, I already sent your gift card.  But I can, if you just disregard the last email, I can send you Amazon, or no?

**MODERATOR 1:**  Yeah.  Maya, your choice.  So if she sent it already, we can like void it, or you can keep it.  Which would you prefer?

**MAYA:**  It’s fine.  Since you all have already sent it, I can just like keep it that way.

**MODERATOR 1:**  Okay.  Cool.  Well, we appreciate your flexibility.  Thanks, everybody, and we’ll talk to you all again soon.  And I hope to see you at school in October.  Bye, everybody.  Thank you.
