# Supplementary material for: Human-centered participatory co-design with children and adults for a prototype lifestyle intervention and implementation strategy in a rural middle school
Source: BMC Public Health. 2024 Mar 19;24:845. doi: 10.1186/s12889-024-18351-x (PMC10949632; doi:10.1186/s12889-024-18351-x)
Supplement: Supplementary file 2 — Supplementary Material 2 [file 12889_2024_18351_MOESM2_ESM.docx]

| **Supplemental Material 1. Co-Design Code Book and Response Frequency** | | | | |  |  |
| --- | --- | --- | --- | --- | --- | --- |
| **Themes** | **Adult Frequency** | **Child Frequency** | **Code** | **Subcode** | **Detailed Description** | **Example** |
| **Autonomy** |  |  |  |  |  |  |
|  |  |  |  |  |  |  |
|  |  |  |  |  |  |  |
|  | 7 | 1 |  | Equipment | Limited availability of physical activity-related equipment | (Adult) "I remember them having these little devices, and then they tracked how many steps they took. But that’s been several years ago, and I have no idea where those little gadgets went. " |
|  | 3 | 2 | Programming choices | Participation choice | Having the ability to choose whether to participate in Hoosier Sport programming. | (Adult) "To make it mandatory, I think you’re going to get of lot of parents, a fair share of them that aren’t going to want to be, they’re forcing me, they make them go to school all day, and then I’ve got to come home, and they make me do this. " |
|  |  |  |  |  |  |  |
|  | 12 | 17 |  | PA choice | Having the ability to choose between different physical activity programming options | (Child) "I don't like running the mile every Friday in PE" |
|  | 4 | 6 |  | Academic choice | Having the ability to choose between different academic programming options | (Adult) "So, I think we could possibly maybe do a couple things like not on electronics and maybe do some on paper." |
|  | 9 | 11 |  | Non-PA choice | Having the ability to choose between non-physical activity programming options (e.g., life skills) | (Child) "I would like if they had different sets [of Legos], and then you can pick one, build it…" |
|  |  |  |  |  |  |  |
| **Competence** | 1 | 0 | Coach/teacher training | Controlling behavior | Coaches or teachers enforcing controlling behaviors that are not desirable | (Adult) "Sometimes when it's daddy or mommy ball, that becomes a problem." |
|  | 4 | 0 |  | Sportsmanship | Coaches/teachers encouraging sportsmanship, rather than just winning | (Adult) "They want to be part of that winning team." |
|  | 7 | 0 | Lack of knowledge | Nutrition choice | Providing people with knowledge to make their own nutritional choices | (Adult) "We’ll guide them as they make their lunch a little bit, but it’s their choice. If they want to eat lunch, they know where the stuff is. " |
|  | 2 | 1 | Lack of resources | Nutrition choice | Providing people with resources to have greater nutritional choices | (Adult) "I do the afterschool program, and literally sometimes they get like, you know, the cheese sticks and juice, and that’s it. It’s bad." |
|  | 1 | 2 |  | After school support | Providing people with after-school programming resources | (Adult) "She started going to that program, I would say two or three weeks into it, and she, it took time to bring up her grades, but, I mean, it helped. " |
|  | 2 | 0 | Acknowledgement | Incentives | Incentives or rewards to help people feel acknowledged for their efforts | (Adult) "They are reward-based. They like, I mean, I hate to say that, but they, whether it is some, they have to have that physical, it’s mine kind of thing. So, if we can adapt that to the kids that, I think that would be the key for them." |
|  |  |  |  |  |  |  |
|  | 6 | 0 | Skill building | Sport skills | Breaking down complex sport skills into tangible steps | (Child) "I'm really bad at batting. And it's just, you know, I practice a whole lot and, you know nothing really about batting really helped" |
|  | 2 | 0 |  | Nutrition skills | Breaking down nutrition into tangible steps |  |
|  | 5 | 1 |  | Social skills | Breaking down social skills into tangible steps | (Adult) "They don’t understand how to, conflict resolutions, they don’t know how to do that when they get upset. They don’t know how to talk it through." |
|  |  |  |  |  |  |  |
| **Relatedness** | 4 | 13 | Relationship building | Peer relationships | Establishing meaningful peer relationships | (Adult) "She didn’t get to really interact with a lot of the kids." |
|  | 8 | 3 |  | Adult relationships | Lack of interaction with authority figures | (Adult) "But there was not much interaction with the coaches." |
|  | 0 | 8 | Lack of effort | Adult relationships | Lack of engagement in building strong connections with peers | (Adult) "There was no extra effort… no extra oomph, I guess." |
|  | 1 | 5 | Coach/teacher training | Adult relationships | Defiance in coach guidance to support or training to support children | (Adult) "There was a lot more than just her that just didn’t get the help or the support or whatever from the coach or whatever they needed to try a little bit harder on or practice on" |
|  | 3 | 3 | Social skills | Peer relationships | Technology relance hinders social skills | (Adult) "I think life skills should be adding social skills because when we do so much screentime, and it is phone time and iPad." time and everything, they don’t know how to communicate with each other. |
|  | 2 | 0 |  | Screen time | Excessive screen time hinders social skills | (Adult) "The amount of screentime is unfathomable to me at this point." |
|  | 13 | 8 | Leadership | Role modeling | Children develop skills by observing role models | (Adult) "Kids would become more involved and want to do more things because they’re seeing the parents setting that example. " |
|  | 3 | 1 |  | Sportsmanship | Playing sports develops sportsmanship qualities | (Adult) “They have to be a sore loser as well as a sore winner, or a good winner, you know what I mean. I’m saying it wrong. But they can’t be, either way, they can’t, they have to be able to teach them that whether they win or lose, they’re supposed to be doing it with humility and respect." |
|  | 0 | 1 | Lack of equipment | PA opportunities | Physical development influenced by resources and opportunities | (Child) "That’s why we’re all like mad when we go to recess, because we don’t have anything to slide on or to swing on." |
|  |  |  |  |  |  |  |
| **Enjoyment** | 1 | 4 | Lack of PA | Inclusion | Inclusive practices | (Child) "I know like she didn’t get to play all the innings." |
|  | 3 | 0 | Lack of resources | Equipment | Opportunity to participate in physical activity | (Adult) "But sometimes the kids just need to go outside. But if you go outside to our playground, it, no offense, it sucks. It’s terrible. They have nothing for those kids to do . . . are broken. It’s just bad." |
|  | 6 | 7 | Leadership | Role modeling | Enthusiastic leaders can convey enthusiasm | (Adult) "If we can get them excited, I think it’s going to carry over to the parents." |
|  | 1 | 9 | Acknowledgement | Incentive | Incentives for child engagement | (Adult) "So maybe we could entice some of the kids to stay if we’re not just doing what I will call schoolwork." |
|  | 3 | 1 | Structured programming | Variation | Variation to enhance students PA engagement | (Child) "I don't like running the mile every Friday in PE" |
|  | 7 | 8 |  | Inclusion | Considering specific needs of children | (Child) "I don't like running because it like messes with my asthma and stuff" |
|  |  |  |  |  |  |  |
|  | 2 | 1 |  | Physical activities | Participate in structured PA children enjoy | (Child) "I like playing dodgeball and playing basketball" |
|  | 3 | 1 |  | Sedentary behavior | Participate in frequent PA breaks to increase focus | (Child)"I don’t like sitting in class the whole time, because I get very bored. And I have, I'm really hyper, so I like PE class" |
|  | 1 | 0 | Physical discomfort | Screen time | Prolonged screen time has a negative impact on physical comfort | (Child) "Sometimes when I get home, my neck is really, really sore from the tablets in class." |
|  |  |  |  |  |  |  |
| **Policy, Systems, and Environment (PSE)** | 3 | 5 | Lack of resources | Personnel | Shortage of qualified personnel | (Adult) "I honestly can't tell you the last time that we had a true P.E. teacher" |
|  |  |  |  |  |  |  |
|  | 3 | 3 |  | Equipment | Poor availability and quality of playground equipment | (Adult) "…if you go outside to our playground, it, no offense, it sucks. It's terrible. They have nothing for those kids to do… it's just bad." |
|  | 0 | 1 |  | Nutrition | Poor nutritional quality of food choices | (Child) " We would always just have milk over and over and over again, you know. And we couldn’t like to have like no juice or, and the same whenever we have lunch." |
|  | 2 | 2 | PA policy/systems | Enhanced PA | Equal opportunities influence children behavior | (Adult) "I'll walk by the gym, and, you know, there's kids sitting on the bleachers" |
|  | 0 | 1 |  | Extended PA | Frequent PA opportunities influence child participation level | (Adult) "They don’t even do [PE] every day" |
|  | 6 | 7 |  | Expanded PA | Emphasize need for expanded PA opportunities | (Adult) "Sometimes the kids just need to go outside" |
|  | 4 | 2 |  | Time restrictions | Time balance between education and PA | (Adult) "Our schedules are tight… we have a block to teach our certain topic" |
|  | 1 | 1 |  | Screen time | Regulate screen time | (Adult) "When we do so much screentime, and it is phone time and iPad time and everything, they don’t know how to communicate with each other" |
|  | 1 | 0 |  | Lack of organization | Lack of organization impact PA participation | (Adult) "Hardly anybody would tell you where you're supposed to go or what you're supposed to do… so that was kind of aggravating." |
